# Supplementary material for: Mexican Biobank advances population and medical genomics of diverse ancestries
Source: Nature. 2023 Oct 11;622(7984):775–83. doi: 10.1038/s41586-023-06560-0 (PMC10600006; doi:10.1038/s41586-023-06560-0)
Supplement: Supplementary file 1 — Supplementary notes on the strengths and limitations of the MXB, sampling ascertainment in the MXB and an observation of gene–culture discordance in the MXB for ancestries and languages from the Americas; links to all software and R packages used in this study; Figs. 1–61 and Tables 1–9, providing further details of the dataset, and of the population genetic and complex trait analyses presented in the manuscript. [file 41586_2023_6560_MOESM1_ESM.pdf]

nature portfolio

https://doi.org/10.1038/s41586-023-06560-0

Supplementary information

Mexican Biobank advances population and medical genomics of diverse ancestries

In the format provided by the authors and unedited

Supplementary Information

Mexican Biobank advances population and medical genomics of diverse ancestries

Mashaal Sohail[1, 2,3,#], María J. Palma-Martínez[1,*], Amanda Y. Chong[4,*], Consuelo D. Quinto-Cortés[1,*], Carmina Barberena-Jonas[1], Santiago G. Medina-Muñoz[1], Aaron Ragsdale[1], Guadalupe Delgado-Sánchez[5], Luis Pablo Cruz-Hervert[5,6], Leticia Ferreyra-Reyes[5], Elizabeth Ferreira-Guerrero[5], Norma Mongua-Rodríguez[5], Sergio Canizales-Quintero[5], Andrés Jiménez-Kaufmann[1], Hortensia Moreno-Macías[7,8], Carlos A. Aguilar-Salinas[9], Kathryn Auckland[4], Adrián Cortés[10], Víctor Acuña-Alonzo[11], Christopher R. Gignoux[13], Genevieve L. Wojcik[14], Alexander G. Ioannidis[12], Selene L. Fernández-Valverde[1], Adrian V.S. Hill[4,15], María Teresa Tusié-Luna[7], Alexander J. Mentzer[4, 10], John Novembre[2,16], Lourdes García-García[5,#], Andrés Moreno-Estrada[1,#]

List of Supplementary Figures

Fig. S1. Map of Mexico with state labels.
Fig. S2. Number of individuals per state.
Fig. S3. Distribution of rural and urban samples in the MXB.
Fig. S4. Number of MXB individuals that speak Spanish, and Indigenous language or both.
Fig. S5. Number of MXB individuals from a rural or urban locality.
Fig. S6. PCA analysis of MXB with global reference panels.
Fig. S7. PCA analysis of MXB.
Fig. S8. PCA analysis of MXB and NMDP grouped by Mesoamerican regions.
Fig. S9. PCA analysis of MXB and NMDP grouped by Indigenous culture.
Fig. S10. Admixture analysis of MXB in principal component space of MXB and NMDP
Fig. S11. Admixture analysis of the MXB and global reference panels across K values.
Fig. S12. Cross validation errors from range of K values in Admixture analysis.
Fig. S13. Admixture analysis of MXB and NMDP.
Fig. S14. Origins of ancestries from West Africa in MXB.
Fig. S15. F_{ST} analysis of autosomes of MXB individuals grouped by their state.
Fig. S16. F_{ST} analysis of autosomes of MXB individuals grouped by their cultural region.
Fig. S17. F_{ST} analysis of autosomes of MXB individuals with high ancestries from the Americas grouped by their state.
Fig. S18. F_{ST} analysis of autosomes of MXB individuals with high ancestries from the Americas grouped by cultural region.
Fig. S19. UMAP analysis of MXB samples with 10 principal components.
Fig. S20. UMAP analysis of MXB samples with global reference panels.
Fig. S21. Gimbernat-Mayol et al., 2022 analysis polygon compositional plots of the MXB (3-10 sources, indicated by A0-A10 on vertex edges).
Fig. S22. Gimbernat-Mayol et al., 2022 analysis polygon compositional plots of the MXB and reference individuals (3-10 sources, indicated by A0-A10 on vertex edges).
Fig. S23. Gimbernat-Mayol et al., 2022 analysis polygon compositional plots of the MXB colored by Mesoamerican regions and references individuals (3-10 sources, indicated by A0-A10 on vertex edges).
Fig. S24. Origins of ancestries from the the Americas in MXB.
Fig. S25. asIBNe analysis results for ancestries from the Americas are visualized for 30 or 20 years per generation.
Fig. S26. Estimated effective population size for ancestries from Western Europe by Mesoamerican region in Mexico (30 years per generation).

Fig. S27. Estimated effective population size for ancestries from Western Europe by Mesoamerican region in Mexico (20 years per generation).
Fig. S28. Estimated effective population size for ancestries from West Africa by Mesoamerican region in Mexico (30 years per generation).
Fig. S29. Estimated effective population size for ancestries from West Africa by Mesoamerican region in Mexico (20 years per generation).
Fig. S30. Gelman-Rubin convergence plot for the AdmixtureBayes analysis.
Fig. S31. NROH and SROH distribution by state.
Fig. S32. SROH are correlated with ancestries in MXB.
Fig. S33. Distribution of ROH segments of different sizes for each Northern, Central, Southern and Southeastern state.
Fig. S34. Ancestries from the Americas as a function of birth year in rural and urban localities.
Fig. S35. Proportion of individuals that speak Spanish, an Indigenous language or both, as a function of birth year.
Fig. S36. Sample counts by birth year in the MXB.
Fig. S37. Birth year distribution by rural or urban regions.
Fig. S38. Distribution of ancestries from the Americas by rural and urban regions.
Fig. S39. Mutation burden in 1000G MXL individuals as a function of their ancestries from the the Americas, Western Europe, and West Africa.
Fig. S40. Rare mutation burden computed on WGS vs array SNPs.
Fig. S41. Schematic for testing prediction performance of polygenic scores computed in the MXB using MXB or UKB GWAS.
Fig. S42. A framework for the role of genetics and environment in creating genetic variation and variation in complex traits and disease risk used in this study.
Fig. S43. Average trait values per state visualized on the map of Mexico.
Fig. S44. Complex trait variation by latitude.
Fig. S45. Complex trait variation by longitude.
Fig. S46. Complex trait variation by altitude.
Fig. S47. Complex trait variation by sex.
Fig. S48. Complex trait variation by birth year.
Fig. S49. Complex trait variation by Indigenous heritage.
Fig. S50. Complex trait variation by rural or urban living environment.
Fig. S51. Variograms of complex traits.
Fig. S52. Correlation of educational attainment and income levels.
Fig. S53. An analysis of complex trait variation in Creatinine and blood pressure in the MXB.
Fig. S54. Mixed model results for ancestries from different global regions in the MXB.

Fig. S55. Mixed model results for two MDS axes reflecting genetic variation within the Americas.
Fig. S56. Mixed model results for sROH carried by an individual.
Fig. S57. Mixed model results for the polygenic score of each trait.
Fig. S58. Mixed model results for the environmental predictors for each trait.
Fig. S59. Complex trait architecture of Cholesterol related traits in the MXB.
Fig. S60. Linear regression of BMI on ancestry proxies inferred from Admixture.
Fig. S61. An analysis of BMI trait variation segmented in MXB individuals from rural or urban localities.

List of Supplementary tables

Table S1. Age and sex distribution in the Mexican Biobank.

Table S2. List of anthropometric, disease and lifestyle variables in the Mexican Biobank

Table S3. Estimation of admixture proportions by state in MXB.

Table S4. NMDP cultural groups and their designation into Mesoamerican regions.

Table S5. Number of ROH by local ancestry tracts.

Table S6: Case and control numbers for each trait.

Table S7: Lead SNPs and most significant variant found in GWAS catalogue.

Table S8. Assessment of polygenic score performance using MXB-GWAS or UKB-GWAS SNPs significant at $p < 0.1$.

Table S9. Assessment of polygenic score performance using MXB-GWAS or UKB-GWAS SNPs significant at $p < 10^{-8}$.

Supplementary Text

Strengths and limitations of MXB

We summarize the strengths and limitations of the MXB. Strengths include:1) large sample size; 2) the initial study population was representative of the Mexican civilian population at state and national levels in 2000; 3) we purposefully over-selected Indigenous populations 4) joint collection of socio-demographic, geographic, anthropometric, clinical, biochemical, and genetic data; 5) some of the clinical and biochemical markers have become public health problems in the present day (diabetes, obesity)[79] and thus this study may contribute to a better understanding of the epidemiology and causal factors; 6) representativeness of the data set allows correlation with diseases that were unknown at the time of the survey and that have now been characterized (e.g. COVID-19). Limitations include: 1) the study is cross-sectional, thus limiting causal inferences (e.g., age and glucose); 2) the health survey data (ENSA2000, see below) were collected in 2000 and thus extrapolation to the present should be done with caution; 3) the sample size limits detection of phenotypes that are rare; 4) there are many characteristics, factors, or variables that were not studied or are unknown that undoubtedly modify the significance of our observations; however, we are able to estimate the extent to which our models account for the variation in the observed data.

Sampling ascertainment in the MXB

We have a sampling bias towards younger individuals in the Mexican Biobank (Fig. S36). The age distribution in rural and urban areas is also not homogeneous (Fig. S37). Individuals in rural areas are significantly older than individuals in urban areas (Wilcoxon test W = 3221193, p-value = 6.518×10^{-7}), and we observe significantly higher ancestries from the Americas in rural areas compared to those in urban areas (Fig. S38, Wilcoxon test W = 3972985, p-value < 2.2×10^{-16}). Therefore, if there is any sampling bias, it is in the opposite direction of the signal we observe (we observe that younger individuals have higher proportions of Indigenous ancestries (Figs. S34, S35)); that is, it would make it such that older individuals have higher proportions of Indigenous ancestries as we have sampled more older individuals from rural areas where the Indigenous ancestries are more prevalent. Instead, we observe that younger individuals have higher proportions of Indigenous ancestries.

Gene-culture discordance in MXB for ancestries and languages from the Americas

We observe in the MXB that ancestries from the Americas are more frequently observed in younger individuals than in older individuals in the biobank (Fig. S34) and that this signal is largest in rural areas. We also observe that the proportion of individuals that speak an Indigenous language is lower in younger individuals compared to older individuals, providing an example of anti-correlated temporal trends in the abundance of Indigenous language use vs Indigenous genetic ancestry proportions (Fig. S35). Many linguists, artists, and human rights activists have been warning that Mexico is becoming increasingly monotone, and this is not because of the lack of Indigenous presence today but due to discrimination and repressive assimilation policies, whereby many Indigenous people believe it is better not to be heard speaking their Indigenous language in Mexico[80]. In this study, we similarly

observe an increasing presence of ancestries from the Americas going hand in hand with a decline in the passage of Indigenous languages.

Links to software and R packages used in this study
EIGENSOFT (v7.2.1): https://github.com/dReichLab/EIG
Smartpca (part of Eigensoft v7.2.1): https://github.com/chrchang/eigensoft/tree/master/POPGEN
ADMIXTURE (v1.3.0): https://dalexander.github.io/admixture/
UMAP (repository downloaded Dec 2021): https://github.com/lmcinnes/umap
Archetypal analysis (repository downloaded Nov 2022): https://github.com/AI-sandbox/archetypal-analysis
GNOMIX (repository downloaded Oct 2021): https://github.com/AI-sandbox/gnomix
maas-MDS (repository downloaded Nov 2021): https://github.com/AI-sandbox/maasMDS/
shapeit (v2.17): https://mathgen.stats.ox.ac.uk/genetics_software/shapeit/shapeit.html
RFMIX (v2): https://github.com/slowkoni/rfmix
PCAmask (20131203): https://mybiosoftware.com/tag/pcamask
AdmixtureBayes (repository downloaded Jan 2023): https://github.com/svendvn/AdmixtureBayes
Refined-ibd (17Jan20): https://faculty.washington.edu/sguy/asibdne/
Merge-ibd-segments (17Jan20): https://faculty.washington.edu/sguy/asibdne/
Beagle (25Nov19): https://faculty.washington.edu/sguy/asibdne/
asIBDNe (19Sept19): https://faculty.washington.edu/sguy/asibdne/
Plink (v1.9): https://www.cog-genomics.org/plink/1.9/
Variant Effect Predictor (ensemble-vep-release-104): https://www.ensembl.org/info/docs/tools/vep/index.html
Regenie (v3.1.3): https://rgcgithub.github.io/regenie/
FINEMAP (v1.3): http://www.christianbenner.com/
FUMA (v1.4.1): https://github.com/Kyoko-wtnb/FUMA-webapp/
KING (v2.2.8): https://www.kingrelatedness.com/
Mxmaps (2020.1.1.9000): https://www.diegovalle.net/mxmaps/
Genesis (release 3.17): https://www.bioconductor.org/packages/release/bioc/html/GENESIS.html
lme4qtl (development version): https://github.com/variani/lme4qtl

Supplementary figures

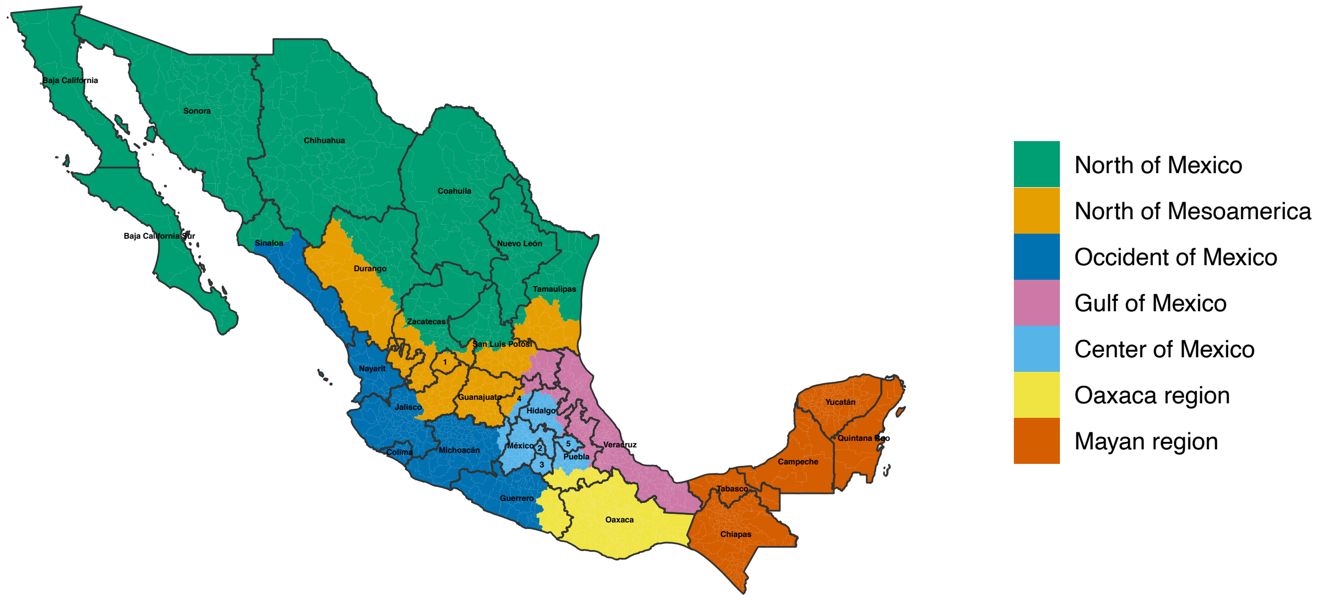

Fig. S1. Map of Mexico with labels per state. The MXB project collected and genotyped samples from all 32 states of Mexico.

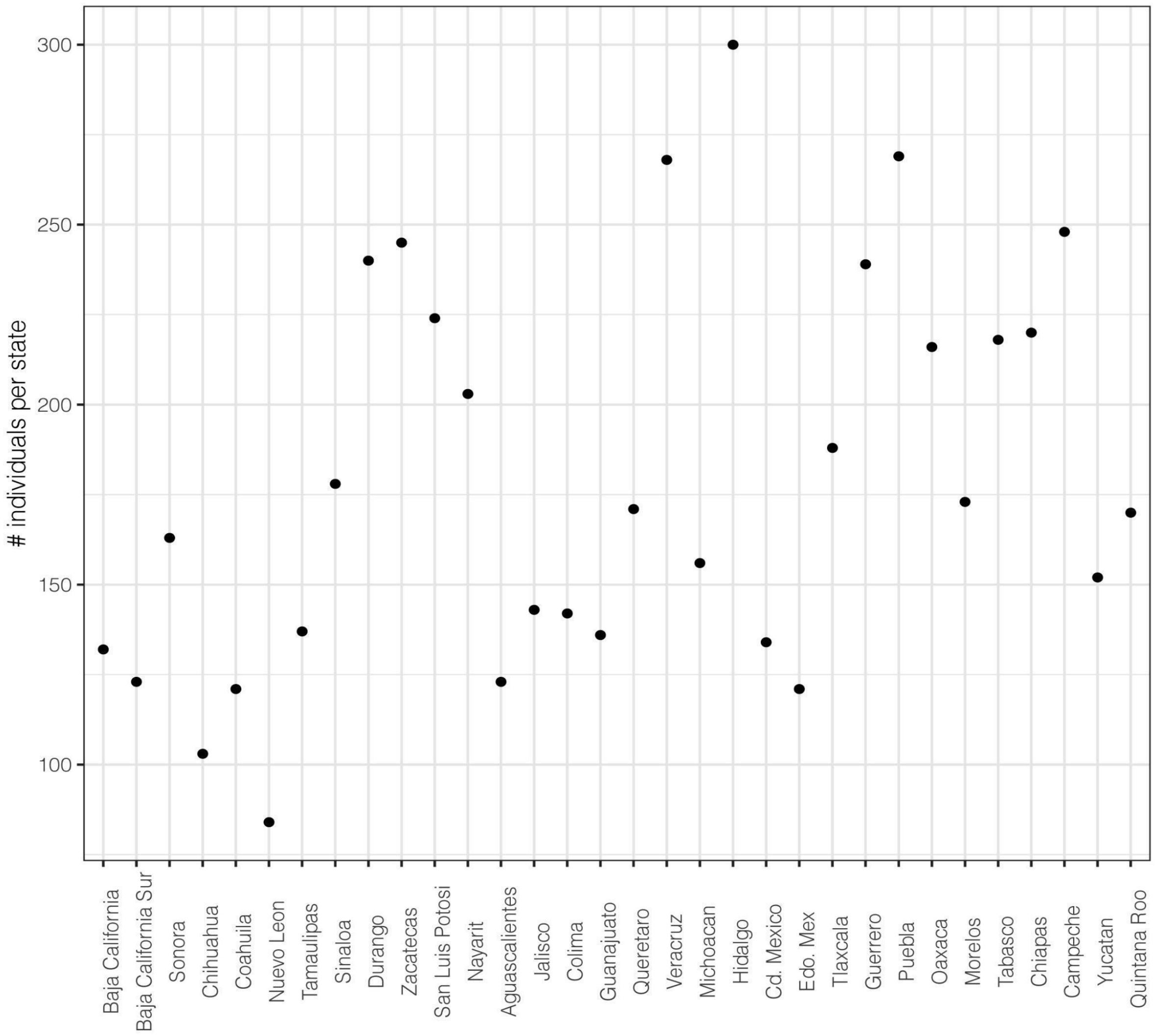

Fig. S2. Number of individuals per state in the MXB. Individuals in the MXB were sampled from the 32 Mexican states. The x-axis shows the states in Mexico and the y-axis shows the number of sampled individuals.

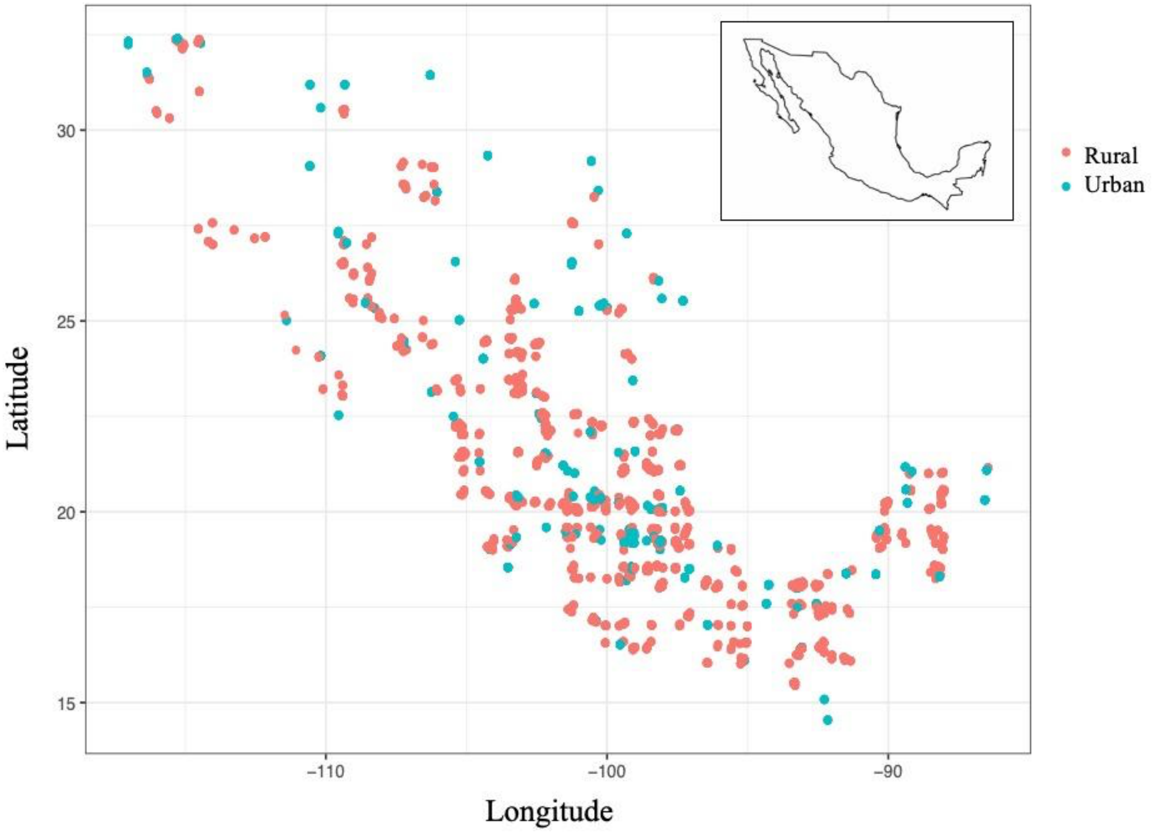

Fig. S3. Distribution of rural and urban samples in the MXB. Each dot represents the geographic location of a sampled locality with a minimum sample size of 5 individuals selected for genotyping. In total, the MXB covers 898 localities, 321 municipalities, and 32 states across Mexico. Rural localities are shown in orange and those in urban areas in blue. Most of the localities belong to rural areas.

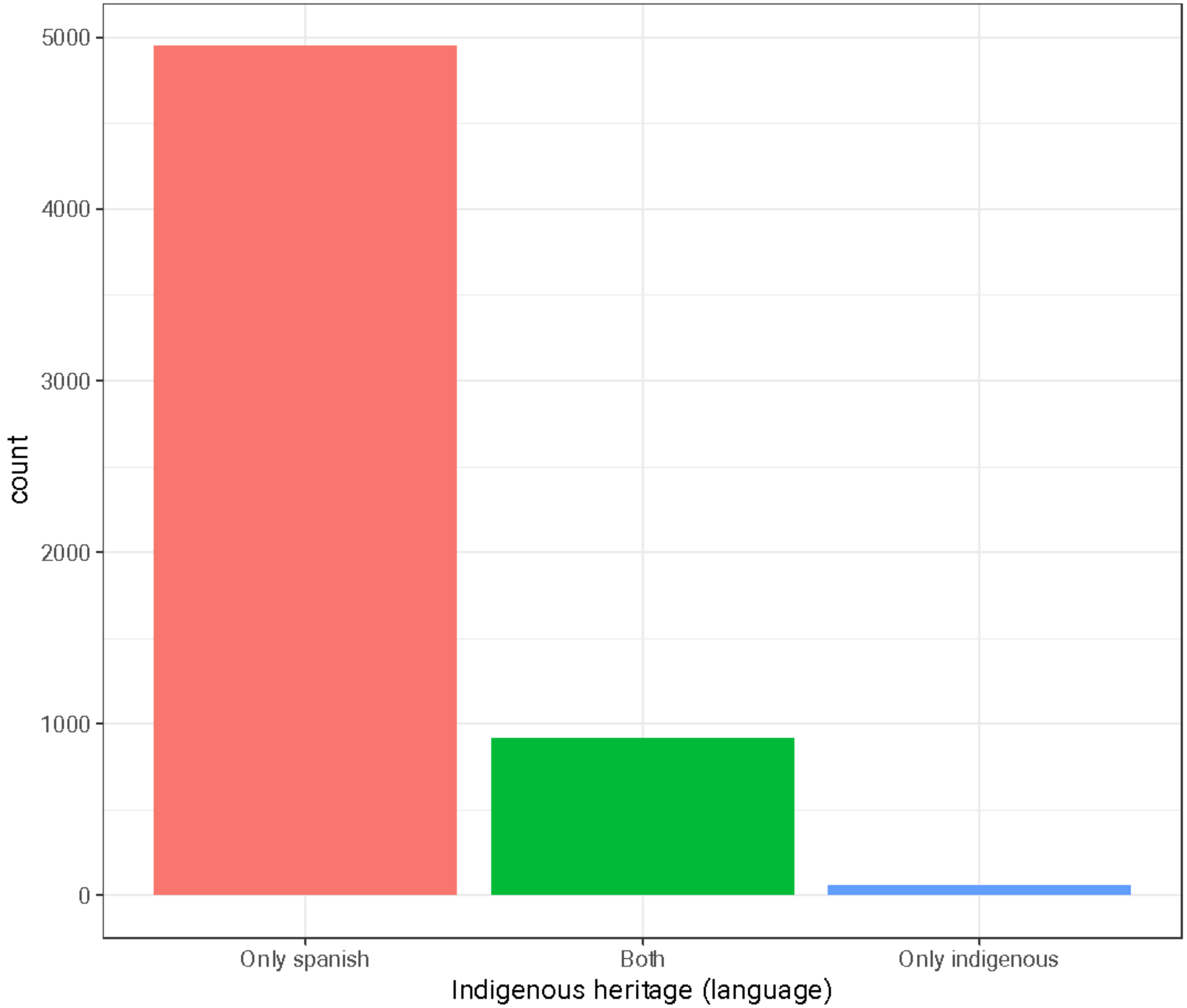

Fig. S4. Number of MXB individuals that speak Spanish, an Indigenous language, or both. Spanish speakers are shown in orange, those that speak both in green, and those that speak only an Indigenous language in blue.

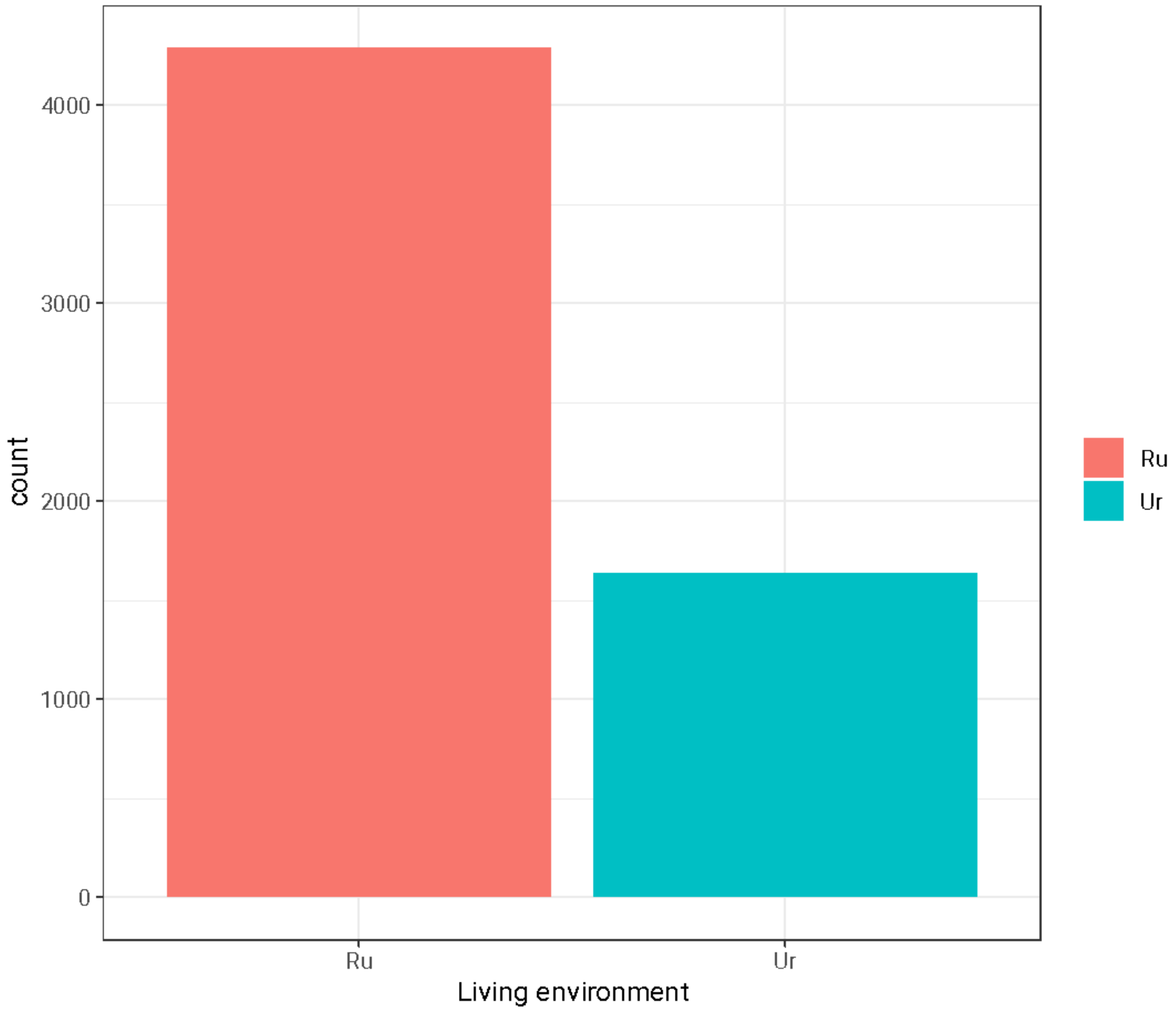

Fig. S5. Number of MXB individuals from a rural or urban locality. Rural localities are shown in orange, and urban localities in blue. Most of the individuals in the MXB live in a rural area.

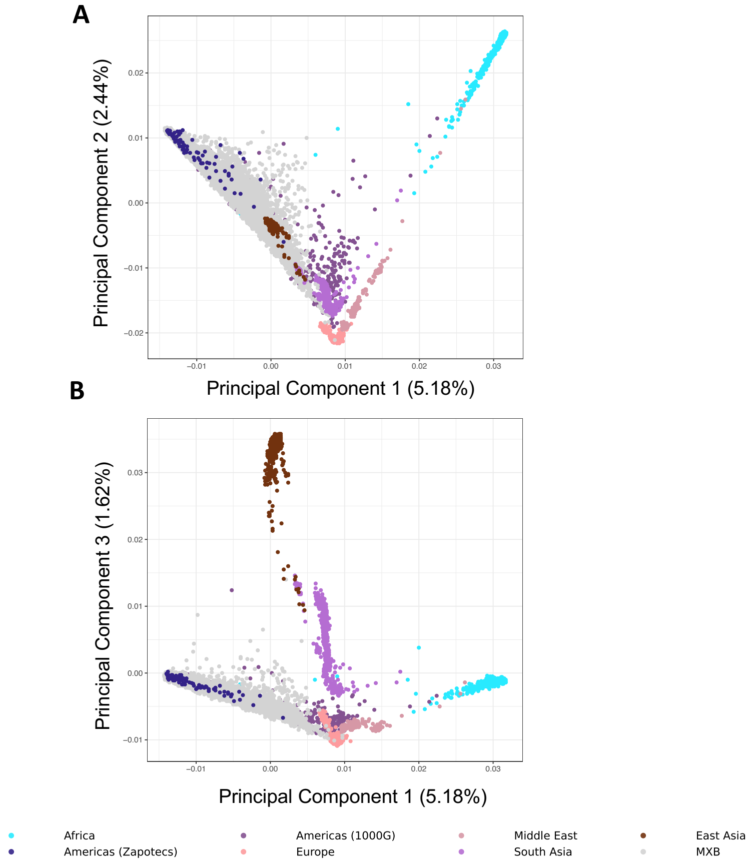

Fig. S6. PCA analysis of MXB with global reference panels. A) PCA analysis showing principal component one (PC1) in the x-axis and principal component two (PC2) in the y-axis. B) PCA analysis showing PC1 in the x-axis and PC3 in the y-axis. Predominant ancestry-proxy clusters inferred from Admixture are used to color present-day individuals used in PCA space solely to help with visualization (in groupings used by the reference datasets). Most of the MXB individuals lie in a cline between living Europeans and Indigenous Americans. We observe a pull towards present-day Africans reflecting the history of the trans-Atlantic slave trade to Mexico. Americas (Zapotecs) refers to Indigenous Zapotec individuals from Oaxaca, Mexico collected as part of the PAGE study. The other reference individuals are from the 1000 Genomes and HGDP studies and are the same as in Fig. 1.

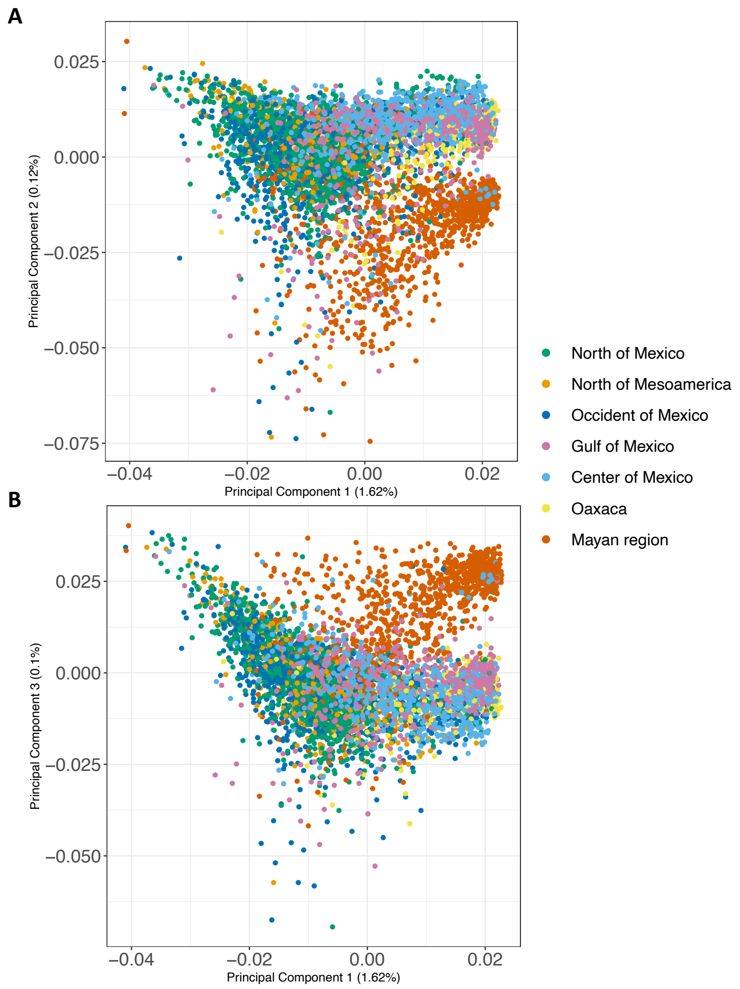

Fig. S7. PCA analysis of MXB. MXB colored by Mesoamerican region. A) PCA of the MXB showing principal component one (PC1) in the x-axis and principal component two (PC2) in the y-axis. B) PCA of the MXB showing principal component one (PC1) in the x-axis and PC3 in the y-axis. Individuals in the MXB show population substructure highlighting the separation of the Mayan region from the rest of the regions in both panels.

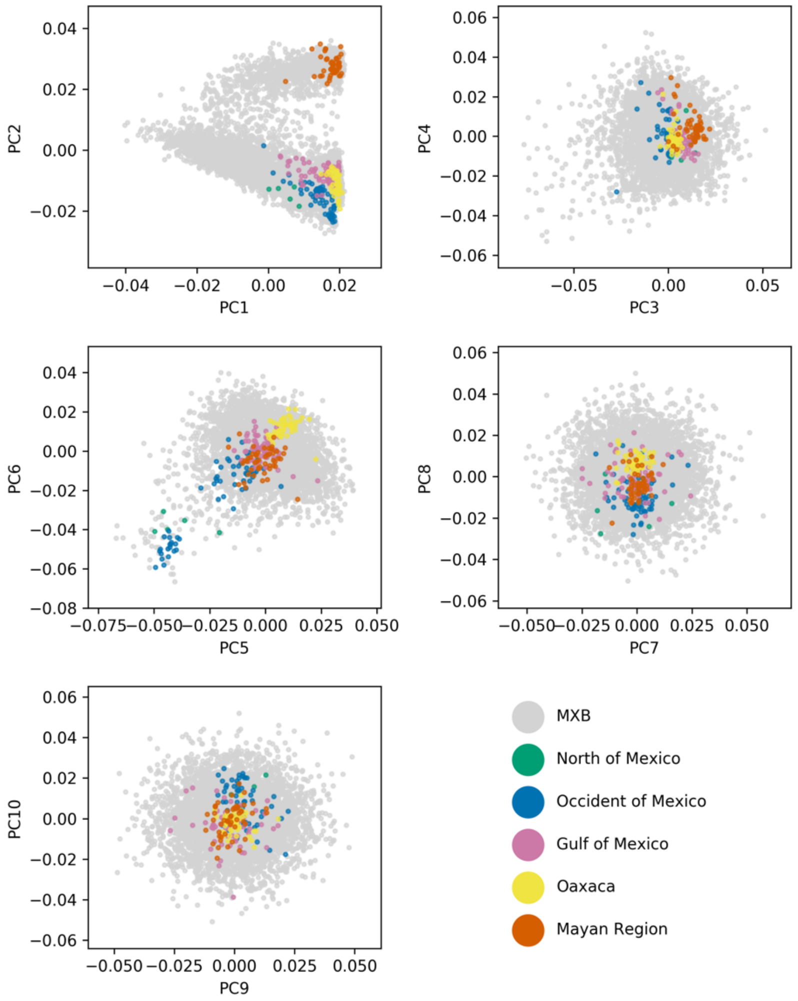

Fig. S8. PCA analysis of MXB and NMDP grouped by Mesoamerican regions. MXB is shown in grey and NMDP is colored by Mesoamerican region as shown in Table S4. The first 10 principal components (PCs) are shown. Each panel shows two of them.

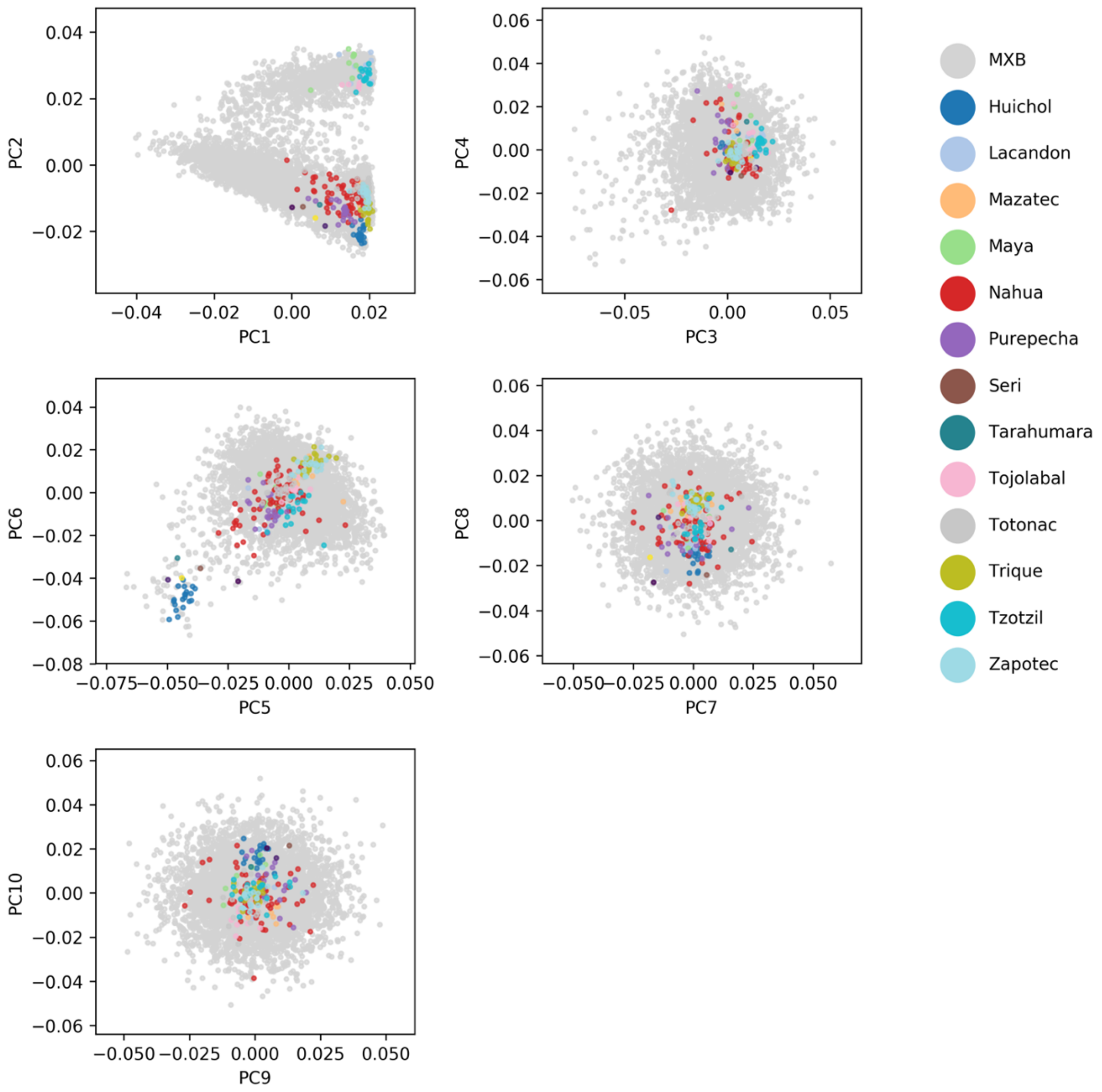

Fig. S9. PCA analysis of MXB and NMDP grouped by Indigenous culture. Plots are equivalent to those found in Fig. S8. NMDP individuals are classified according to the culture the individuals self-identify with[12].

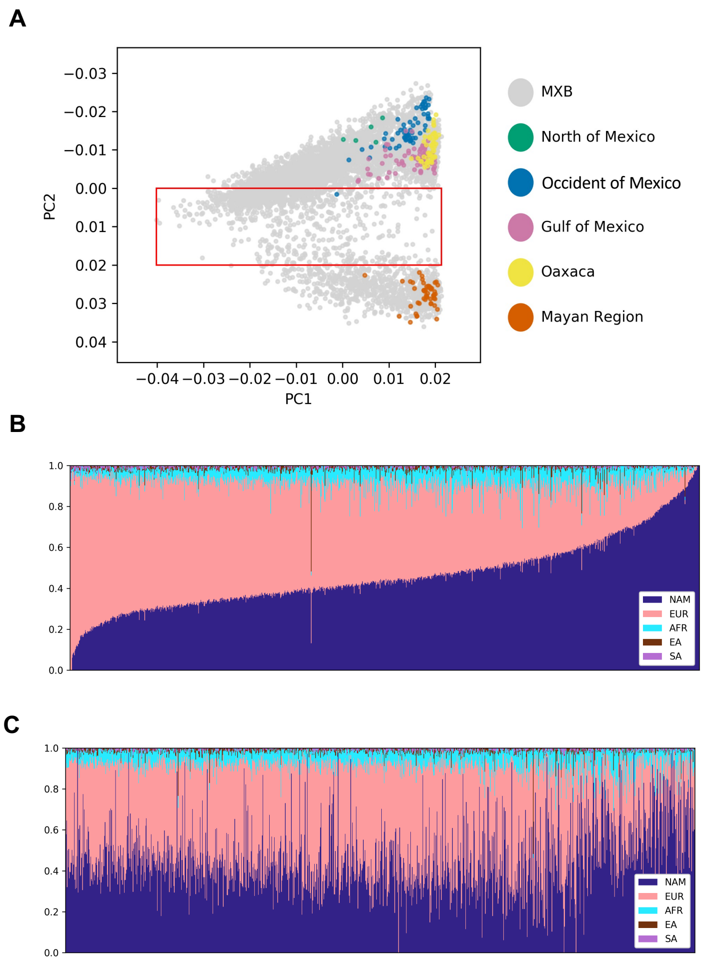

Fig. S10. Admixture analysis of MXB in a principal component space defined by MXB and Indigenous individuals from the NMDP. (**A**) PCA analysis of MXB samples with Indigenous groups from NMDP[12]. Indigenous groups were divided into the Mesoamerican regions. A red box indicates the samples selected for parts (B) and (C). (**B**) Admixture analysis of MXB samples sorted by principal component 1. This axis captures the propotion of ancestries from the Americas, with more Indigenous ancestries found moving rightwards. (**C**) Admixture analysis of MXB samples sorted by principal component 2. This axis captures variation within ancestry from the Americas and does not result in any apparent pattern at the scales of ancestries from different continents.

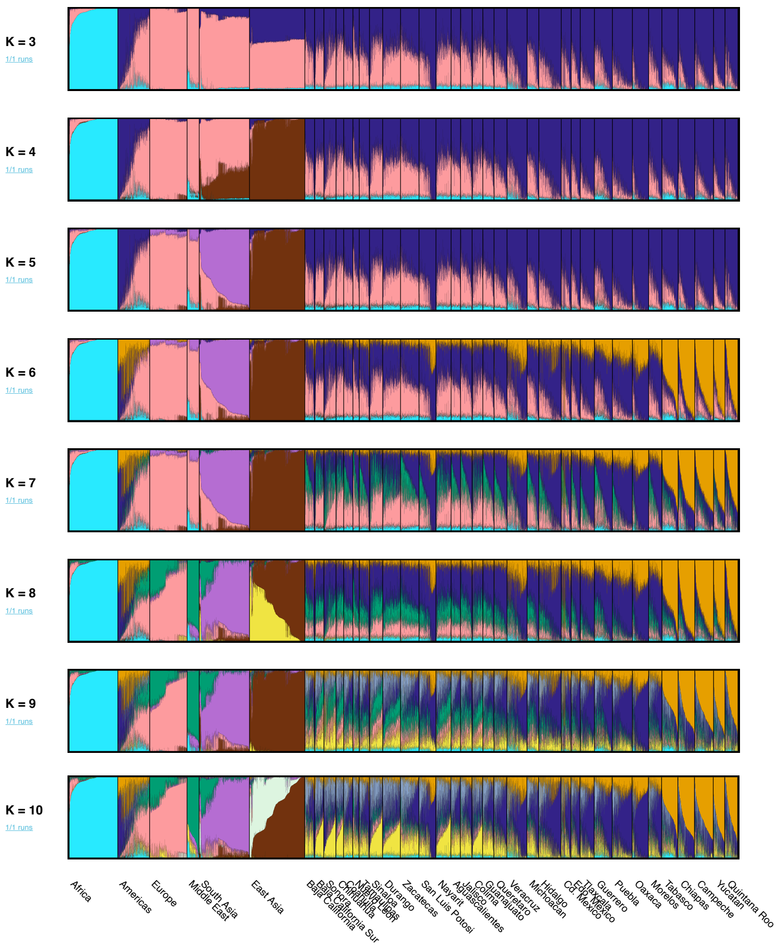

Fig. S11. Admixture analysis of the MXB and global reference panels across K values. MXB individuals are grouped by state in all plots. Each row shows the result at a different K value, from K=3 to K=10.

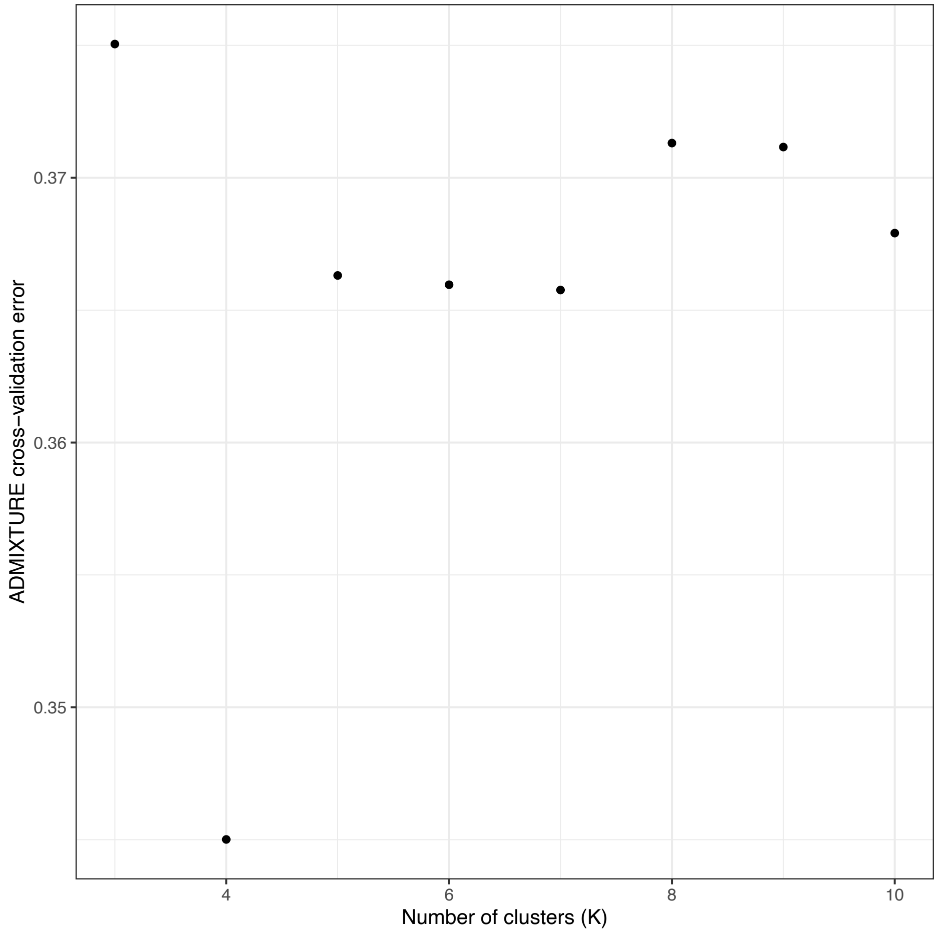

Fig. S12. Cross-validation errors for range of K values in Admixture analysis. Cross-validation errors correspond to the analysis shown in Fig. S11. The lowest cross validation error is seen at K=4.

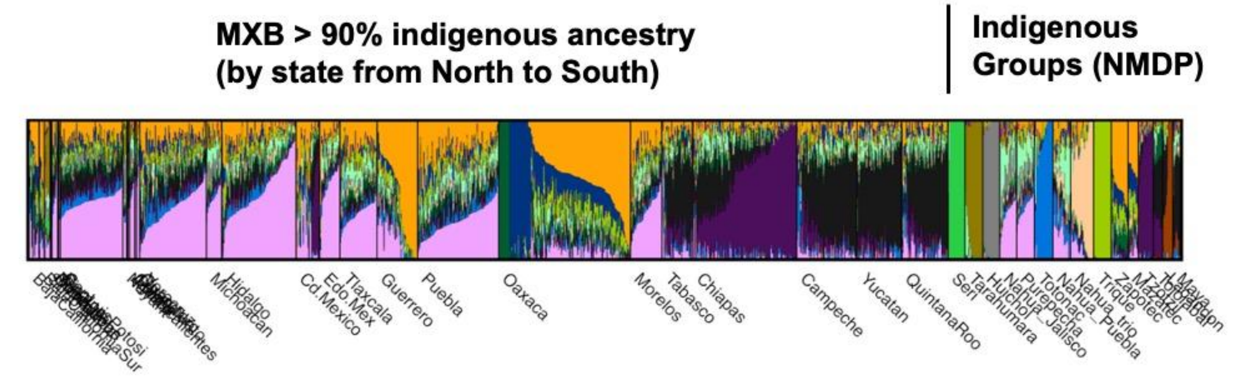

Fig. S13. Admixture analysis of MXB and NMDP. Only individuals that had significant ancestries from the Americas were chosen for this analysis from the analysis in Figure 1. An admixture analysis was performed with Indigenous groups from the NMDP (K=15).

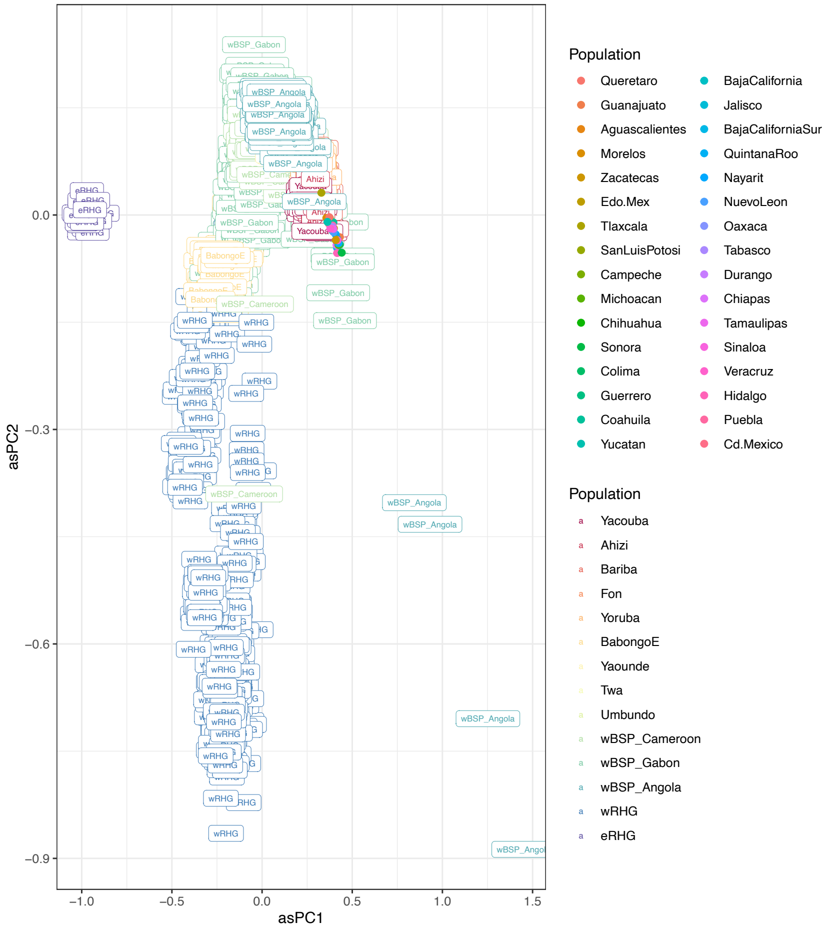

Fig. S14. Origins of ancestries from West Africa in MXB. Segments of the MXB genome inferred to have ancestries from Africa were employed in an ancestry-specific PCA in a reference space computed using samples from different groups in Africa collected by previous studies[21]. These axes show variation within ancestries from Africa, the third most predominant source of ancestries in Mexico. Each state is plotted at the average values for asPC1 and asPC2 of each of its individuals. Individuals from each state cluster with groups from West Africa. The groups from Africa are labelled according to their cohort labels in previous work[21]. wRHG and eRHG correspond to western and eastern rainforest hunter-gatherers, respectively. wBSP correspond to Bantu-speaking populations from Western Central Africa.

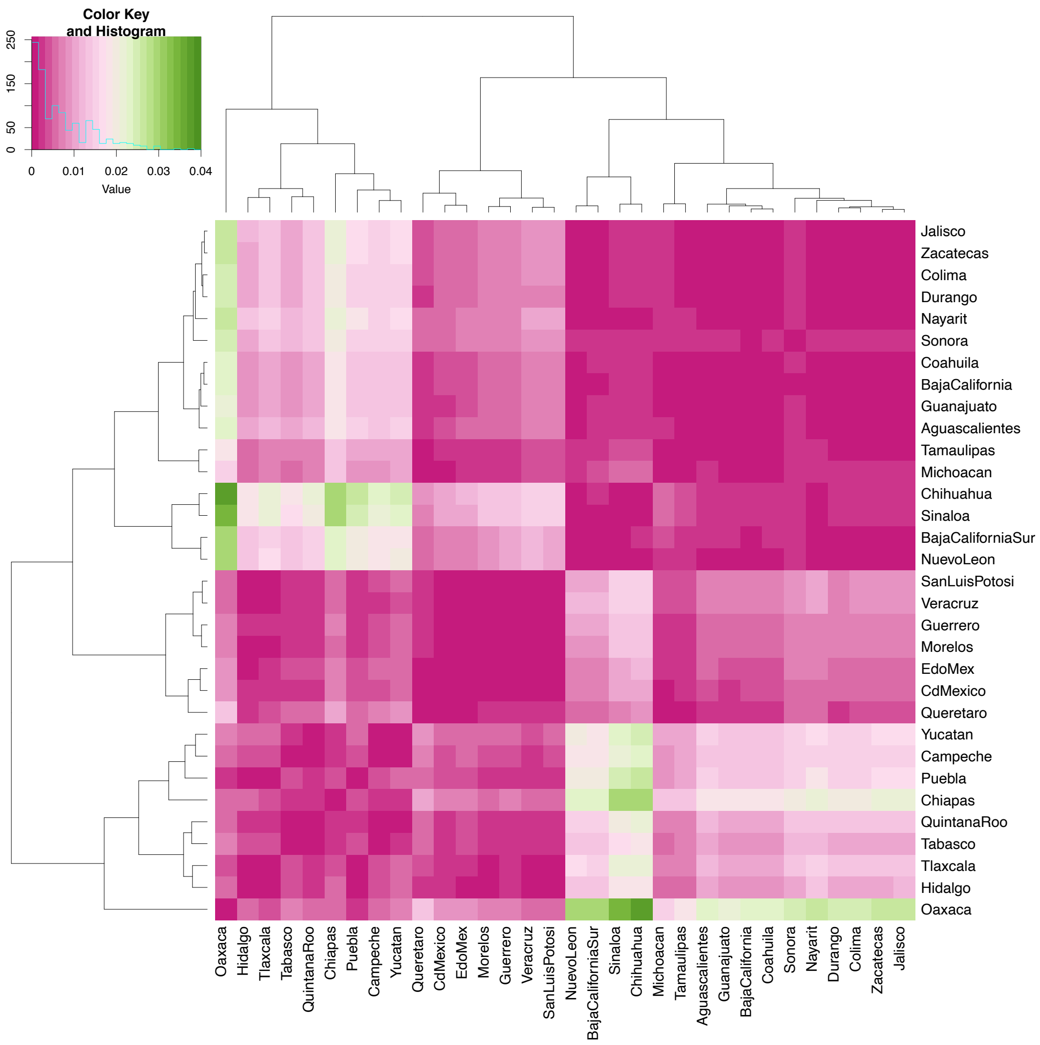

Fig. S15. F_{ST} analysis of autosomes of MXB individuals grouped by their state. The heatmap represents the results of the pairwise F_{ST} analysis. The highest values of differentiation are shown in green. The largest genetic differentiation is seen along a north-to-southeast cline.

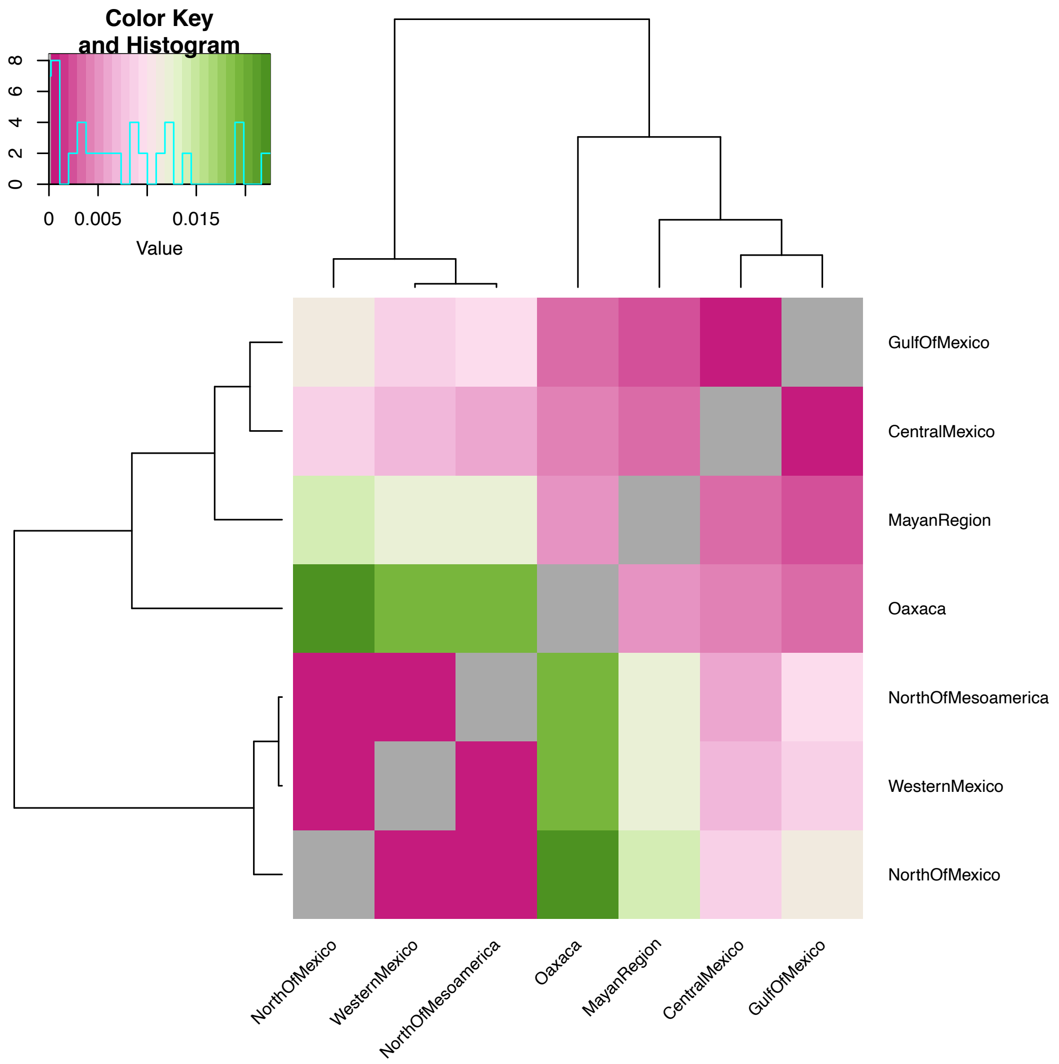

Fig. S16. F_{ST} analysis of autosomes of MXB individuals grouped by their cultural region. The heatmap represents the results of the pairwise F_{ST} analysis. The highest values of differentiation are shown in green. Zero F_{ST} values are shown in gray to distinguish them from very small Fst values (dark pink). The largest genetic differentiation is seen along a north-to-southeast cline.

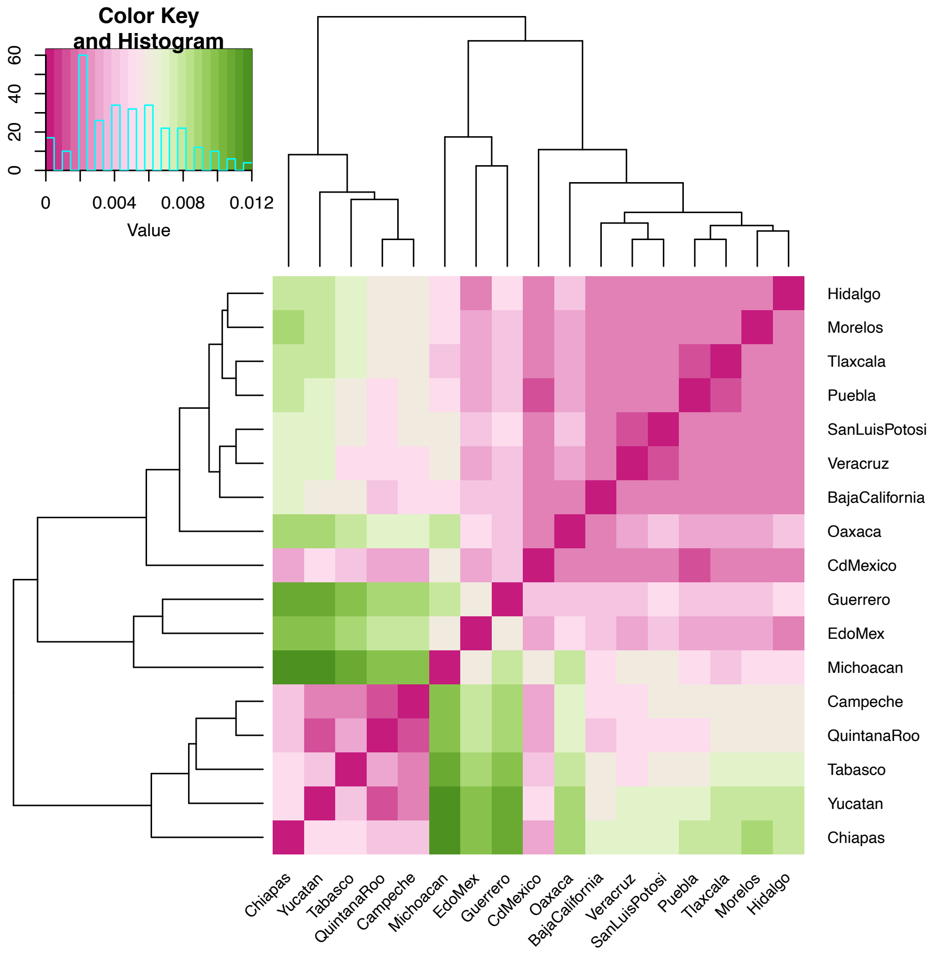

Fig. S17. F_{ST} analysis of autosomes of MXB individuals with high ancestries from the Americas grouped by their state. The heatmap represents the results of the pairwise F_{ST} Analysis. Only individuals with ≥ 90% proportion of ancestry from the Americas (inferred using Admixture) were analyzed. Only states with a minimum of 10 individuals are shown. The highest values of differentiation are shown in green. States in the Mayan region (Chiapas, Tabasco, Yucatan, Quintana Roo and Campeche) show the largest F_{ST} values with states in other regions.

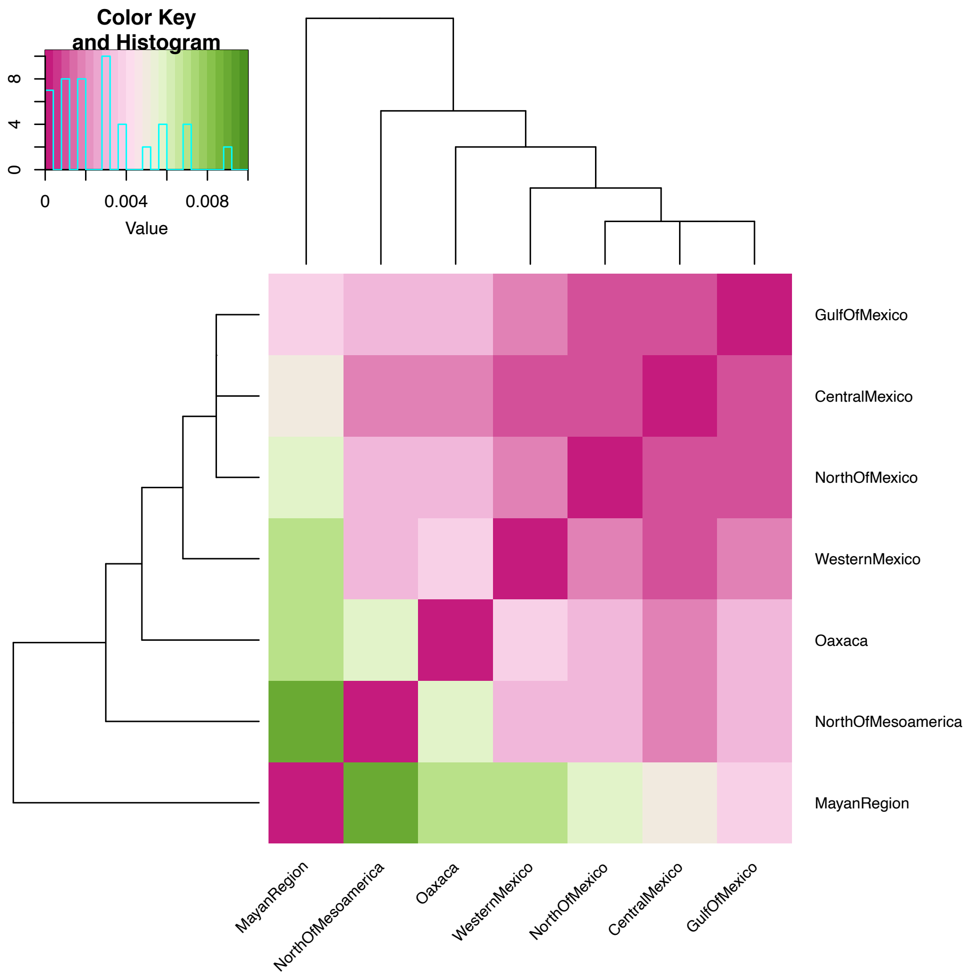

Fig. S18. F_{ST} analysis of autosomes of MXB individuals with high ancestries from the Americas grouped by cultural region. The heatmap represents the results of the pairwise F_{ST} Analysis. Only individuals with ≥ 90% proportion of ancestry from the Americas (inferred using Admixture) were analyzed. The highest values of differentiation are shown in green. The Mayan region shows the largest F_{ST} values with the other regions.

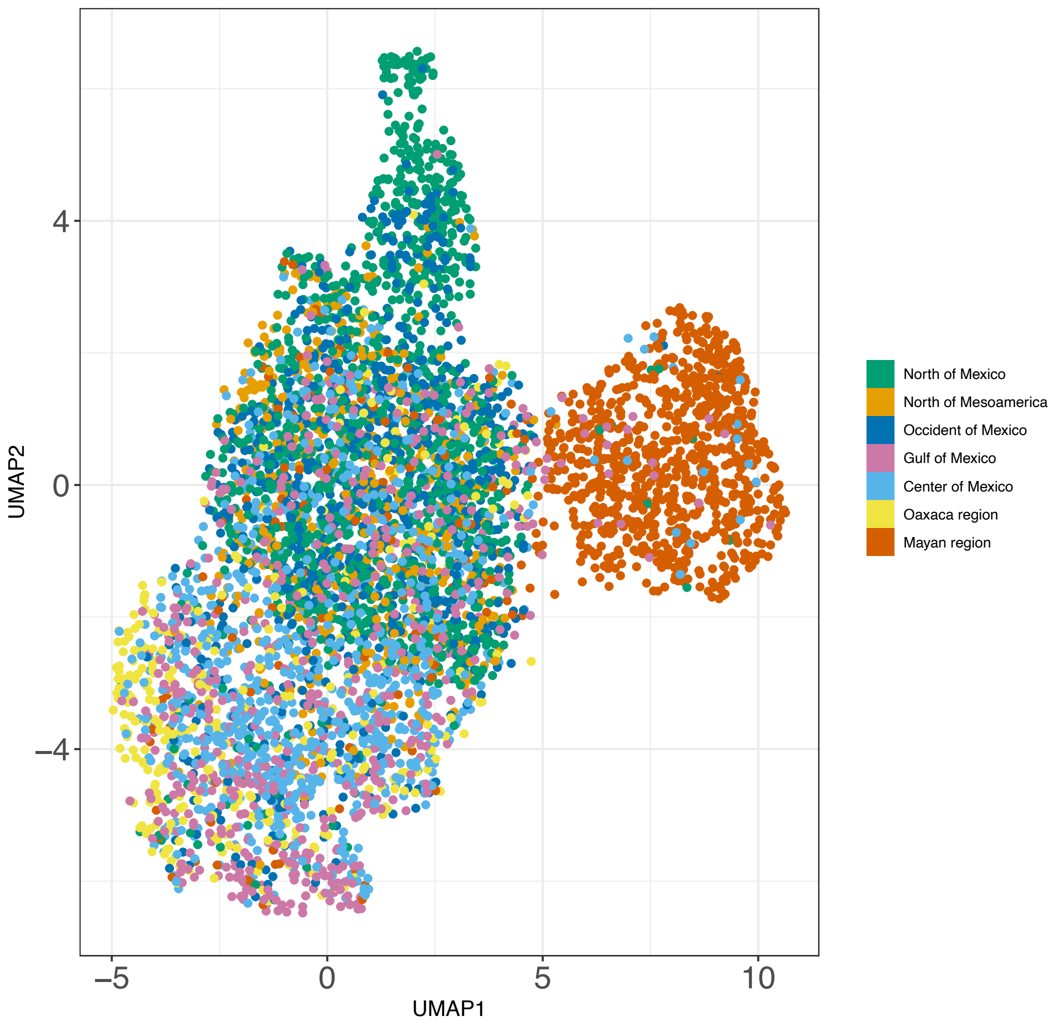

Fig. S19. UMAP analysis of MXB samples with 10 principal components. MXB colored by Mesoamerican region. UMAP1 axis shows the relative differentiation of the Mayan region from the rest.

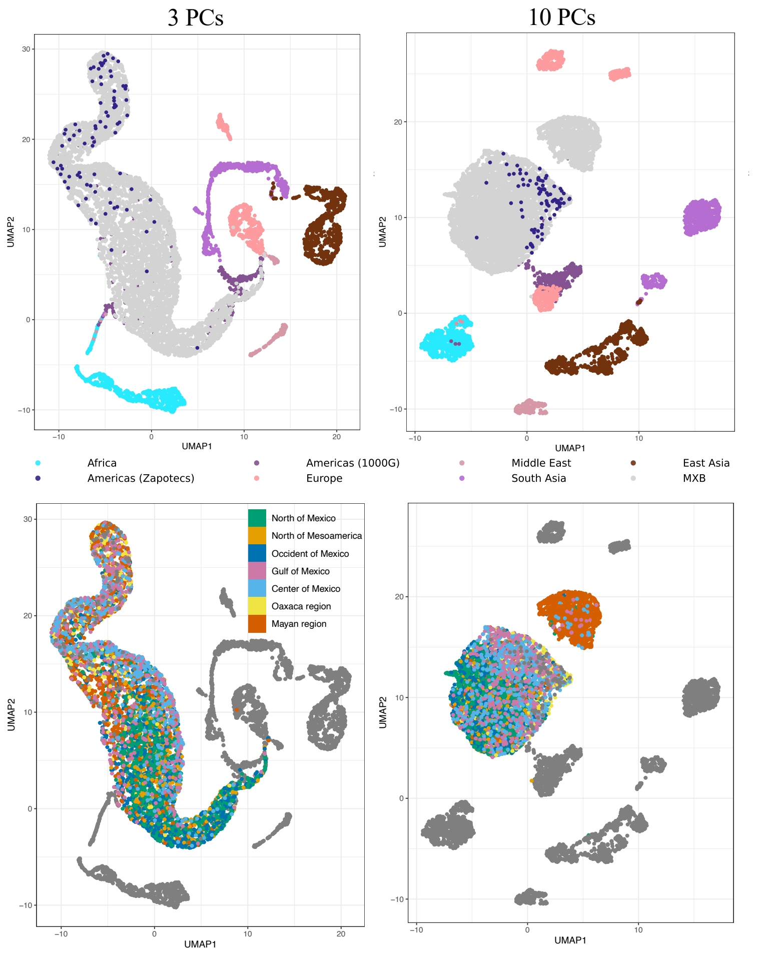

Fig. S20. UMAP analysis of MXB samples with global reference panels. Figures in the top row show global references in colors and MXB in gray. Figures in the bottom row show only the MXB colored by Mesoamerican region and global references in gray. The left column shows UMAP analysis with 3 principal components. The right column shows UMAP analysis with 10 principal components.

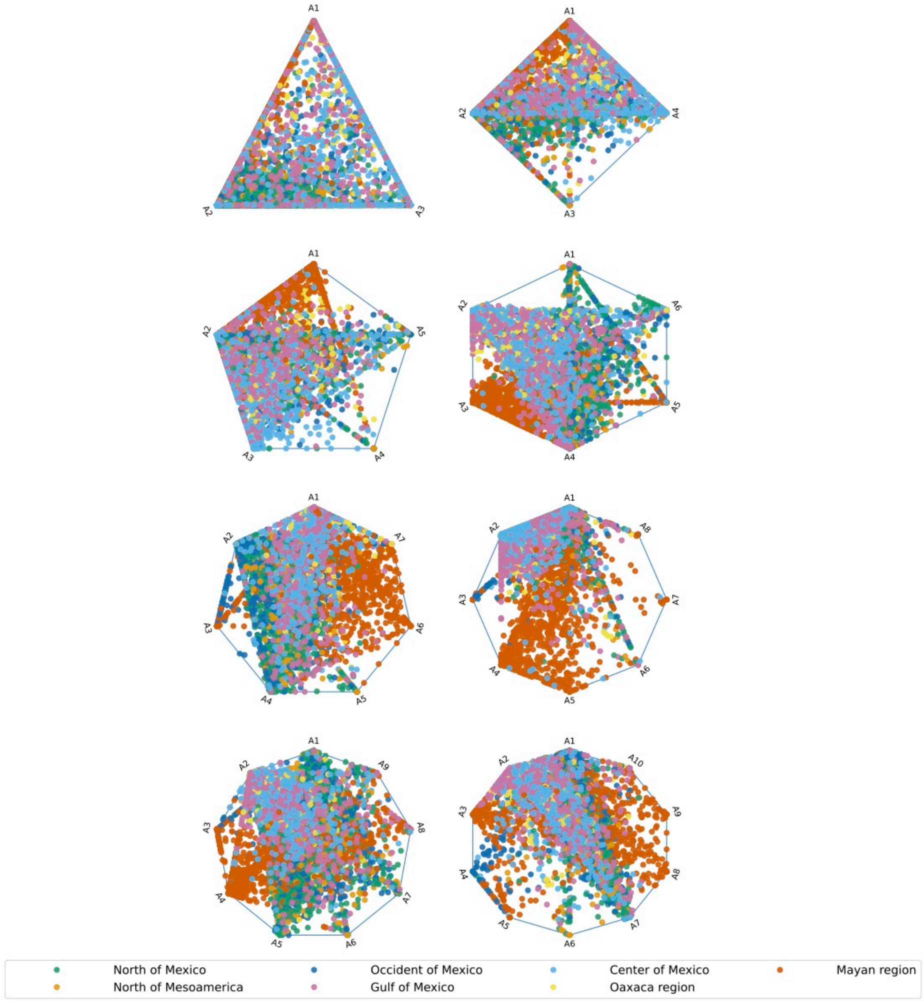

Fig. S21. Gimbernat-Mayol et al, 2022[5] analysis polygon compositional plots of the MXB (3-10 sources, indicated by A0-A10 on vertex edges). Each panel represents a compositional plot inferred with 3 sources (top left) up to 10 sources (bottom right). Points that fall on a vertex represent individuals whose ancestry derives from a single source. Points on the edges or diagonals represent individuals whose ancestry likely derives from the two sources on the extremes of the edge or diagonal. The rest of the points represent individuals whose ancestry derives from more than two sources. Most of the points that represent MXB samples fall inside the polygon between the different sources. The different colors represent the Mesoamerican regions. The Mayan region is represented by one or more sources. Individuals from the Mayan region tend to cluster together. The rest of the sources are shared by individuals from other regions.

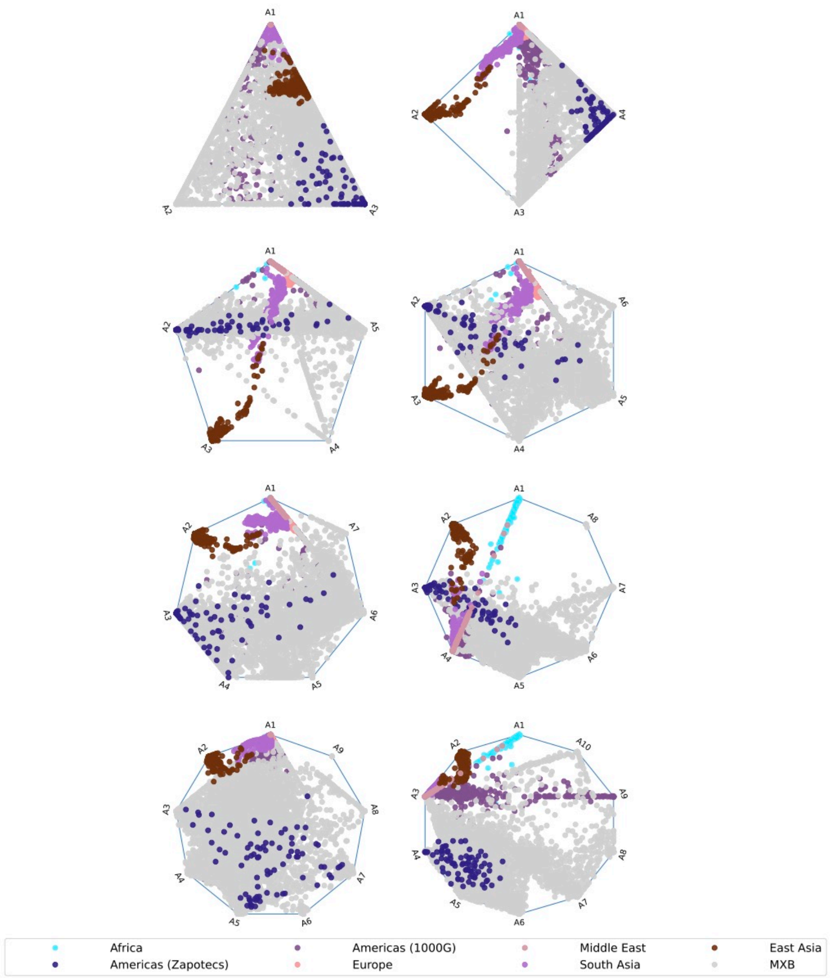

Fig. S22. Gimbernat-Mayol et al., 2022[5] analysis polygon compositional plots of the MXB and reference individuals (3-10 sources, indicated by A0-A10 on vertex edges). Each panel represents a compositional plot inferred with 3 sources (top left) up to 10 sources (bottom right). As mentioned in Gimbernat-Mayol et al., 2022; points that fall on a vertex represent individuals whose ancestry derives from a single source. Points on the edges or diagonals represent individuals whose ancestry derives from the two sources on the extremes of the edge or diagonal. The rest of the points represent individuals whose ancestry derives from more than two sources. Individuals from a continent tend to cluster near a vertex, often overlap with individuals from other continents and usually are seen as a gradient following a diagonal between vertices. The diversity found in the MXB is represented by multiple sources while individuals from South Asia, Europe, the Middle East and Africa tend to share the same source(s). At higher K values, individuals from Africa begin to be represented by their own source. Individuals from the Americas share sources along with the MXB individuals.

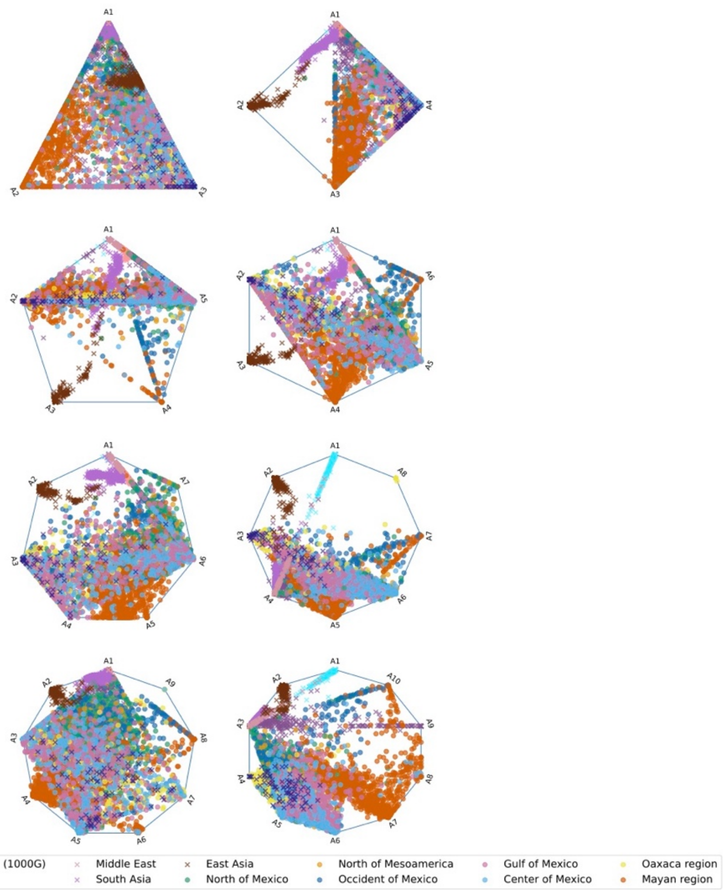

Fig. S23. Gimbernat-Mayol et al., 2022[5] analysis polygon compositional plots of the MXB colored by Mesoamerican regions and references individuals (3-10 sources, indicated by A0-A10 on vertex edges). To further assess the multiple sources defined by the MXB individuals (Fig. S22), we visualize the analysis in Fig. S22 with the MXB individuals colored by Mesoamerican region. Each panel represents a compositional plot inferred with 3 sources (top left) up to 10 sources (bottom right). Individuals from a continent tend to cluster near a vertex, often overlap with individuals from other continents and usually are seen as a gradient following a diagonal between vertices. The diversity found in the MXB is represented by multiple sources while individuals from South Asia, Europe, the Middle East and Africa tend to share the same source(s). At higher K values, individuals from Africa begin to be represented by their own source. Individuals from the Americas share sources along with the MXB individuals. For several regions, individuals from the same region (for example, the Mayan region) are represented by several sources, reflecting the diversity of ancestry variation within Mesoamerican regions. Individuals from different Mesoamerican regions also overlap in the results reflecting shared demographic histories and migrations among them. There are individuals from the Mayan region that tend to cluster mostly together, but even here we see overlap with individuals from the Gulf of Mexico and Central Mexico. At K=10, individuals from Oaxaca from the MXB and the reference panel (PAGE Zapotecs from Oaxaca) are mostly represented by the same source.

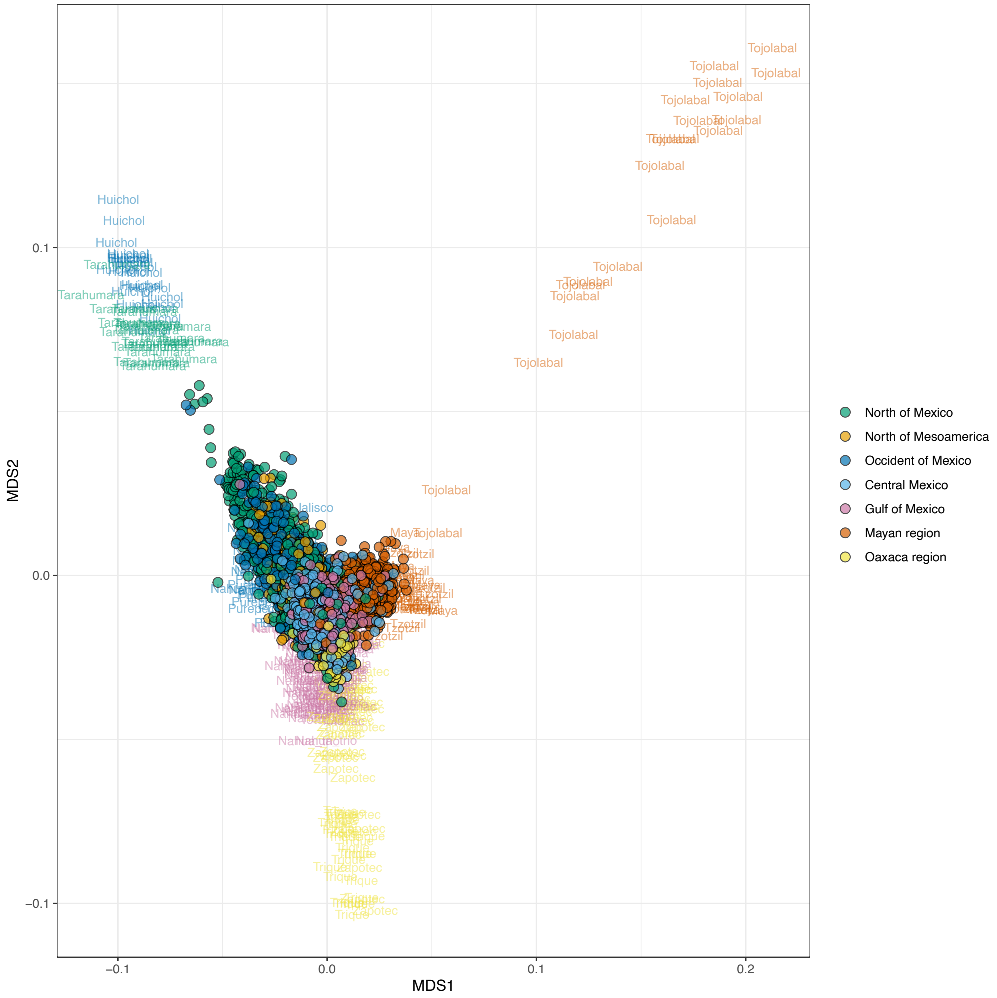

Fig. S24. Origins of ancestries from the Americas in MXB. Segments of the MXB genome inferred to have ancestries from the Americas were employed in multidimensional scaling (MDS) using MAAS-MDS in a reference space computed using samples from Indigenous groups collected as part of NMDP (see methods). These MDS axes will be used in the complex trait modelling section of the manuscript to study the role of fine-scale ancestry variation in complex trait variation.

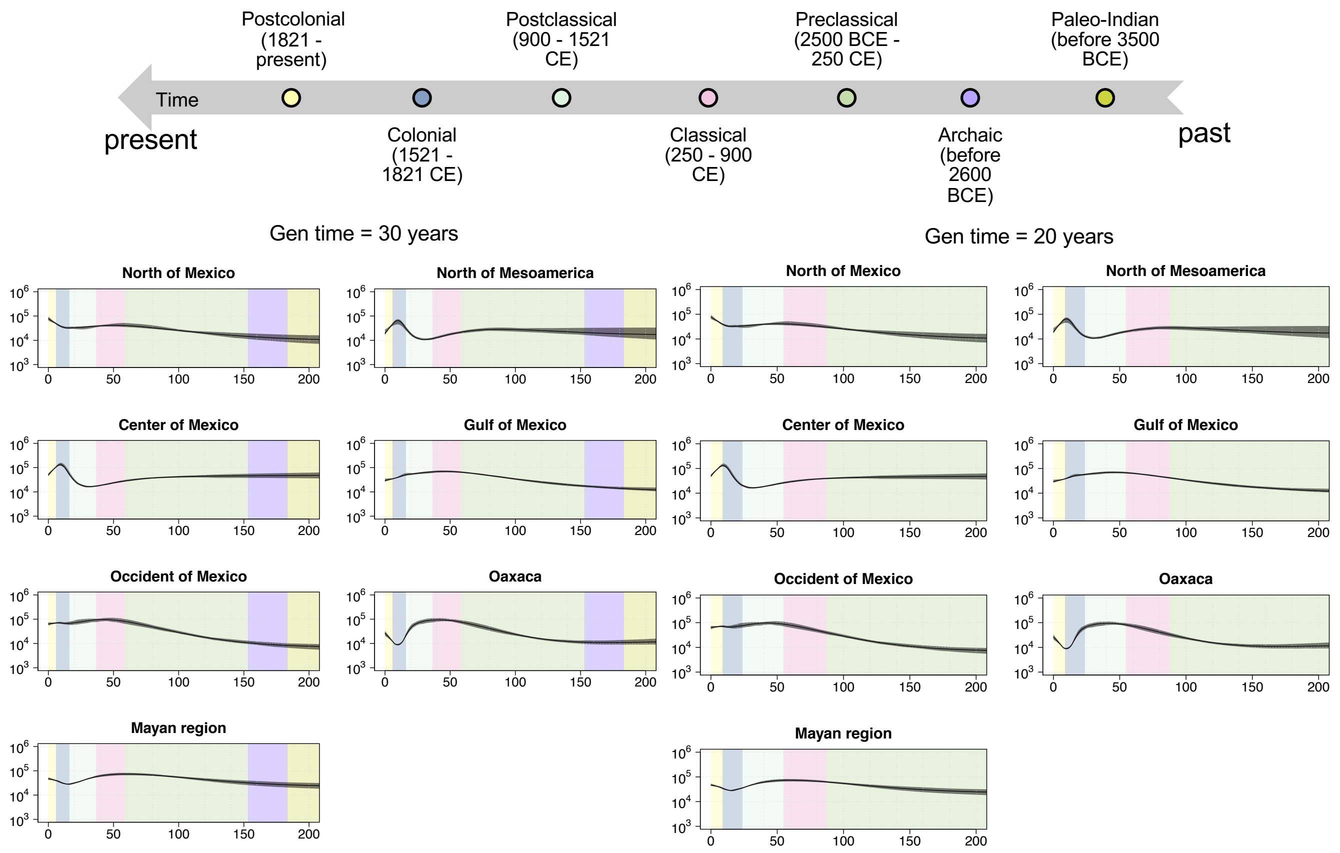

Fig. S25. ***asIBDNe*** **analysis results for ancestries from the Americas are visualized for 30 or 20 years per generation.** Effective population size changes were inferred for each Mesoamerican region using ancestry-specific IBD tracts. Different histories of the ancestries from the Americas are shown by Mesoamerican region. The figures on the left are the visualizations for 30 years per generation while the figures on the right are the visualization for 20 years per generation. The panels are colored by chronology (see the timeline in the upper part of the figure). Each panel represents a different region of Mexico. The solid black lines show estimated ancestry-specific effective population sizes, and the gray regions show 95% bootstrap confidence intervals.

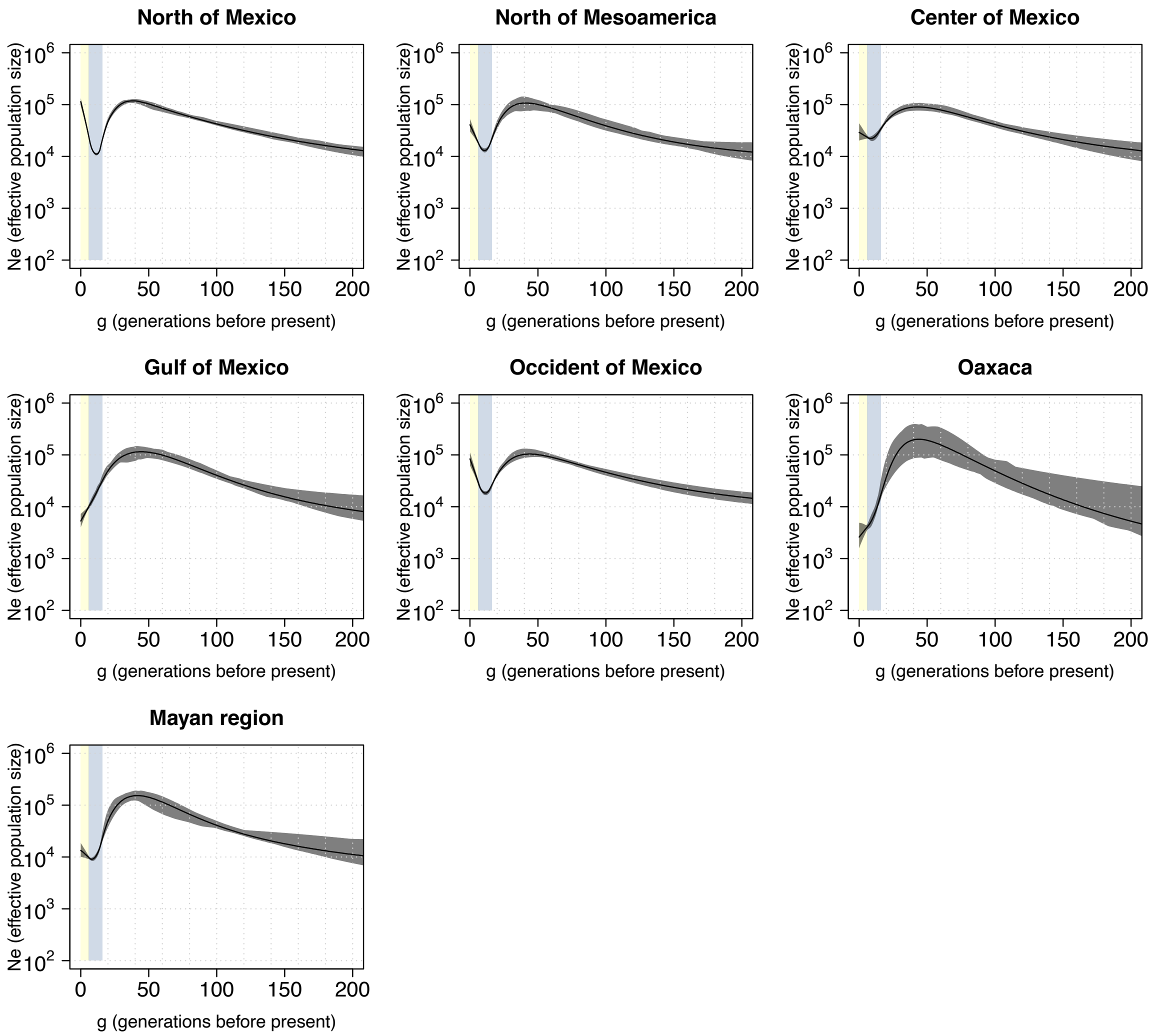

Fig. S26. Estimated effective population size for ancestries from Western Europe by Mesoamerican region in Mexico (30 years per generation). Effective population size changes were inferred for each Mesoamerican region using ancestry-specific IBD tracts. Each panel represents a different region of Mexico. The x-axis represents the number of generations before the present and the y-axis the effective population size. The colors in the panels represent colonial (purple) and postcolonial (light grey) periods (see timeline in the upper part of figure S25). The solid black lines show estimated ancestry-specific effective population sizes, and the gray regions show 95% bootstrap confidence intervals.

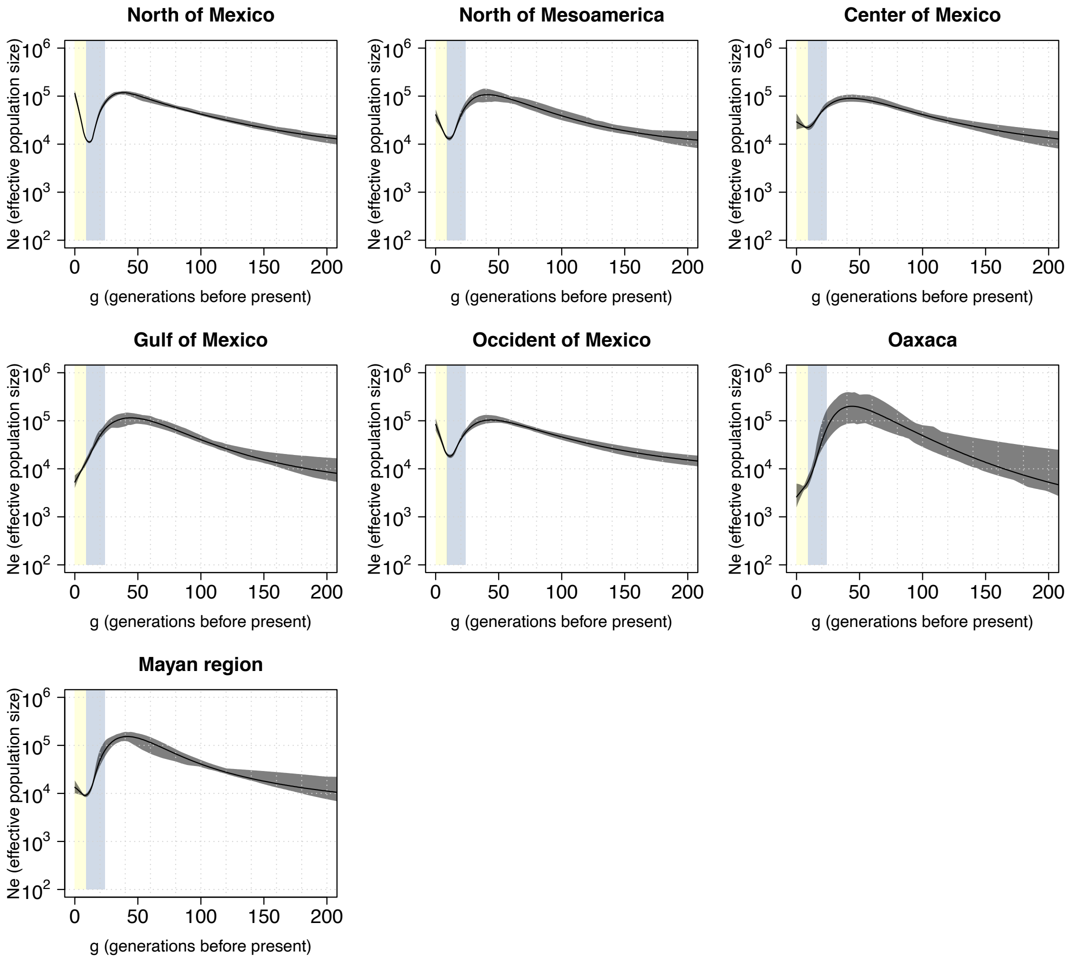

Fig. S27. Estimated effective population size for ancestries from Western Europe by Mesoamerican region in Mexico (20 years per generation). Effective population size changes were inferred for each Mesoamerican region using ancestry-specific IBD tracts. Each panel represents a different region of Mexico. The x-axis represents the number of generations before the present and the y-axis the effective population size. The colors in the panels represent colonial (purple) and postcolonial (light grey) periods (see timeline in the upper part of figure S25). The solid black lines show estimated ancestry-specific effective population sizes, and the gray regions show 95% bootstrap confidence intervals.

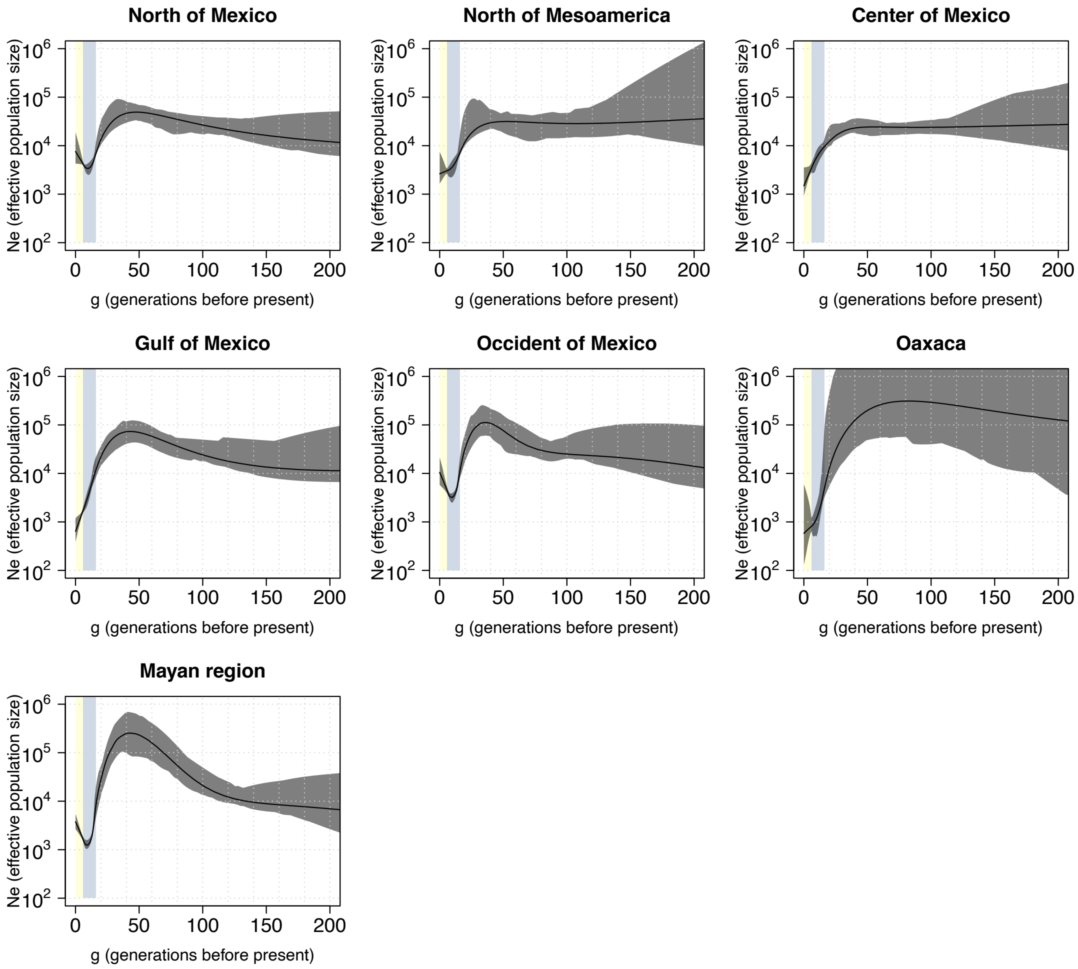

Fig. S28. Estimated effective population size for ancestries from West Africa by Mesoamerican region in Mexico (30 years per generation). Effective population size changes were inferred for each Mesoamerican region using ancestry-specific IBD tracts. Each panel represents a different region of Mexico. x-axis represents the number of generations before the present and the y-axis the effective population size. The colors in the panels represent colonial (purple) and postcolonial (light grey) periods (see timeline in the upper part of figure S25). The solid black lines show estimated ancestry-specific effective population sizes, and the gray regions show 95% bootstrap confidence intervals.

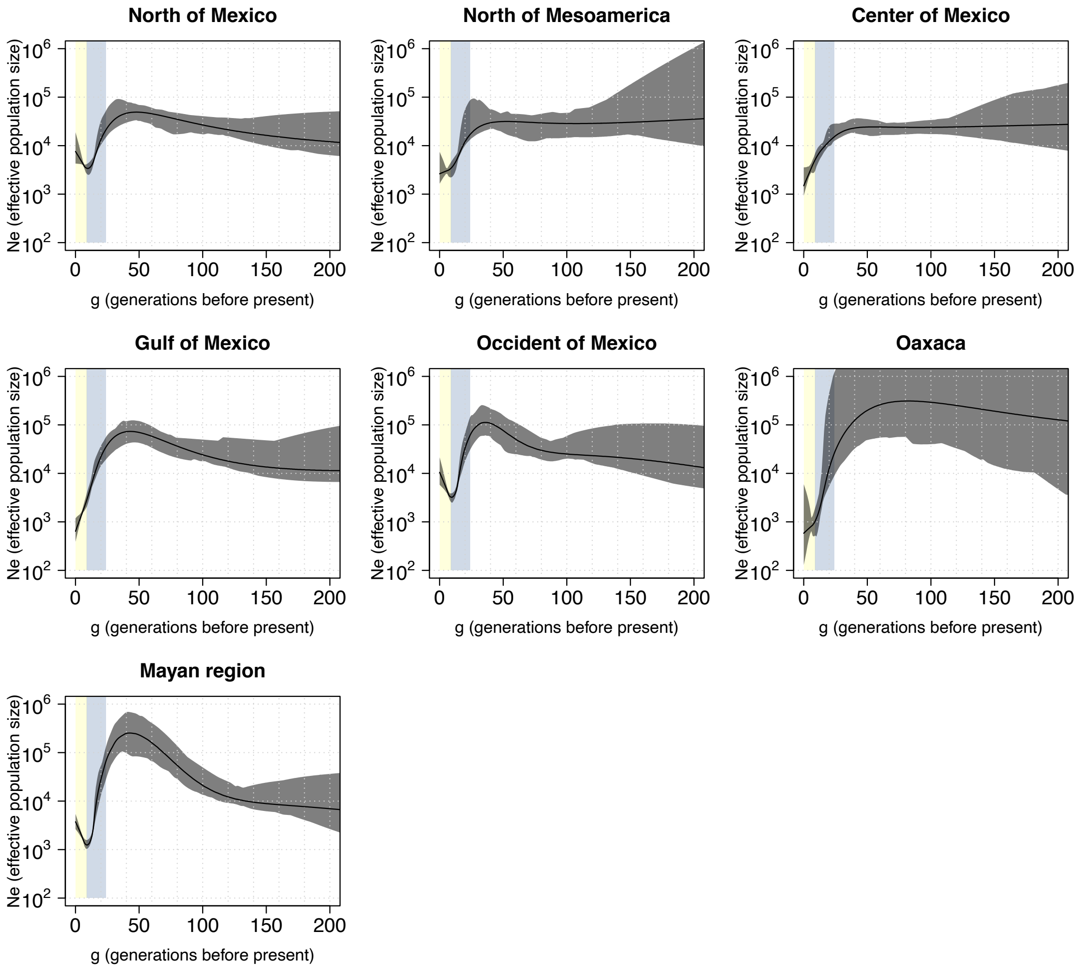

Fig. S29. Estimated effective population size for ancestries from West Africa by Mesoamerican region in Mexico (20 years per generation). Effective population size changes were inferred for each Mesoamerican region using ancestry-specific IBD tracts. Each panel represents a different region of Mexico. The x-axis represents the number of generations before the present and the y-axis the effective population size. The colors in the panels represent colonial (purple) and postcolonial (light grey) periods (see timeline in the upper part of figure S25). axis the effective population size. The colors in the panels represent colonial (purple) and postcolonial (light grey) periods (see timeline in the upper part of figure S25). The solid black lines show estimated ancestry-specific effective population sizes, and the gray regions show 95% bootstrap confidence intervals.

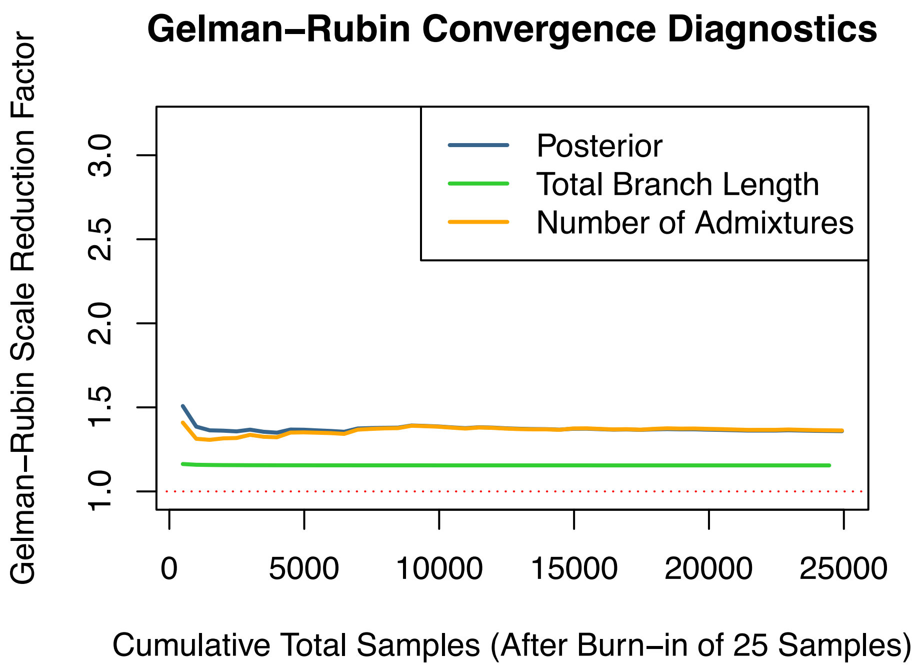

Fig. S30. Gelman-Rubin convergence plot for the AdmixtureBayes analysis.

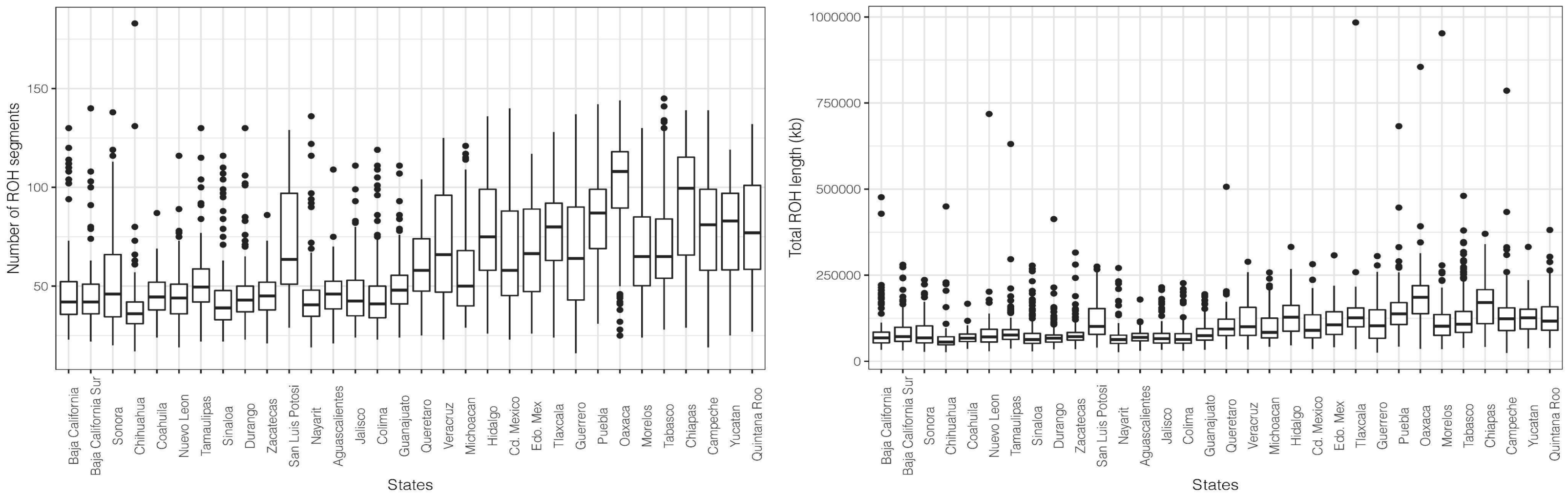

Fig. S31. NROH and SROH distribution by state. The x-axis shows the states of Mexico and the y-axis shows the number of Runs of Homozygosity (nROH) segments carried by an individual (left) or the total length of ROH (in kilobases) carried by an individual's genome (right). The boxplots show the distributions of these values by state. Boxplots show the median value and the quartiles. Whiskers extend the minimum and the maximum values. The dots represent outliers. n = 5833 biologically independent samples were used for both panels.

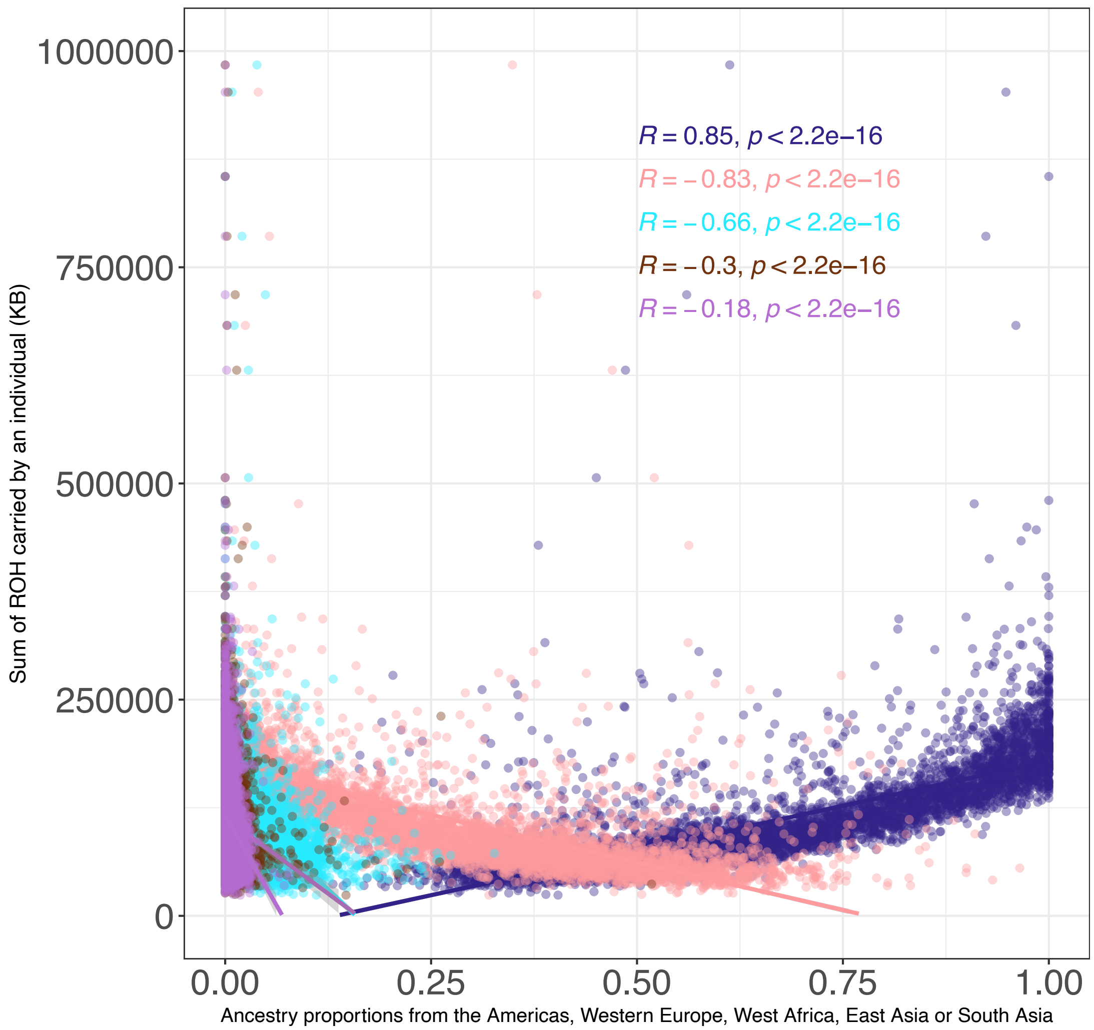

Fig. S32. SROH are correlated with ancestries in MXB. Ancestry proxies are inferred from *Admixture*[20]. Each panel shows the correlation between the total length of ROH segments (sROH) and the proportion of ancestries from the Americas, Western Europe, West Africa, East Asia or South Asia quantified using ADMIXTURE. Spearman correlation values are shown (R and two-sided P-values) for all ancestries.

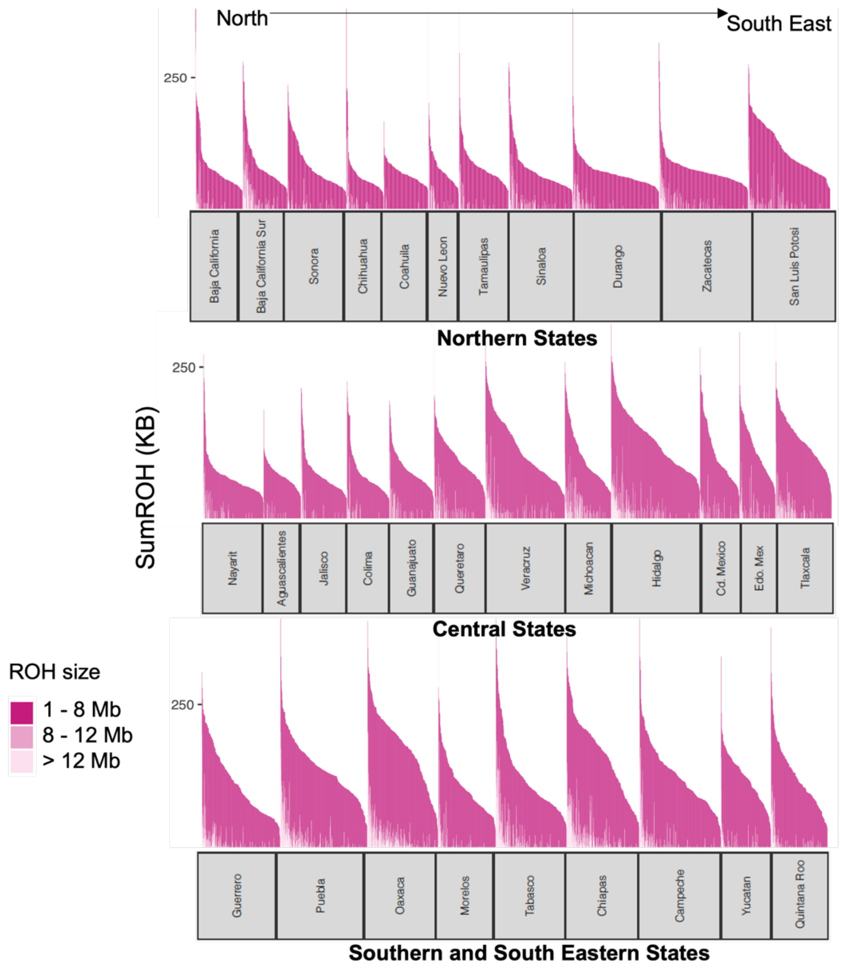

Figure S33. Distribution of ROH segments of different sizes for each Northern, Central, Southern and Southeastern state. The y-axis was truncated to aid visualization, truncating the first bar for some states. ROH was first broken into 3 bins (small, medium, and large). The sumROH per individual is shown here, highlighting the presence of small ROH especially moving south and southeastward in Mexico. The top panel shows northern states, the middle panel shows central states, and the bottom panel shows southern and southeastern states.

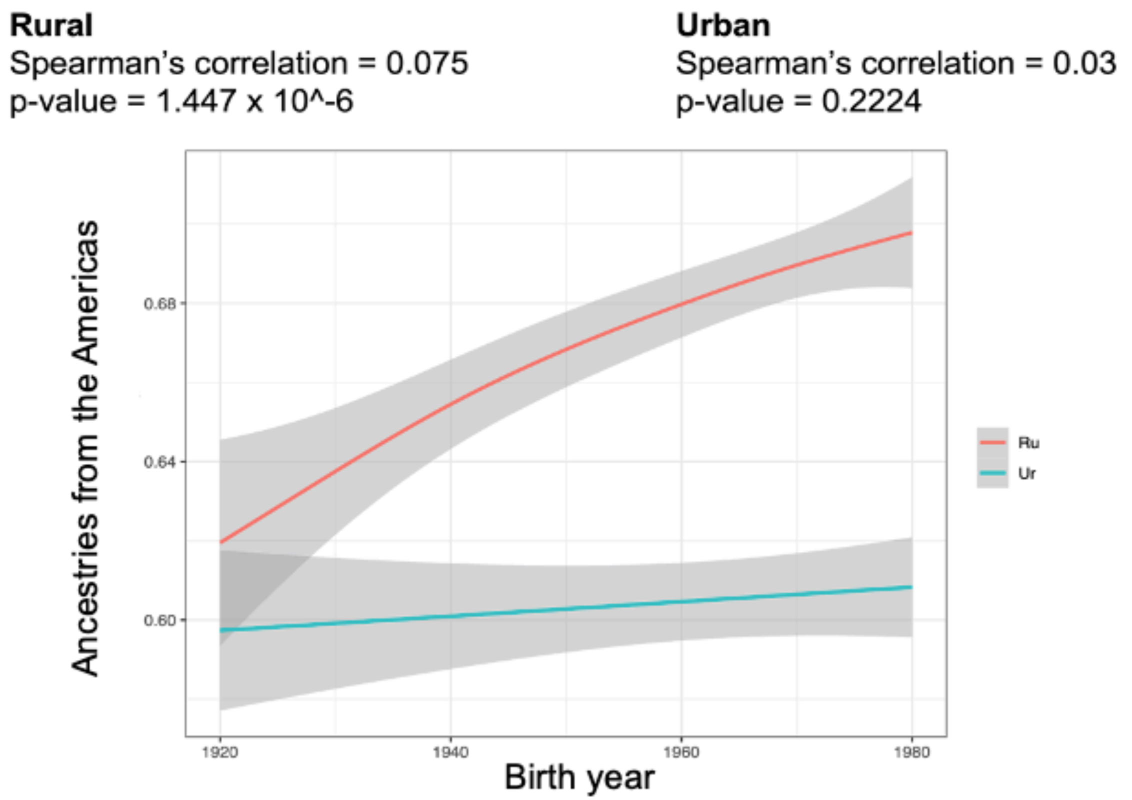

Fig. S34. Ancestries from the Americas as a function of birth year in rural and urban localities. Spearman correlation was estimated between birth year (x-axis) and the inferred proportion of ancestries from the Americas computed using Admixture (y-axis) for individuals that live in rural (orange) and urban areas (blue). A significant positive correlation between birth year and proportion of ancestries from the Americas is seen in individuals that live in rural areas. Smoothed conditional mean line are shown using the loess smoothing method. Error bands represent 95% confidence intervals. Spearman correlation values are shown (R and two-sided P-values).

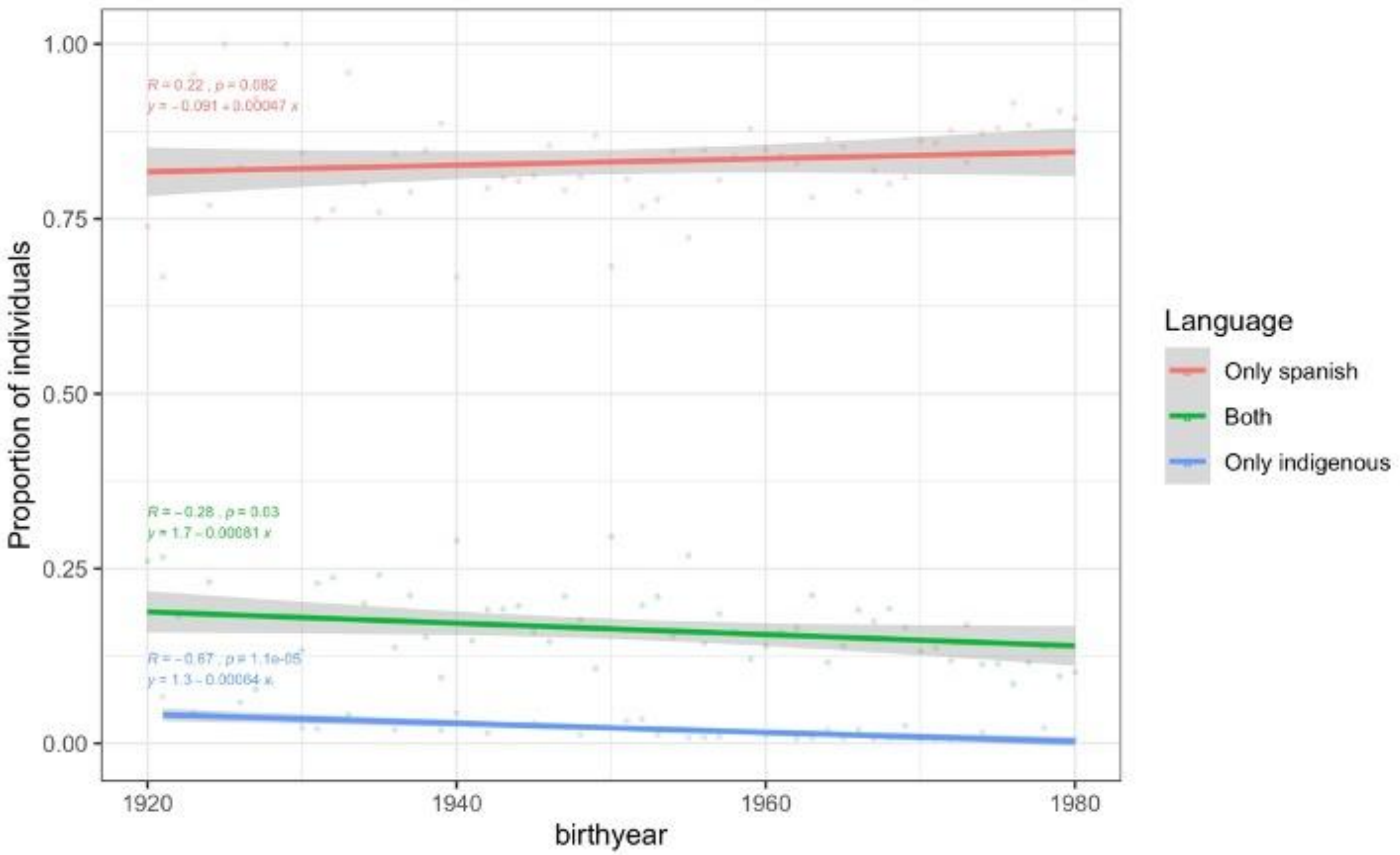

Fig. S35. Proportion of individuals that speak Spanish, an Indigenous language or both, as a function of birth year. Correlation between birthyear (x-axis) and the proportion of individuals (y-axis) that speak only Spanish (orange), both (green) and only an Indigenous language (blue). A negative correlation is seen between birth year and the proportion of individuals that speak both Spanish and an Indigenous language, or only an Indigenous language. Smoothed conditional mean lines are shown using a linear model. Error bands represent 95% confidence intervals. Spearman correlation values (R and two-sided P-values), and the fitted values for a linear model are shown for all three language categories.

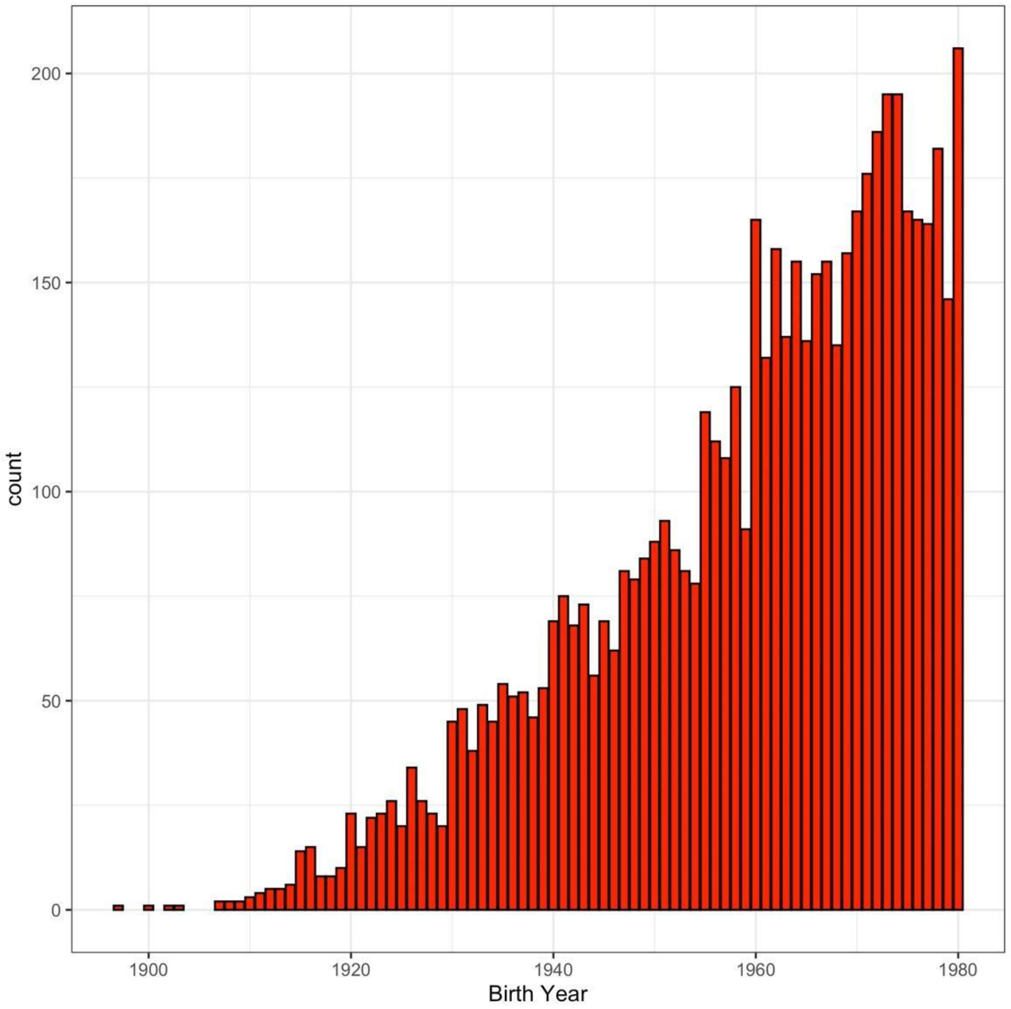

Fig. S36. Sample counts by birth year in the MXB. Histogram of the MXB samples born by year. A sampling bias towards younger individuals is observed.

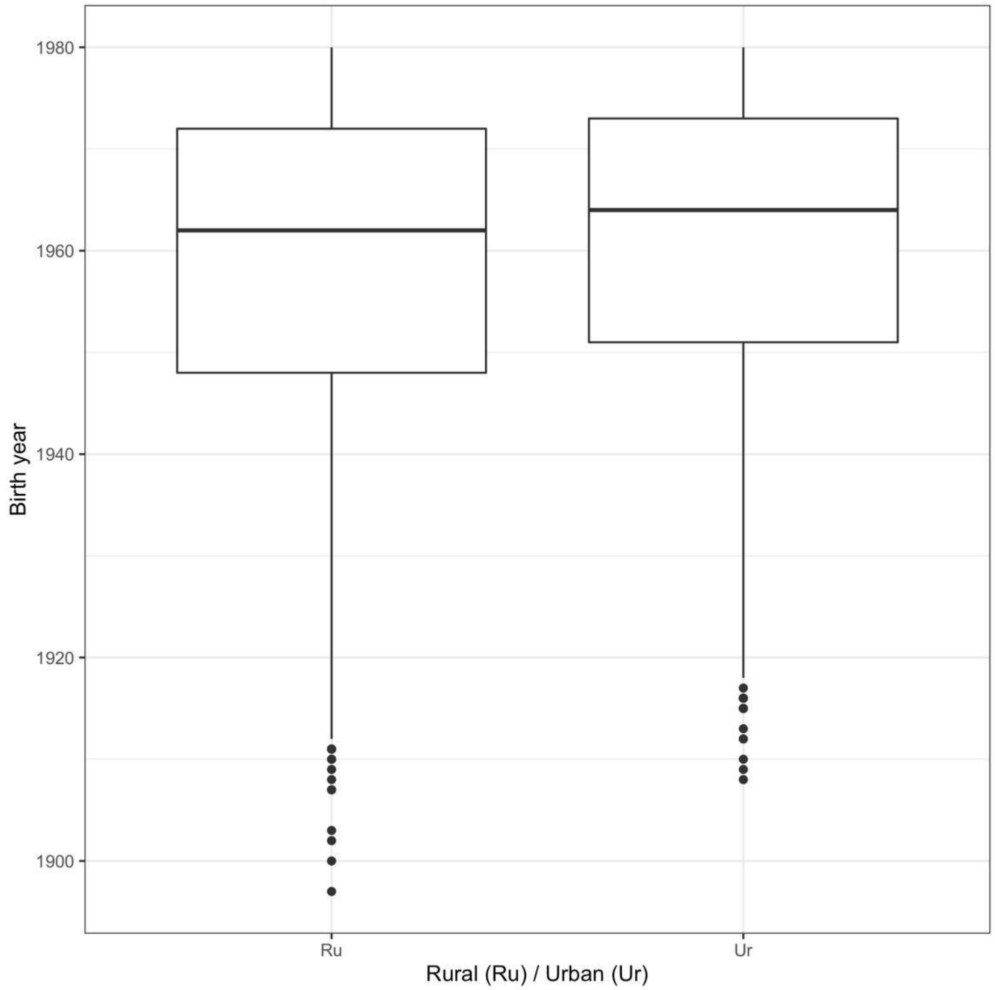

Fig. S37. Birth year distribution by rural or urban areas. Individuals in rural areas are significantly older than individuals in urban areas (Wilcoxon test W = 3221193, p-value = 6.518×10^{-7}). The boxplots show the median value and the quartiles. Whiskers extend the minimum and the maximum values. The dots represent outliers. n = 5929 biologically independent samples were used for the analysis.

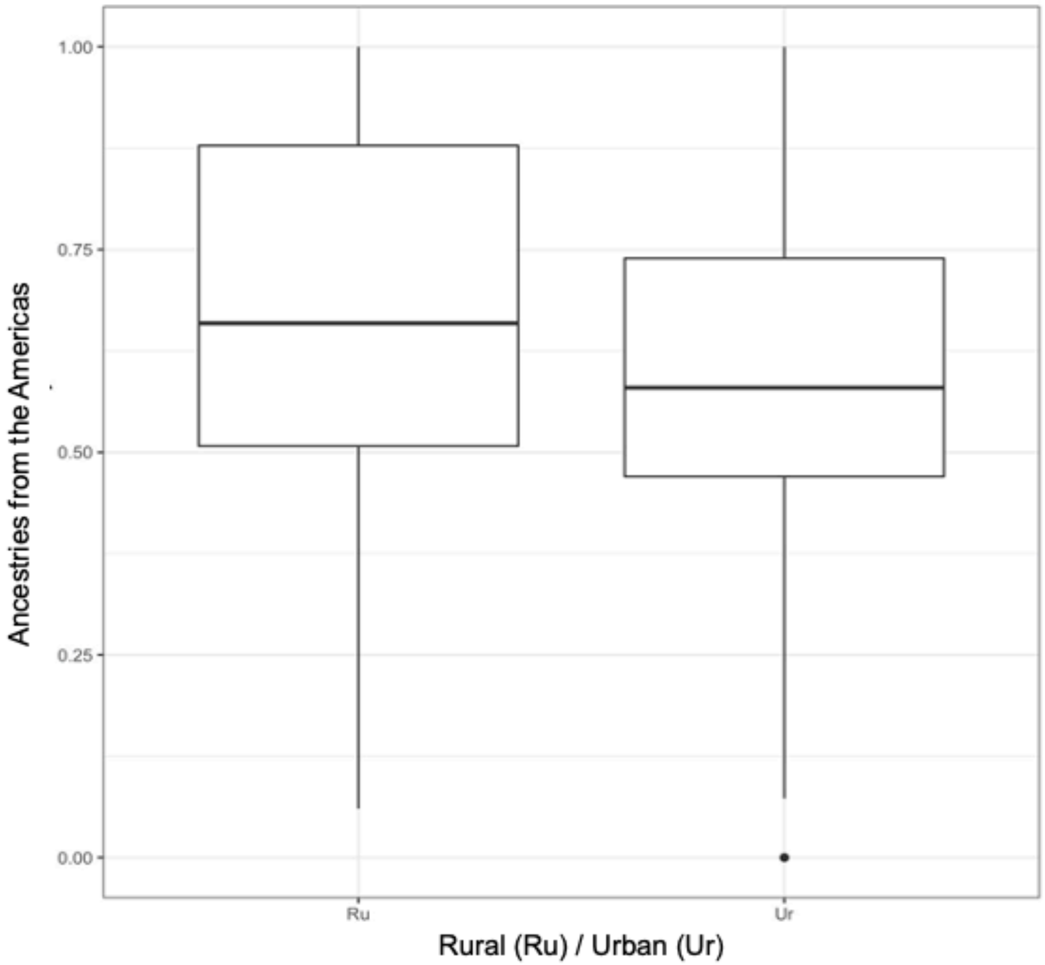

Fig. S38. Distribution of ancestries from the Americas by rural and urban areas. We observe significantly higher proportion of ancestries from the Americas (inferred using Admixture) in rural areas compared to in urban areas (Wilcoxon test W = 3972985, p-value < 2.2×10^{-16}). The boxplots show the median value and the quartiles. Whiskers extend the minimum and the maximum values. The dots represent outliers. n = 5753 biologically independent samples were used for the analysis.

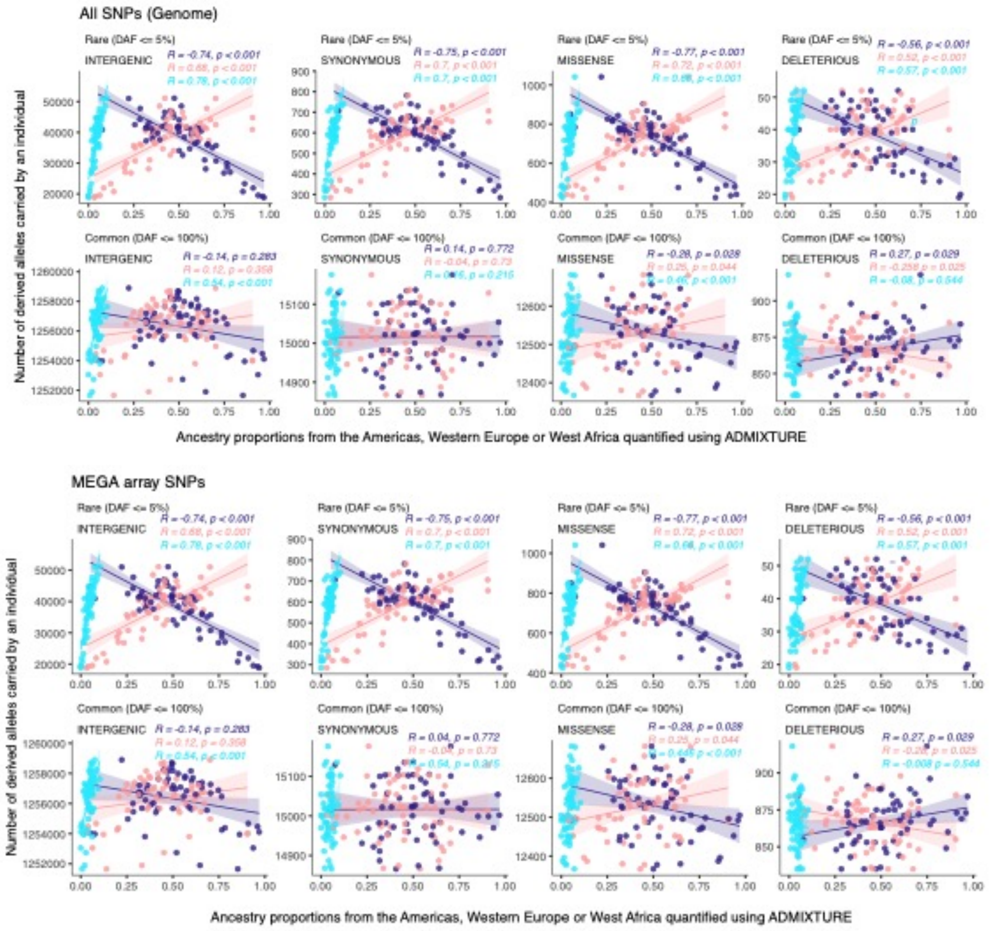

Fig. S39. Mutation burden in 1000G MXL individuals as a function of their ancestries from the Americas, Western Europe, and West Africa. Colors show ancestries from the Americas (red), Western Europe (green) or West Africa (blue). The top panel (first and second rows) shows mutation burden computed on All SNPs (Genome) while the bottom panel (third and fourth row) shows mutation burden computed on only the subset of MEGA array SNPs. The first and third row show the analysis for rare variants (DAF <= 5%) and the second and fourth row show the analysis for common SNPs (DAF <= 100%). Smoothed conditional mean lines are shown using a linear model. Error bands represent 95% confidence intervals. Spearman correlation values are shown (R and two-sided P-values) for all ancestries. A significant correlation between mutation burden and ancestry is seen for rare mutation burden in both the MEGA array SNPs and All SNPs (Genome) due to differential bottlenecking histories reflected in different ancestry proxies. In contrast, no clear or strong significant correlations between mutation burden and ancestry are seen for total mutation burden. Thus, rare mutation burden shows a robust correlation with ancestry proxies, while total mutation burden does not. Each column shows the analysis for a different set of variants, intergenic, synonymous, missense and deleterious (see methods).

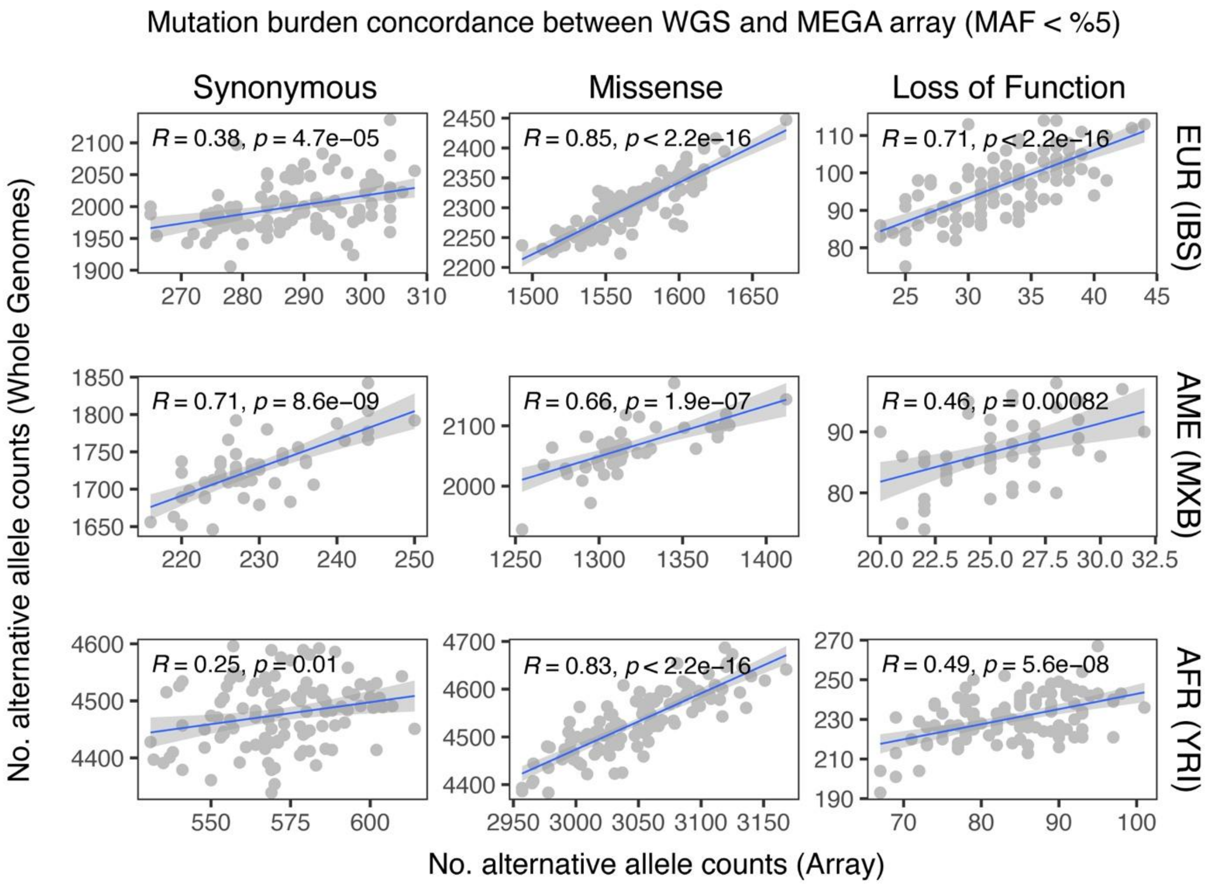

Fig. S40. Rare mutation burden computed on WGS vs. array SNPs. This analysis was done using 50 genomes sequenced as part of the MXB (second row), as well as using IBS (first row) and YRI (third row) cohorts from the 1000 Genomes Project. Rare mutation burden computed using all SNPs in WGS or only SNPs in the array data were correlated. Each column shows the analysis for a different set of variants, synonymous, missense and loss of function. A smoothed conditional mean line is shown using a linear model. Error bands represent 95% confidence intervals. Spearman correlation values are shown (R and two-sided P-values). There is a significant correlation in all types of variants (synonymous, missense and loss of function) in the three analyzed cohorts.

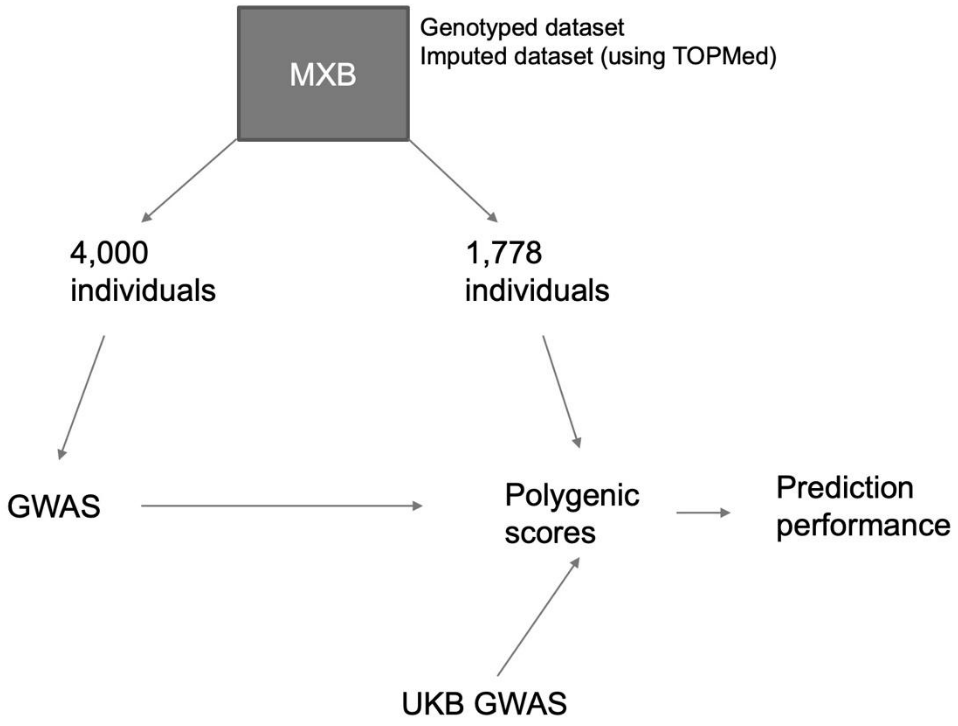

Fig. S41 Schematic for testing prediction performance of polygenic scores computed in the MXB using MXB or UKB GWAS. GWAS was performed in only 4,000 randomly selected individuals and polygenic scores were computed for the rest of the individuals in the MXB. UKB GWAS pan-ancestry summary statistics were also used to compute polygenic scores in the same MXB individuals. The impact of using different GWAS was assessed (Tables S8-S10).

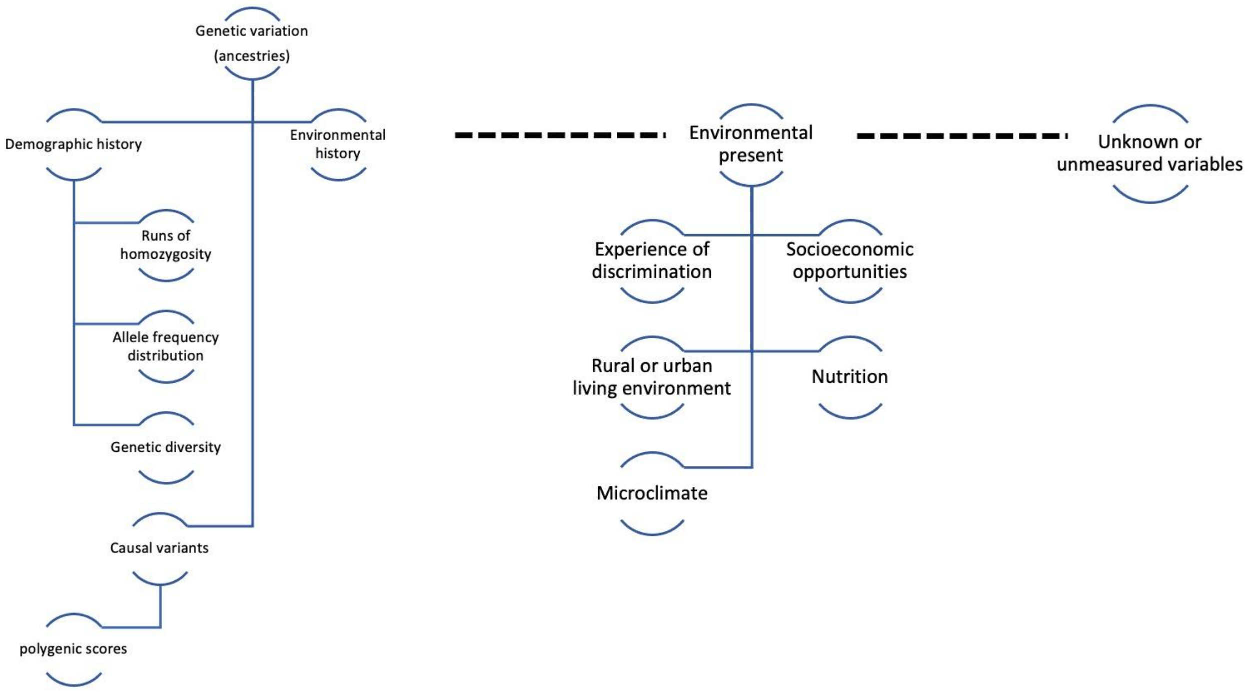

Fig. S42. A conceptual framework used in this study for the role of genetics and environment in creating genetic variation and variation in complex traits and disease risk. The top layer represents a higher-order characterization of factors affecting phenotypic variation. The lower layers reflect the fine-grained components of these factors. Dashed lines represent potential correlations between factors. Genetic variation (ancestries) reflects variable demographic histories (reflected in patterns of ROH, allele frequencies and genetic diversity), environmental histories and causal variant distributions (proxied using polygenic scores). Genetic variation can further be correlated with the environmental present. The interrelation between genetics and environment is very complex and there are likely variables unknown or unmeasured that undoubtedly participate in the framework.

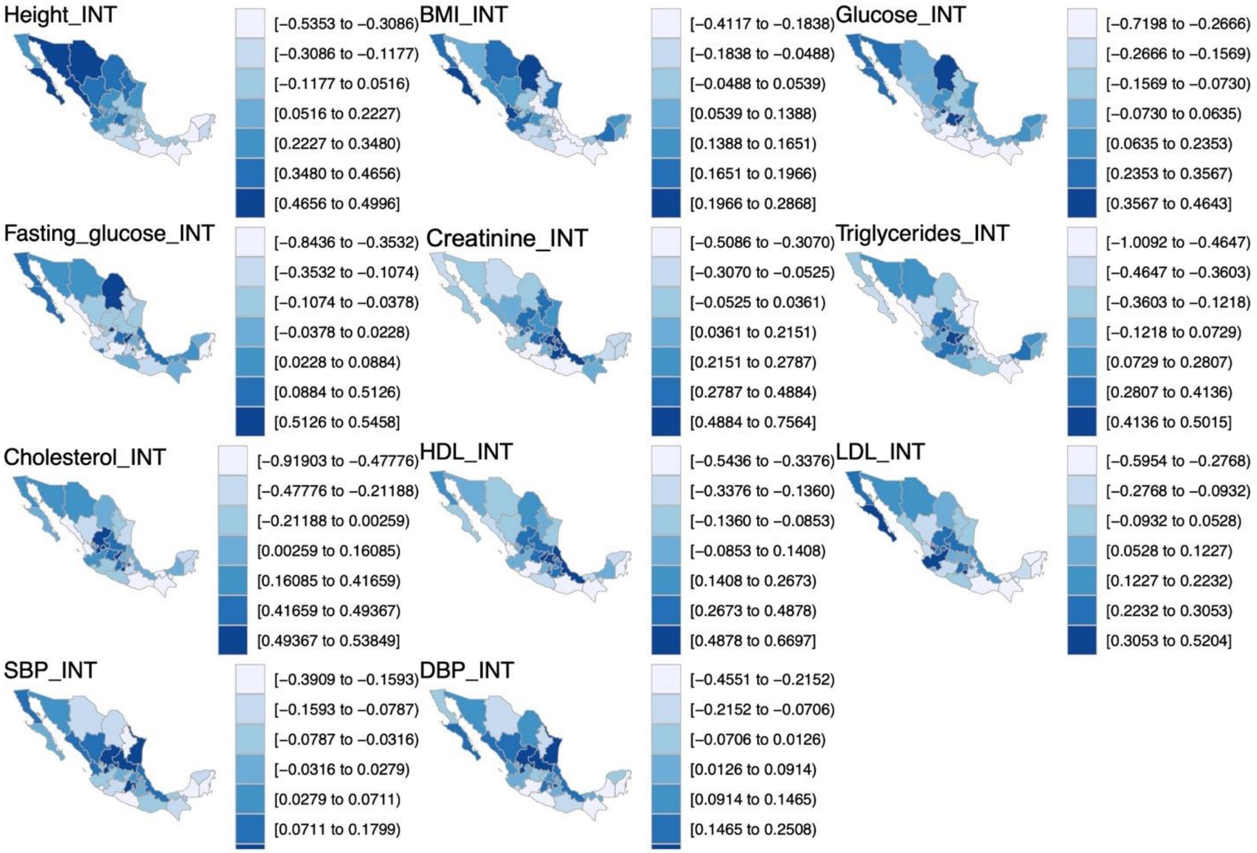

Fig. S43. Average trait values per state visualized on the map of Mexico. Quantitative traits were inverse normalized and average values for transformed traits per state are shown here. See methods (The Mexican Biobank Project: phenotype, lifestyle and environmental data) for further details on the quantitative traits analyzed. Each panel shows the map for a different quantitative trait.

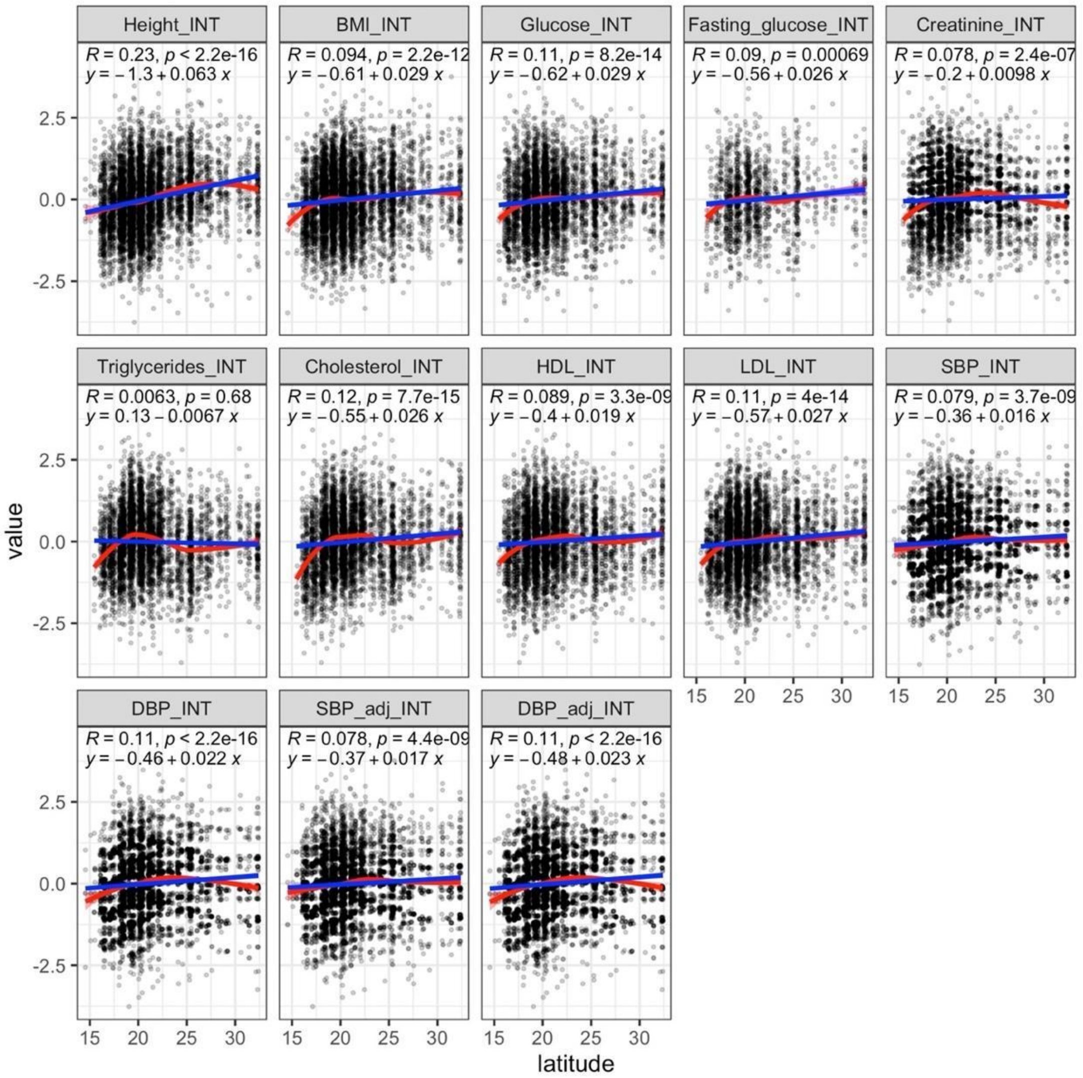

Fig. S44. Complex trait variation by latitude. To aid with visualization, a smoothed conditional mean line is shown using the loess smoothing method (red) or a linear model (blue). Each panel shows the results for the indicated trait. The x-axis shows the latitude value and the y-axis shows the value of the particular trait. Spearman correlation values (R and two-sided P-values), and the fitted values for a linear model are shown for all traits.

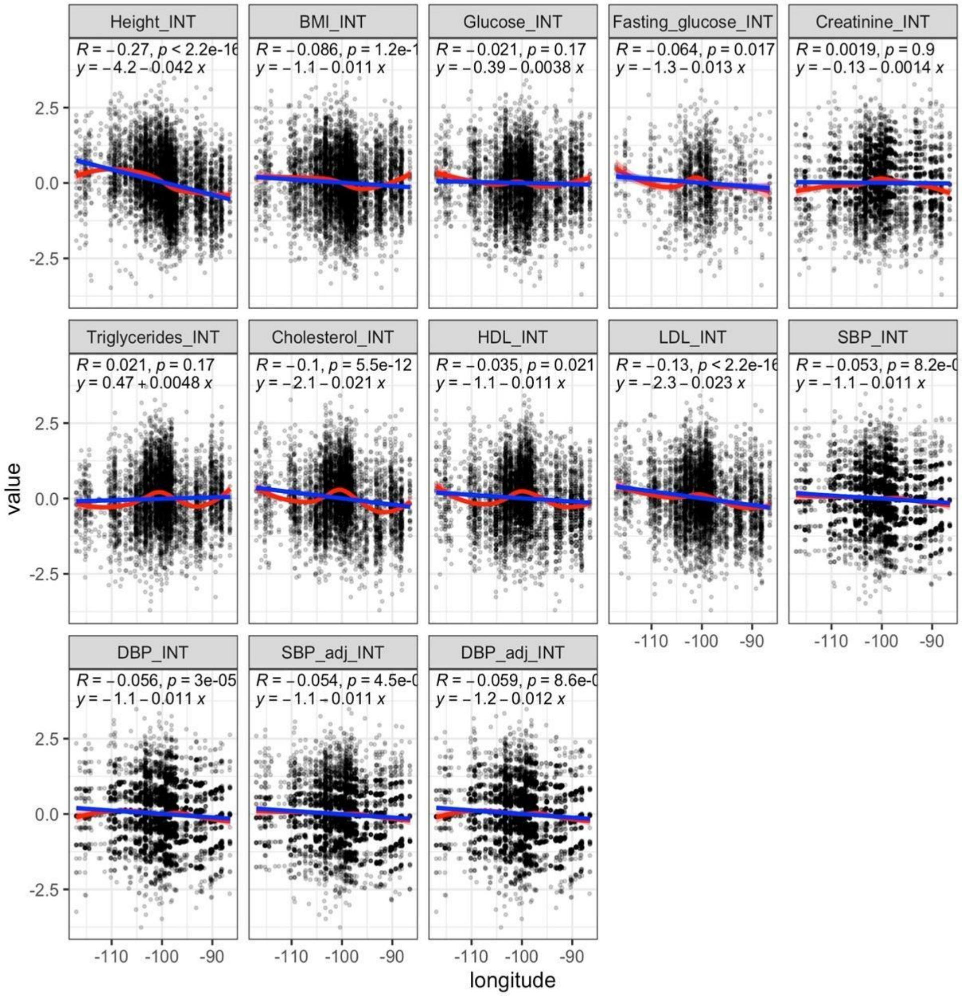

Fig. S45. Complex trait variation by longitude. To aid with visualization, a smoothed conditional mean line is shown using the loess smoothing method (red) or a linear model (blue). Each panel shows the results for the indicated trait. The x-axis shows the longitude value and the y-axis shows the value of the particular trait. Spearman correlation values (R and two-sided P-values), and the fitted values for a linear model are shown for all traits.

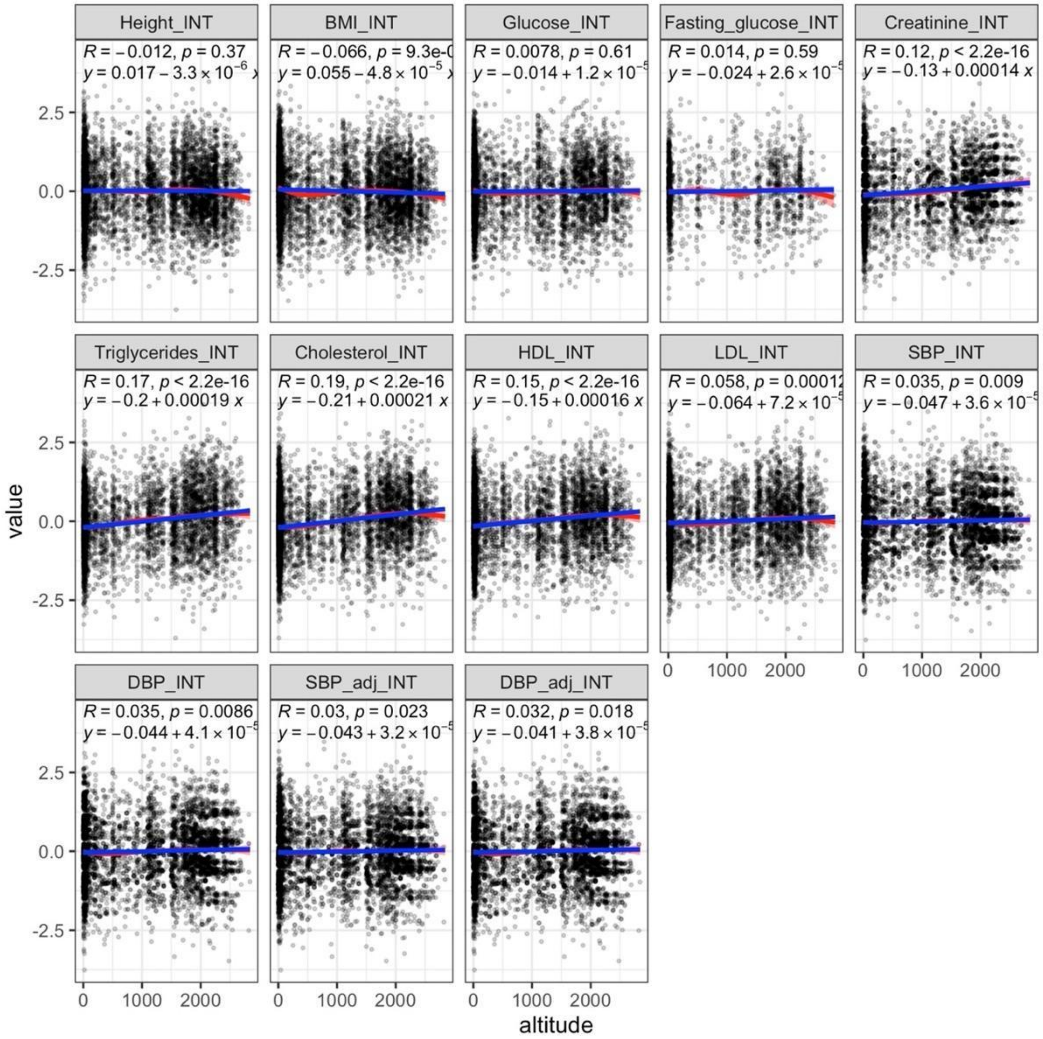

Fig. S46. Complex trait variation by altitude. To aid with visualization, a smoothed conditional mean line is shown using the loess smoothing method (red) or a linear model (blue). Each panel shows the results for the indicated trait. The x-axis shows the altitude value and the y-axis shows the value of the particular trait. Spearman correlation values (R and two-sided P-values), and the fitted values for a linear model are shown for all traits.

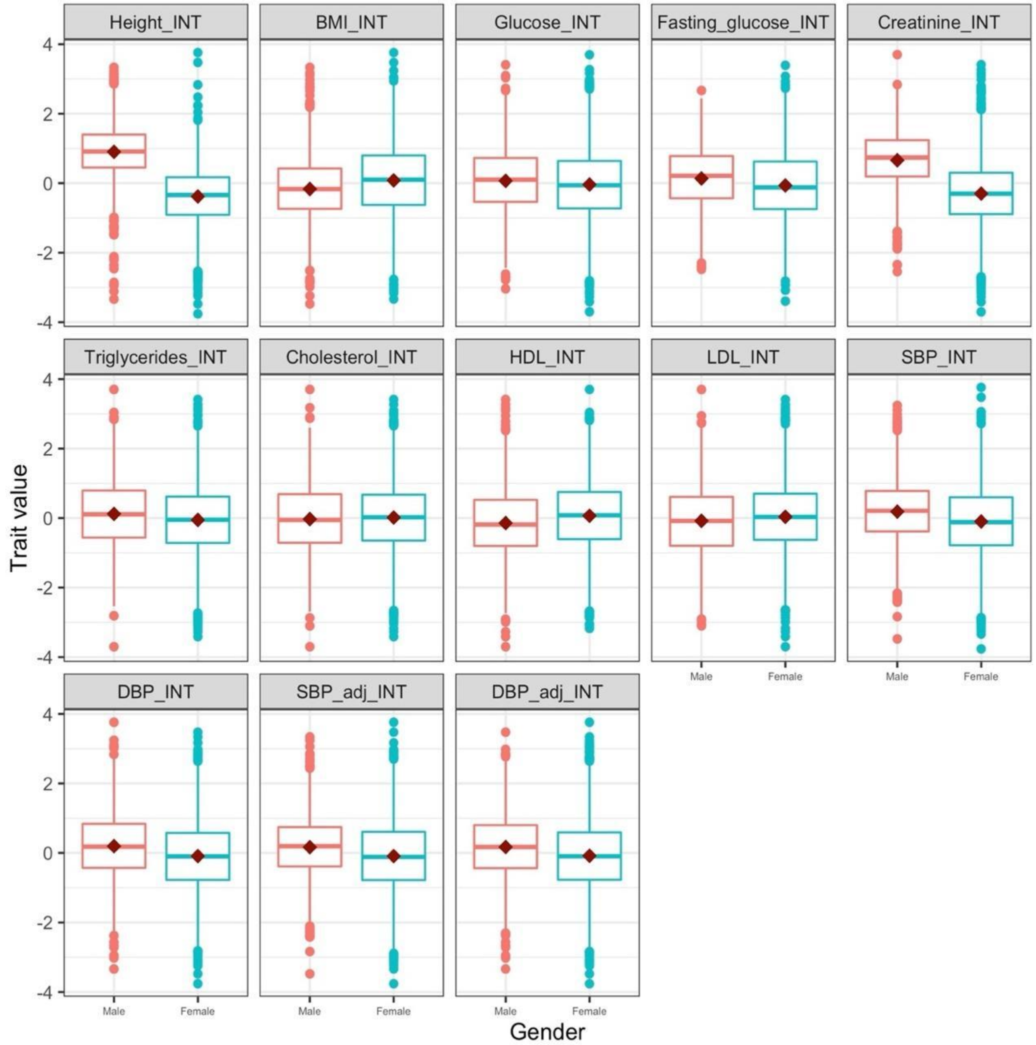

Fig. S47. Complex trait variation by sex. Box plots of trait values are shown for males (red) and females (blue). Each subplot shows the values of the specified trait. Box plots show median, mean and quartiles. Each panel shows the results for the indicated trait. Whiskers extend the minimum and the maximum values. The dots represent outliers. n = 5770, 5747, 4516, 1427, 4582, 4583, 4584, 4584, 4553, 5836, 5836, 5836 and 5836 biologically independent samples were used for each trait from Height (Height_INT) to adjusted Diastolic blood pressure (DBP_adj_INT) respectively (top left to bottom right).

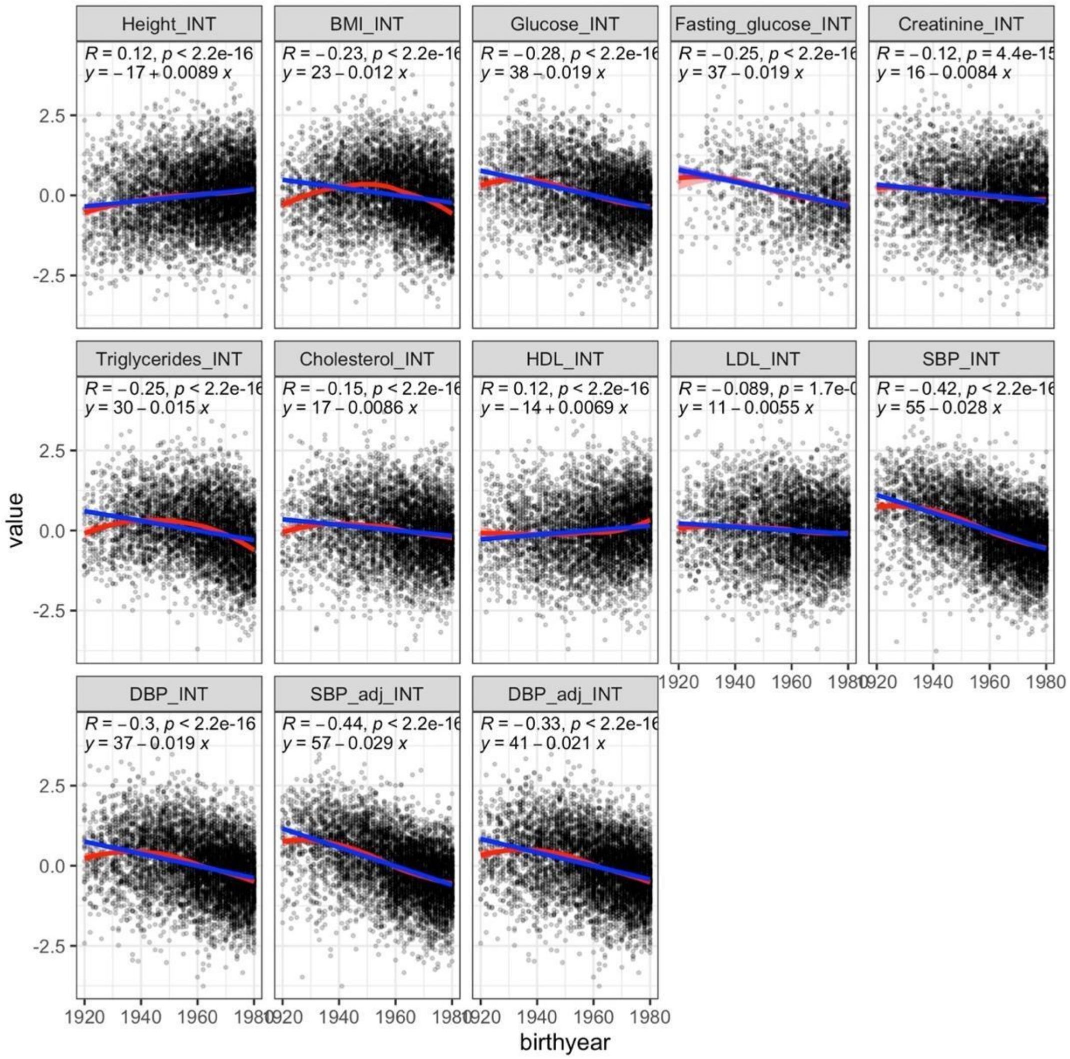

Fig. S48. Complex trait variation by birth year. To aid with visualization, a smoothed conditional mean line is shown using the loess smoothing method (red) or a linear model (blue). Each panel shows the results for the indicated trait. The x-axis shows the birth year and the y-axis shows the value of the particular trait. Spearman correlation values (R and two-sided P-values), and the fitted values for a linear model are shown for all traits.

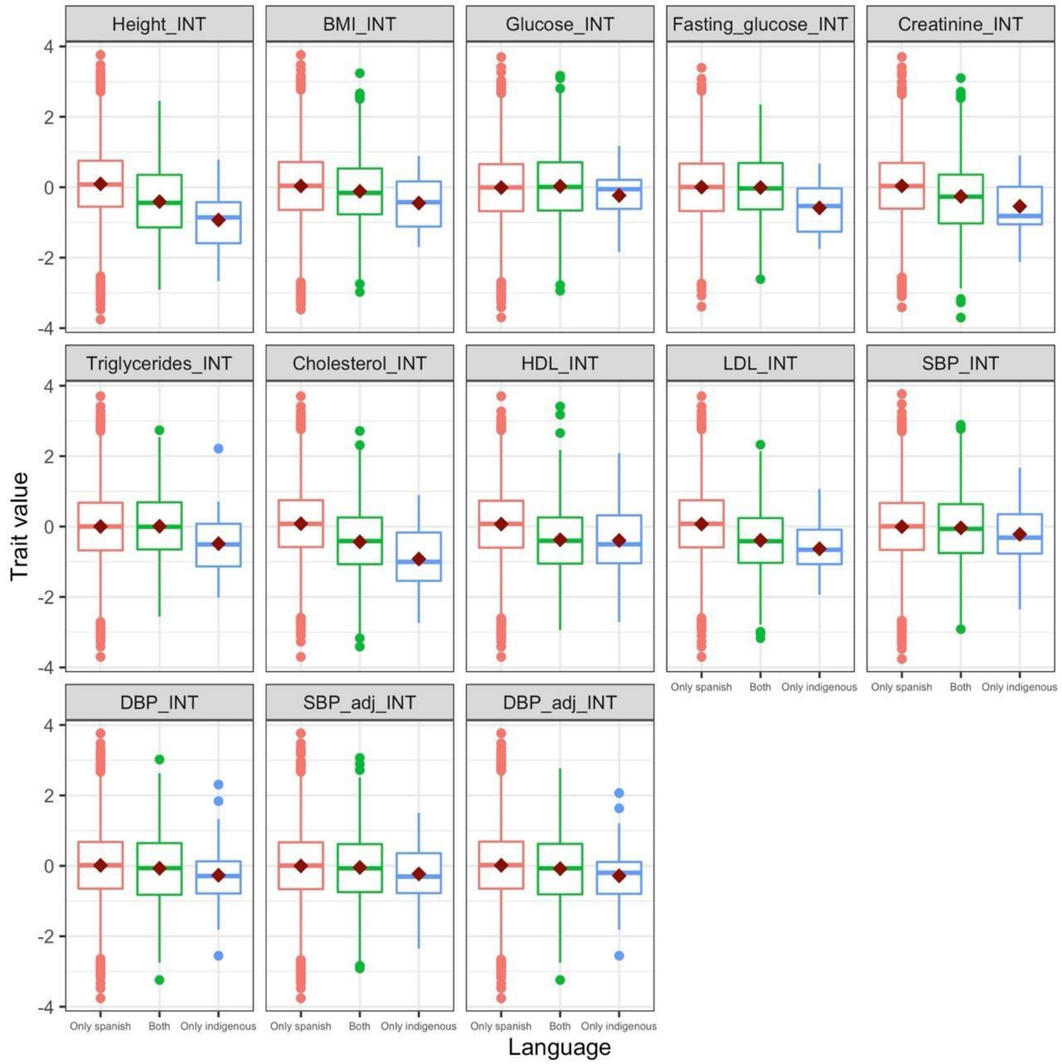

Fig. S49. Complex trait variation by Indigenous heritage. Box plots of trait values are shown for individuals that speak only Spanish (red), that speak both Spanish and an Indigenous language (green), and that speak only an Indigenous language (blue). Box plots show median, mean and quartiles. Each panel shows the results for the indicated trait. Whiskers extend the minimum and the maximum values. The dots represent outliers. n = 5770, 5747, 4516, 1427, 4582, 4583, 4584, 4584, 4553, 5836, 5836, 5836 and 5836 biologically independent samples were used for each trait from Height (Height_INT) to adjusted Diastolic blood pressure (DBP_adj_INT) respectively (top left to bottom right).

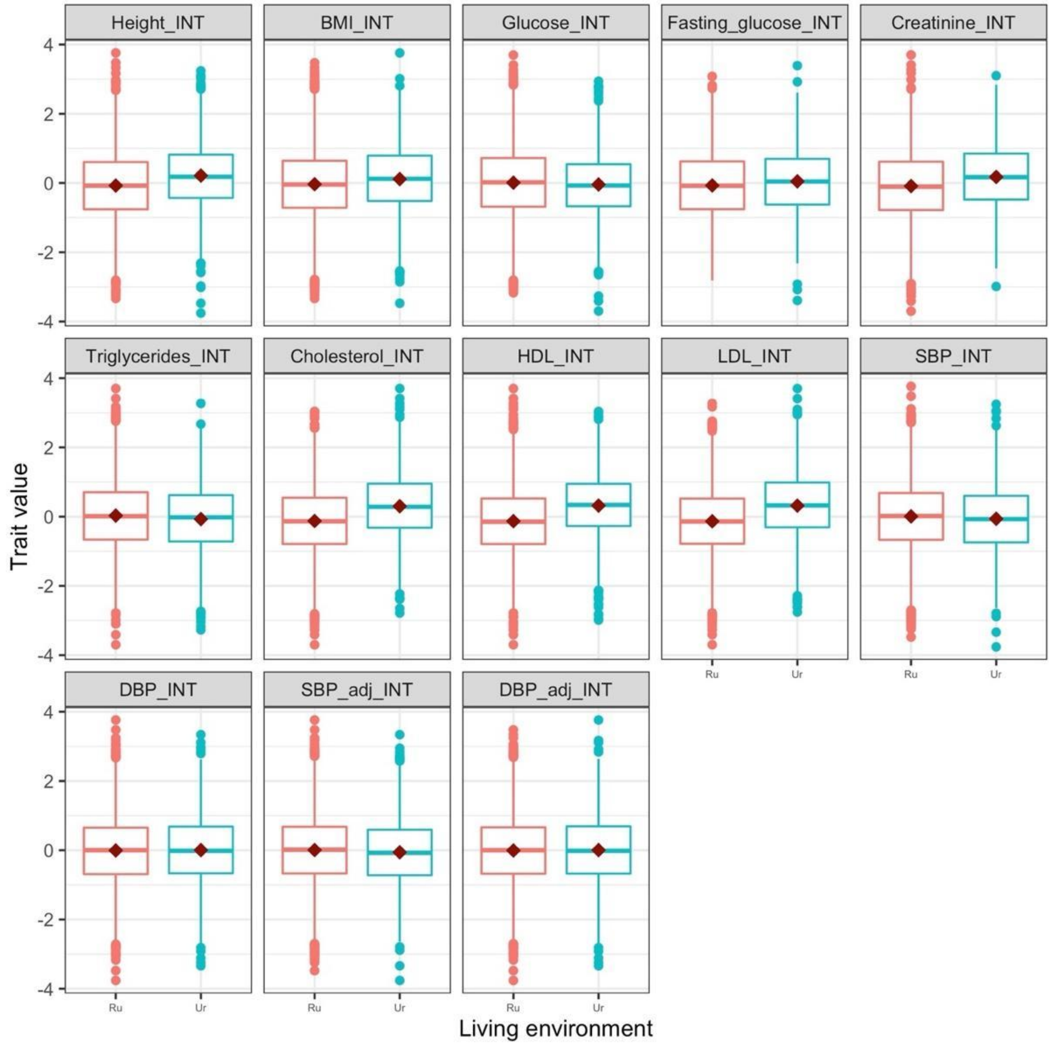

Fig. S50. Complex trait variation by rural or urban living environment. Box plots of trait values are shown for individuals that live in a rural area (red) and an urban area (blue). Box plots show median, mean and quartiles. Each panel shows the results for the indicated trait. Whiskers extend the minimum and the maximum values. The dots represent outliers. n = 5770, 5747, 4516, 1427, 4582, 4583, 4584, 4584, 4553, 5836, 5836, 5836 and 5836 biologically independent samples were used for each trait from Height (Height_INT) to adjusted Diastolic blood pressure (DBP_adj_INT) respectively (top left to bottom right).

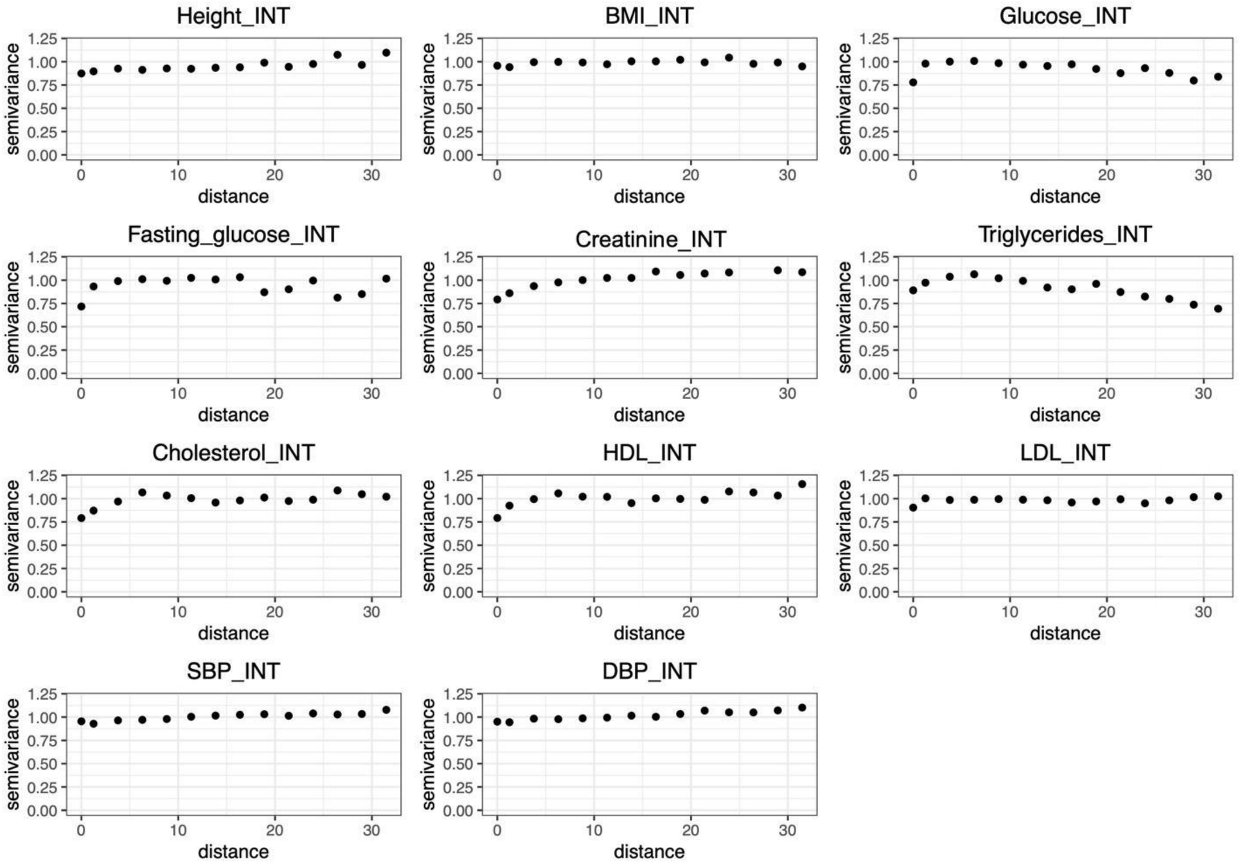

Fig. S51. Variograms of complex traits. Variogram was computed using a classical (method of moments) estimator, and computing distance using longitude and latitude. Each panel shows the results for the indicated trait.

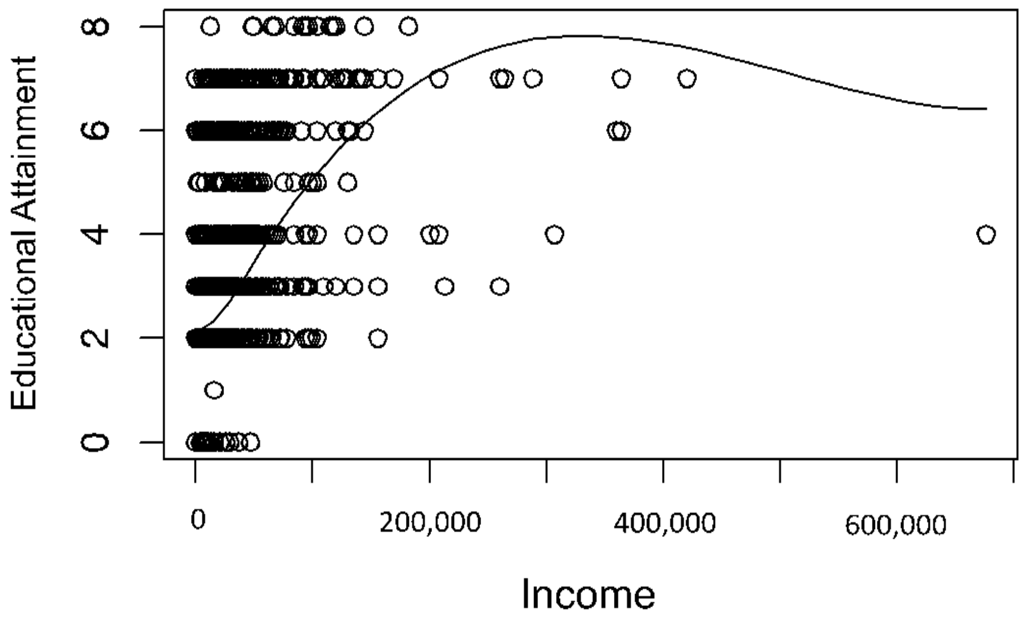

Fig. S52. Correlation of educational attainment and income levels in the MXB. Education attainment is correlated with income levels (in pesos). An epidemiological variable in its own right, it can also serve as a proxy for income levels in the complex trait modeling, as this metric is available for the majority of MXB individuals compared to income levels (only available for a fraction).

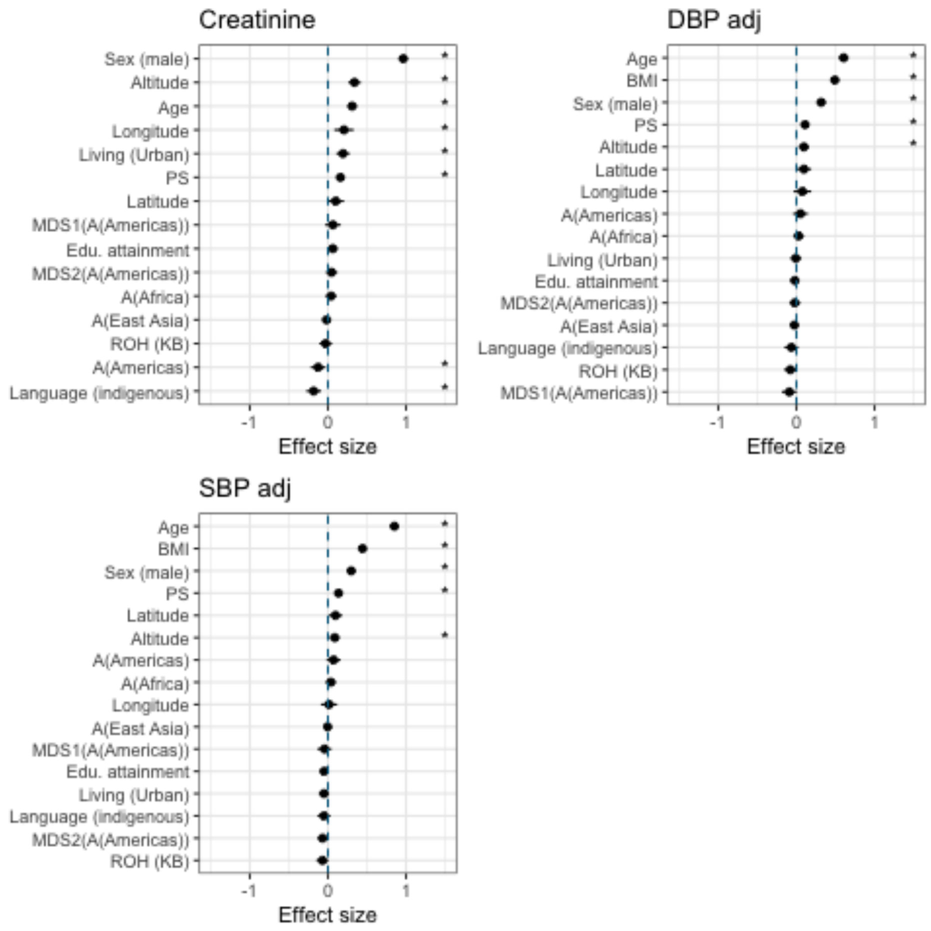

Fig. S53. An analysis of complex trait variation in Creatinine and blood pressure in the MXB. The plot shows effect size estimates and confidence intervals from a mixed model analysis. All quantitative predictors are centered and scaled by 2 standard deviations. Asterisks show significance at FDR < 0.05 across traits and predictors analyzed. Panels are shown for creatinine, diastolic blood pressure (DBP adj) and systolic blood pressure (SBP adj), both adjusted for medication status (see methods). The plot shows effect size estimates and confidence intervals (1.96*SEM) from a mixed model analysis. n = 3714, 4605 and 4605 biologically independent samples were used for the analysis for Creatinine, adjusted Diastolic blood pressure (DBP adj) and adjusted Systolic blood pressure (SBP adj) respectively.

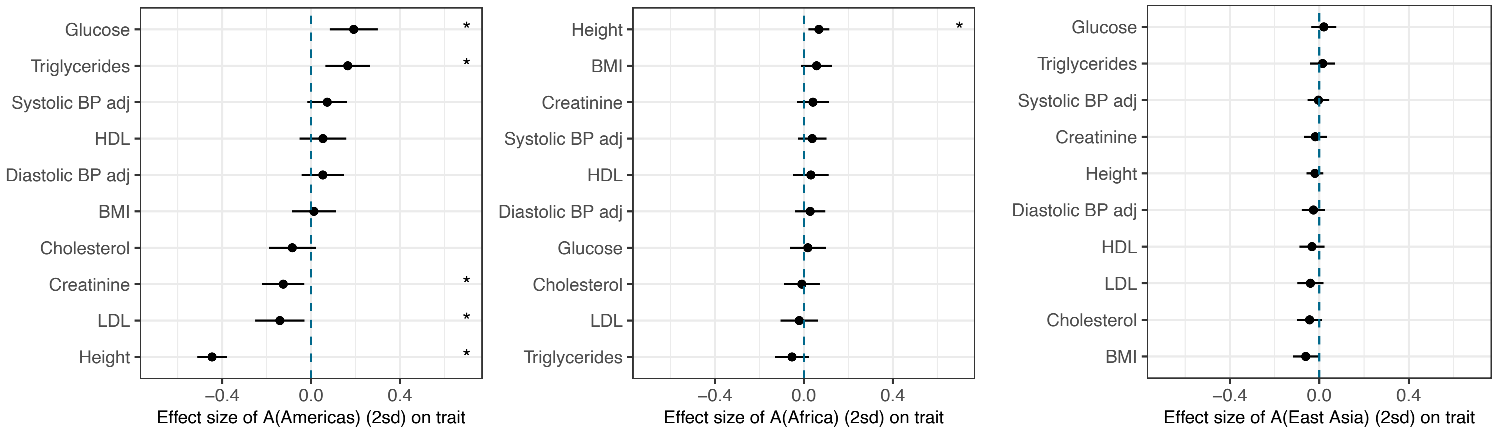

Fig. S54. Mixed model results for ancestries from different global regions in the MXB. The plot shows effect size estimates and confidence intervals from a mixed model analysis for A(Americas) (left panel), A(Africa) (middle panel) and A(East Asia) (right panel). These reflect ancestry proxies quantified from an Admixture analysis (at K=5) and these predictors are centered and scaled by 2 standard deviations. Asterisks show significance at FDR < 0.05 across traits and predictors analyzed. The plot shows effect size estimates and confidence intervals (1.96*SEM) from a mixed model analysis. n = 4625, 4607, 3664, 3613, 3714, 4605, 4605, 3665, 3665 and 3641 biologically independent samples were used for the analysis for Height, BMI, Triglycerides, Glucose, Creatinine, adjusted Diastolic blood pressure (DBP adj), adjusted Systolic Blood pressure (SBP adj), total Cholesterol, HDL and LDL cholesterol levels respectively.

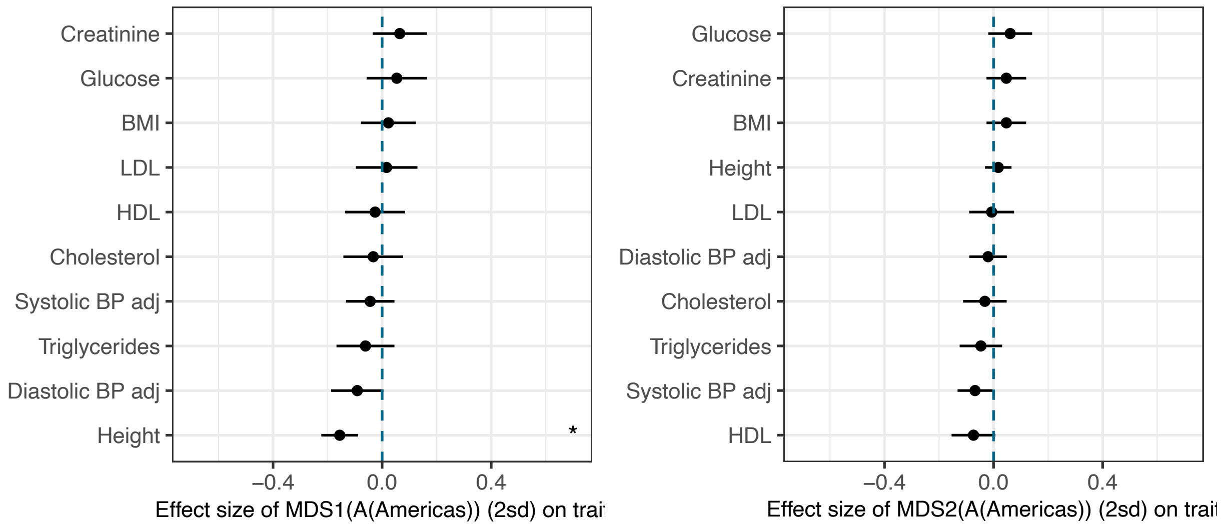

Fig. S55. Mixed model results for two MDS axes reflecting genetic variation within the Americas. The plot shows effect size estimates and confidence intervals from a mixed model analysis for MDS axis 1 (left panel) and MDS axis 2 (right panel). MDS axes are centered and scaled by 2 standard deviations. Asterisks show significance at FDR < 0.05 across traits and predictors analyzed. The plot shows effect size estimates and confidence intervals (1.96*SEM) from a mixed model analysis. n = 4625, 4607, 3664, 3613, 3714, 4605, 4605, 3665, 3665 and 3641 biologically independent samples were used for the analysis for Height, BMI, Triglycerides, Glucose, Creatinine, adjusted Diastolic blood pressure (DBP adj), adjusted Systolic Blood pressure (SBP adj), total Cholesterol, HDL and LDL cholesterol levels respectively.

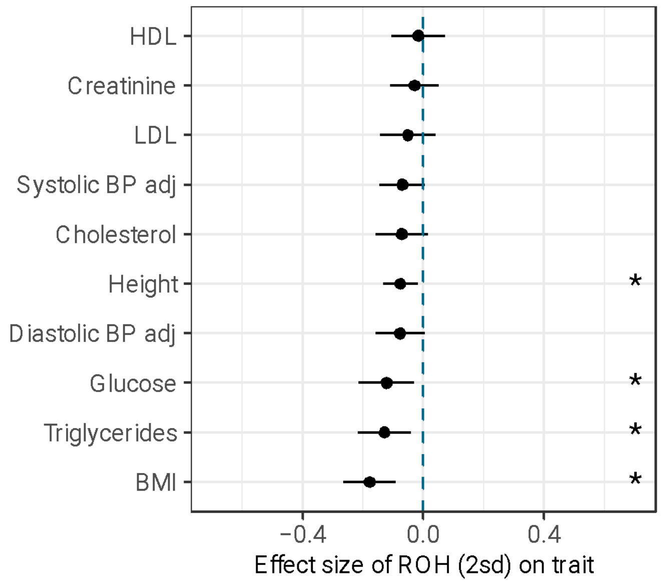

Fig. S56. Mixed model results for sROH carried by an individual. The plot shows effect size estimates and confidence intervals from a mixed model analysis. ROH predictor is centered and scaled by 2 standard deviations. Asterisks show significance at FDR < 0.05 across traits and predictors analyzed. The plot shows effect size estimates and confidence intervals (1.96*SEM) from a mixed model analysis. n = 4625, 4607, 3664, 3613, 3714, 4605, 4605, 3665, 3665 and 3641 biologically independent samples were used for the analysis for Height, BMI, Triglycerides, Glucose, Creatinine, adjusted Diastolic blood pressure (DBP adj), adjusted Systolic Blood pressure (SBP adj), total Cholesterol, HDL and LDL cholesterol levels respectively.

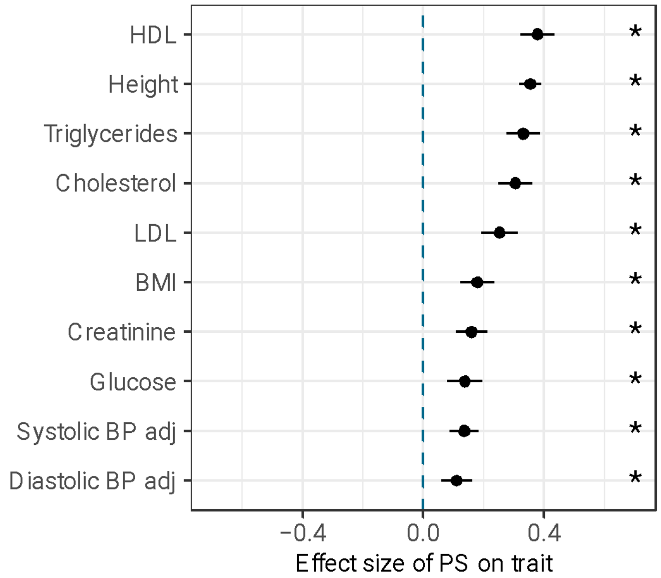

Fig. S57. Mixed model results for the polygenic score of each trait. The plot shows effect size estimates and confidence intervals from a mixed model analysis. Polygenic scores are computed using UKB GWAS summary statistics for each trait (SNPs significant at $P < 10^{-8}$), and are centered and scaled by 2 standard deviations. Asterisks show significance at FDR < 0.05 across traits and predictors analyzed. The plot shows effect size estimates and confidence intervals (1.96*SEM) from a mixed model analysis. n = 4625, 4607, 3664, 3613, 3714, 4605, 4605, 3665, 3665 and 3641 biologically independent samples were used for the analysis for Height, BMI, Triglycerides, Glucose, Creatinine, adjusted Diastolic blood pressure (DBP adj), adjusted Systolic Blood pressure (SBP adj), total Cholesterol, HDL and LDL cholesterol levels respectively.

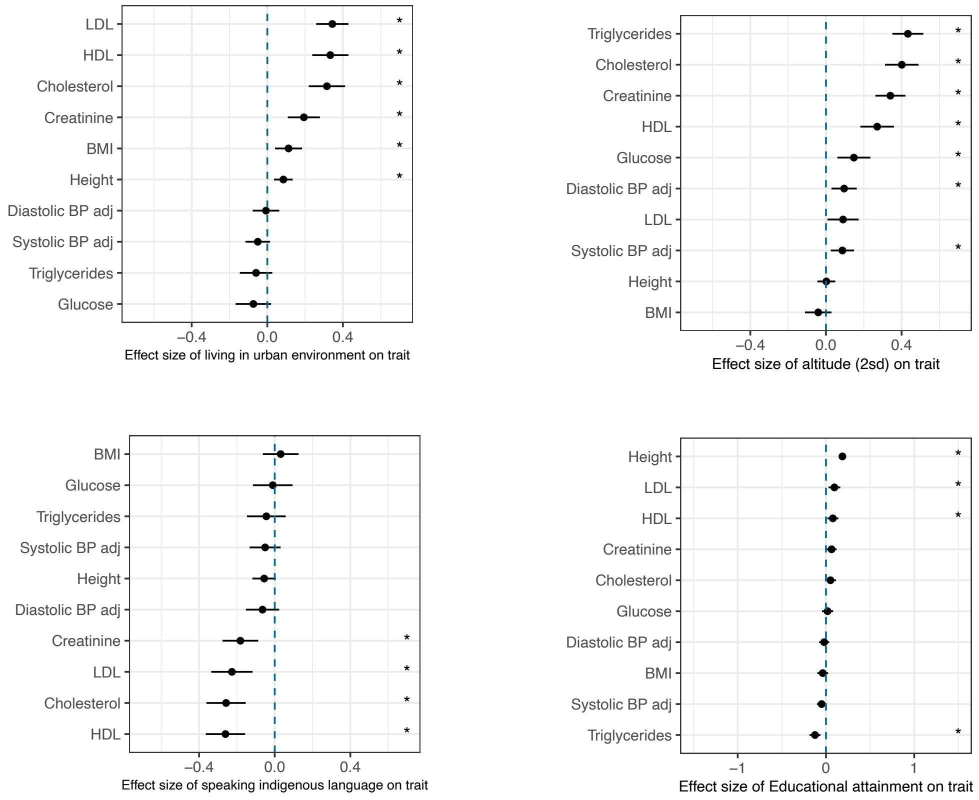

Figure S58. Mixed model results for the environmental predictors for each trait. The plot shows effect size estimates and confidence intervals from a mixed model analysis (see methods). Asterisks show significance at FDR < 0.05 across traits and predictors analyzed. Top row shows the analysis for living in an urban environment (left) and altitude (right). Bottom row shows the analysis for speaking an Indigenous language (left) and educational attainment (right). The plot shows effect size estimates and confidence intervals (1.96*SEM) from a mixed model analysis. n = 4625, 4607, 3664, 3613, 3714, 4605, 4605, 3665, 3665 and 3641 biologically independent samples were used for the analysis for Height, BMI, Triglycerides, Glucose, Creatinine, adjusted Diastolic blood pressure (DBP adj), adjusted Systolic Blood pressure (SBP adj), total Cholesterol, HDL and LDL cholesterol levels respectively.

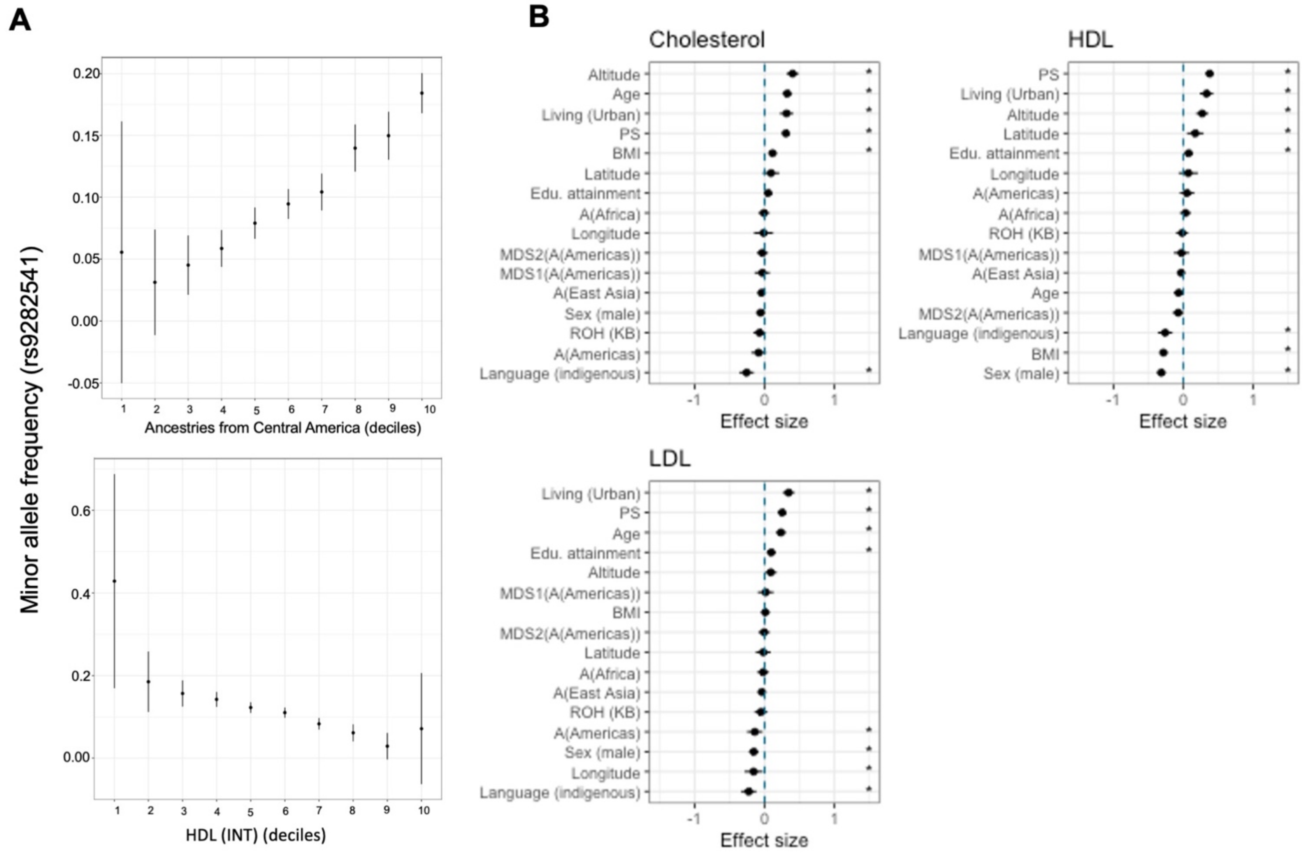

Fig. S59. Complex trait architecture of Cholesterol related traits in the MXB. (A) C230 allele in the ABCA1 gene shows higher frequency in individuals with higher ancestries from the Americas (top). Allele frequency is computed in deciles of individuals partitioned by their level of ancestries from the Americas as inferred from Admixture. Higher C230 allele frequency is found in individuals with lower HDL levels (bottom). This allele has previously shown to be present primarily in the Americas and associated with low HDL levels[43] similar to what is observed in the MXB. The plot shows allele frequencies and confidence intervals (1.96*SEM). n = X biologically independent samples were used for the analysis. (B) Cholesterol (top left panel), HDL (top right panel), and LDL (bottom panel) levels are explained by several genetic and environmental factors. The plot shows effect size estimates and confidence intervals (1.96*SEM) from a mixed model analysis. Asterisks show significance at FDR < 0.05 across all traits and predictors analyzed. n = 3665, 3665 and 3641 biologically independent samples were used for the analysis for total Cholesterol, HDL, and LDL cholesterol levels respectively. Notably, overall, ancestries from the Americas are associated with low LDL cholesterol levels ($\beta = -0.14$, p = 0.013), and speaking an Indigenous language is associated with low total ($\beta = -0.26$, p = 1.185×10^{-06}), HDL ($\beta = -0.26$, p = 1.009×10^{-06}) and LDL ($\beta = -0.23$, p = 4.928×10^{-05}) cholesterol levels. Despite the C230 effect, there is no association of overall ancestries from the Americas with HDL levels ($\beta = 0.05$, p = 0.324).

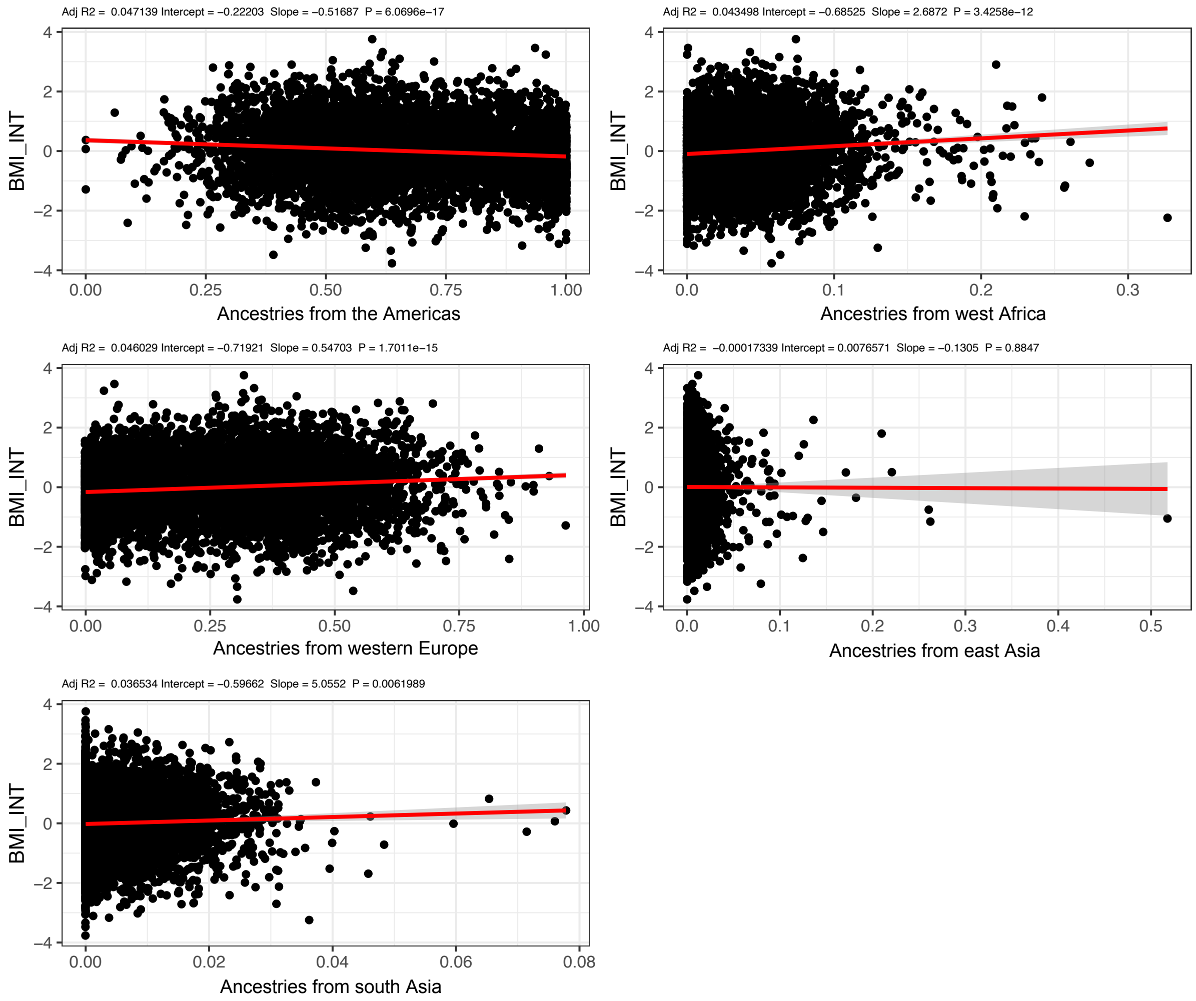

Fig. S60. Linear regression of BMI on ancestry proxies inferred from Admixture. BMI was inverse normal transformed same as for the mixed model analysis. A linear regression model is fit to the data, and the fitted slope, intercept and two-sided P-value are shown, along with the variance explained (R^2) by the model. Age and sex were included as covariates in the model. The fitted line is shown in red, with error bands showing 95% confidence intervals. We observe a significant negative slope of BMI with ancestries from the Americas (top row left) and a significant positive slope with ancestries from west Africa (top row right), western Europe (middle row left) and South Asia (bottom row). No significant slope is observed with ancestries from East Asia (middle row right).

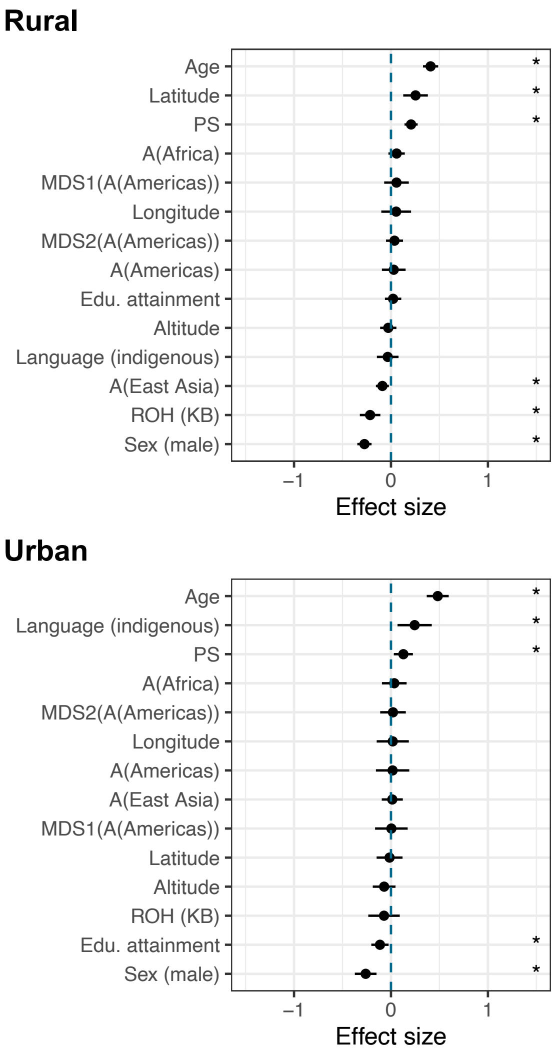

Fig. S61. Segmented analysis of BMI trait variation in MXB individuals from rural or urban localities. The plot shows effect size estimates and confidence intervals from a mixed model analysis. All quantitative predictors are centered and scaled by 2 standard deviations. Asterisks show significance at FDR < 0.05 across traits and predictors analyzed. Top panel shows the analysis for rural localities and bottom panel shows the analysis for urban localities. The plots show effect size estimates and confidence intervals (1.96*SEM) from a mixed model analysis. n = 3192 and 1415 biologically independent samples were used for the rural and urban analysis respectively.

Supplementary Tables

Characteristic		Number	(%)
**Age**			
	**20-39**	**3295**	**55.13**
	**40-59**	**1808**	**30.25**
	**60-79**	**763**	**12.77**
	**80+**	**111**	**1.86**
**Sex**			
	**Male**	**1820**	**30.45**
	**Female**	**4157**	**69.55**

Table S1. Age and sex distribution in the Mexican Biobank. Results shown for the quality control filtered dataset used for downstream analyses.

Anthropometric traits	Urine Biochemical traits	Health-related traits	Socioeconomic covariates	Familial Disease History	Health-related covariates	Blood biochemical traits
Height	pH	Systolic blood pressure (SBP and DBP)	Salary	Diabetes	Fertility	Cholesterol
BMI	Leukocytes	Diabetes treatment	Indigenous heritage	Hypertension	Contraceptive use	High-density Lipoproteins (HDL)
Weight	Nitrites	Diabetes diagnosis	Literacy	Cardiac disease	Alcohol use	Low-density Lipoproteins (LDL)
Foot size	Proteins	Vaccination history	Perceived health status	Inbreeding	Tobacco use	Triglycerides
Waist Circumference	Ketones	History of paludism	Access to healthcare			Creatinine
	Hemoglobin	History of Dengue	Disability			Glucose
		Renal disease indicators	Access to safe water			
		Tuberculosis diagnosis	Mosquito exposure			
		Arthritis	Proximity to pollution sources			
		Gout				
		Rheumatoid arthritis				
		Gout arthritis				
		Urinary tract infection (UTI)				
		High cholesterol				
		Hypertension				
		Genital_warts				
		Gonorrhoea				
		Inflammatory disease				
		Kidney stones				
		Prostate problems				
		Renal insufficiency				
		STD				

Table S2. List of anthropometric, disease and lifestyle variables in the Mexican Biobank. Additionally, a number of biochemical traits analyzed in this study were measured from blood samples.

	Indigenous	European	African	East Asian	South Asian
Baja California	52.8	40.7	5	1.1	0
Baja California Sur	47.6	45	4.7	2.1	0.6
Sonora	52.9	41.1	3.4	2.1	0.5
Chihuahua	41.7	51.2	5.1	1.3	0.8
Coahuila	53.1	40.2	5.5	0.7	0
Nuevo Leon	46.9	47	4.8	0.7	0.6
Tamaulipas	57.8	36.1	4.7	0.8	0.5
Sinaloa	43.3	50.1	4.8	1.2	0.6
Durango	51	41.5	6.1	0.9	0.5
Zacatecas	50.1	43.3	5.3	0.8	0.6
San Luis Potosi	74	22.1	3.1	0.5	0
Nayarit	48.8	43.5	6.1	1.1	0.5
Aguascalientes	52.1	41.4	5.3	0.7	0.5
Jalisco	50.1	43.6	4.9	0.8	0.6
Colima	50.4	42	5.9	1	0.7
Guanajuato	53.7	40.6	4.6	0.7	0
Queretaro	64.6	31	3.5	0.7	0
Veracruz	73.8	19.8	5.2	0.8	0
Michoacan	59.9	34.5	4.3	0.7	0.6
Hidalgo	77	20.4	1.8	0.5	0
Cd. Mexico	67.9	28.1	2.9	0.7	0
Edo. Mex	69	27.4	2.4	0.8	0
Tlaxcala	79.5	18.4	1.3	0	0
Guerrero	69.9	21.7	5.4	2.3	0.7
Puebla	82.7	14.9	1.6	0	0
Oaxaca	89.9	7.8	1.7	0	0
Morelos	71.3	24.3	3.2	0.5	0.6
Tabasco	73.5	19.1	6.1	0.6	0.5
Chiapas	84	12.2	2.9	0	0.5
Campeche	78.8	16.1	3.8	0.6	0.5
Yucatan	79.3	17.2	2.4	0	0.7
Quintana Roo	77.1	18.2	3.4	0.7	0.6

Table S3. Estimation of admixture proportions by state in MXB. The state with the highest ancestries from any source is highlighted.

Cultural group	ID	Mesoamerican region	Latitude	Longitude
Seri	SER	North of Mexico	29	-112.15
Tarahumara	TAR	North of Mexico	27.75	-107.17
Tepehuano	Tep	North of Mesoamerica	23.48	-104.39
Huichol	HUI	Occident of Mexico	21.17	-104.08
Nahua_Jaliso	NAJ	Occident of Mexico	19.5	-103.5
Purepecha	PUR	Occident of Mexico	19.75	-101.5
Totonac	TOT	Gulf of Mexico	20	-97.8
Nahua_Puebla	NXP	Gulf of Mexico	19.97	-97.62
Nahua_trio	NFM	Gulf of Mexico	19.93	-97.62
Nahua_Guerrero	NAG	Occident of Mexico	17.89	-99.13
Trique	TRQ	Oaxaca	17.18	-97.95
Zapotec	ZAP.N	Oaxaca	17.41	-96.69
Zapotec	ZAP.S	Oaxaca	17.23	-96.23
Mazatec	MAZ	Oaxaca	18.33	-96.33
Tzotzil	TZT	Mayan region	16.83	-92.67
Tojolabal	TOJ	Mayan region	16.5	-92
Lacandon	LAC	Mayan region	16.75	-91.25
Maya	MYA.Q	Mayan region	19.58	-88.58
Maya	MYA.C	Mayan region	20.37	-90.05
Maya	MYA.Y	Mayan region	21.17	-88.14

Table S4. NMDP cultural groups and their designation into Mesoamerican regions. This table is adapted from table S1 from Moreno-Estrada et al (2014)[12] with permission.

	Indigenous ancestries	African ancestries	European ancestries
**nROH**	**287675**	**429**	**23015**
**Local ancestry (Mb)**	**459820.79**	**2070.33**	**44006.19**
**nROH/localancestry (MB)**	**0.626**	**0.207**	**0.523**

Table S5. Number of ROH by local ancestry tracts.

Phenotype	N (cases/controls)
UTI	5723 (677/5046)
Gout	5722 (156/5566)
High cholesterol	5708 (345/5363)
Hypertension 1	4783 (748/4035)
Hypertension 2	5039 (1839/3200)
Rheumatoid arthritis	5720 (205/5515)
Diabetes	5734 (291/5443)
Arthritis	5721 (268/5453)
Height	5663
Weight	5681
BMI	5642
Glucose	4418
Fasting glucose	1361
Creatinine	4482
Triglycerides	4483
Cholesterol	4484
HDL	4484
LDL	4453
SBP	5737
SBP adj	5737
DBP	5737
DBP adj	5737

Table S6. Case and control numbers for each trait. N is the number of individuals for which information is available for each trait. Cases and controls are in parenthesis when applicable.

Trait	lead variant	Chr	Pos (b38)	Effect Allele	EAF	Infoscore	Beta	SE	P	Nearest Gene	GWAS catalogue
Cholesterol	rs7528419	1	109274570	A	0.801	0.999	0.135	0.024	1.440E-08	*CELSR2*	rs7528419
Cholesterol	rs57502215	16	56938507	G	0.789	0.980	0.135	0.024	1.017E-08	*HERPUD1*	
Cholesterol	rs56228609	16	56953853	C	0.673	0.997	-0.110	0.020	4.284E-08	*HERPUD1,CETP*	rs56228609
Cholesterol	rs118146573	16	56967026	G	0.854	0.995	0.155	0.027	6.030E-09	*CETP*	rs118146573
Creatinine	rs73579830	16	51055831	G	0.988	0.998	-0.456	0.082	3.304E-08	*LOC107984887*	
**HDL**	**rs2065412**	**9**	**104836459**	**C**	**0.451**	**0.999**	**-0.122**	**0.019**	**9.692E-11**	***ABCA1***	**rs2472386**
**HDL**	**rs9282541**	**9**	**104858554**	**G**	**0.883**	**0.987**	**0.219**	**0.030**	**1.645E-13**	***ABCA1***	**rs9282541**
**HDL**	**rs180326**	**11**	**116753987**	**G**	**0.475**	**1.000**	**-0.122**	**0.019**	**5.863E-11**	***BUD13***	**rs180326**
**HDL**	**rs200905431**	**11**	**116909318**	**GA**	**0.847**	**0.965**	**-0.166**	**0.026**	**2.050E-10**	***SIK3***	**rs188287950**
HDL	rs4784732	16	56860607	G	0.255	0.978	-0.124	0.022	1.677E-08	*NUP93*	rs78291913
**HDL**	**rs57502215**	**16**	**56938507**	**G**	**0.789**	**0.980**	**0.152**	**0.024**	**1.383E-10**	***HERPUD1***	
HDL	rs56129100	16	56941862	G	0.284	0.998	0.118	0.021	1.697E-08	*HERPUD1,CETP*	rs9938160
HDL	rs193695	16	56951244	A	0.262	0.998	-0.126	0.021	4.685E-09	*HERPUD1,CETP*	rs9989419
**HDL**	**rs56228609**	**16**	**56953853**	**C**	**0.673**	**0.997**	**-0.205**	**0.020**	**3.268E-24**	***HERPUD1,CETP***	**rs56228609**
**HDL**	**rs117427818**	**16**	**56976574**	**C**	**0.883**	**0.985**	**0.234**	**0.030**	**4.522E-15**	***CETP***	**rs117427818**
**LDL**	**rs7528419**	**1**	**109274570**	**A**	**0.802**	**0.999**	**0.194**	**0.025**	**2.766E-15**	***CELSR2***	**rs7528419**
LDL	rs66505542	11	116752497	TA	0.410	0.998	-0.112	0.020	1.971E-08	*BUD13*	rs66505542
**LDL**	**rs7412**	**19**	**44908822**	**C**	**0.975**	**0.991**	**0.408**	**0.063**	**1.103E-10**	***APOE***	**rs7412**
Triglycerides	rs947989	11	116702805	A	0.286	0.990	-0.111	0.020	2.729E-08	*BUD13,LINC02702*	rs1263056
**Triglycerides**	**rs66505542**	**11**	**116752497**	**TA**	**0.411**	**0.998**	**0.184**	**0.019**	**4.405E-23**	***BUD13***	**rs66505542**
**Triglycerides**	**rs5104**	**11**	**116821618**	**C**	**0.257**	**0.999**	**0.149**	**0.021**	**6.604E-13**	***APOC3***	**rs5141**
Triglycerides	rs440446	19	44905910	C	0.465	0.998	-0.106	0.018	4.719E-09	*APOE*	rs440446
Weight	rs4636755	12	23536367	T	0.200	0.985	-0.116	0.021	2.03E-08	*SOX5*	rs4246218
**Trait**	**lead variant**	**Chr**	**Pos (b38)**	**Effect Allele**	**EAF**	**Infoscore**	**OR**	**95%CI**	**P**	**Nearest Gene**	**GWAS catalogue**
Arthritis	rs12932003	16	81292633	A	0.600	0.990	0.572	0.47-0.7	4.86E-08	*BCO1*	
Hypertension	rs17607804	7	35350638	A	0.878	0.998	0.584	0.48-0.71	4.67E-08	*LOC401324*	

Table S7. Lead SNPs and most significant variant found in GWAS catalogue. The statistics were generated with whole-genome regression models as implemented in Regenie69 providing P-values, followed by Bonferroni correction for multiple tests. Lines in bold indicate loci passing Bonferroni correction.

	MXB-GWAS based				UKB-GWAS based			
	Imputed dataset		Genotyped dataset		Imputed dataset		Genotyped dataset	
	R	p	R	p	R	p	R	p
**Height**	0.033	0.17	0.059	0.016	0.025	0.29	0.15	2.8×10^{-10}
**BMI**	0.061	0.013	0.044	0.07	0.054	0.022	0.083	7×10^{-4}
**Triglycerides**	0.14	6.7×10^{-7}	0.087	0.0016	-0.029	0.27	0.064	0.019
**Cholesterol**	0.13	2.1×10^{-6}	0.058	0.033	0.067	0.01	0.034	0.22
**HDL**	0.13	4.7×10^{-6}	0.1	0.00017	0.026	0.32	0.0019	0.95
**LDL**	0.023	0.4	0.038	0.16	0.1	$9.5 \mathrm{X} 10^{-5}$	0.055	0.047
**Glucose**	0.12	2.3×10^{-5}	0.075	0.0069	0.044	0.097	0.019	0.5
**Creatinine**	0.17	3.9×10^{-10}	0.11	3.6×10^{-5}	0.00033	0.99	0.058	0.036
**Systolic blood pressure**	-0.01	0.68	0.016	0.5	-0.0043	0.85	0.043	0.075
**Diastolic blood pressure**	0.0086	0.72	0.052	0.032	-0.0016	0.95	0.051	0.035

Table S8. Assessment of polygenic score performance using MXB-GWAS or UKB-GWAS (SNPs significant at $p < 0.1$). We tested the prediction performance of polygenic scores by computing the Pearson's correlation (R and associated two-sided p-value) between the trait value and the polygenic score.

	MXB-GWAS based				UKB-GWAS based			
	**Imputed dataset**		**Genotyped dataset**		**Imputed dataset**		**Genotyped dataset**	
	**R**	**p**	**R**	**p**	**R**	**p**	**R**	**p**
**Height**	-0.00007	1	NA	NA	0.13	8.5×10^{-9}	0.16	1.2×10^{-10}
**BMI**	NA	NA	NA	NA	0.065	0.0053	0.086	0.00042
**Triglycerides**	0.13	4.8×10^{-6}	0.092	0.00081	0.15	1.3×10^{-8}	0.15	3.5×10^{-8}
**Cholesterol**	0.019	0.5	0.016	0.55	0.13	2.1×10^{-7}	0.12	1.2×10^{-5}
**HDL**	0.19	9×10^{-3}	0.17	1.5×10^{-10}	0.15	4.2×10^{-9}	0.21	2×10^{-14}
**LDL**	0.081	0.0033	0.13	3.5×10^{-6}	0.1	9.6×10^{-5}	0.11	3.2×10^{-5}
**Glucose**	NA	NA	NA	NA	0.032	0.22	0.052	0.06
**Creatinine**	NA	NA	NA	NA	0.076	0.0035	0.068	0.013
**Systolic blood pressure**	NA	NA	NA	NA	0.066	0.0042	0.077	0.0014
**Diastolic blood pressure**	0.044	0.069	NA	NA	0.07	0.0025	0.079	0.0012

Table S9. Assessment of polygenic score performance using MXB-GWAS or UKB-GWAS (SNPs significant at $p < 10^{-8}$). We tested the prediction performance of polygenic scores by computing the Pearson's correlation (R and associated two-sided p-value) between the trait value and the polygenic score.

Supplementary References

79. Rivera, J. A. *et al.* Epidemiological and nutritional transition in Mexico: rapid increase of non-communicable chronic diseases and obesity. *Public Health Nutr.* **5**, 113–122 (2002).

80. Feike de Jong, G. G. The Indigenous Voice of Mexico City. *Bloomberg News* (2018).
