# Supplementary material for: Mexican Biobank advances population and medical genomics of diverse ancestries
Source: Nature. 2023 Oct 11;622(7984):775–83. doi: 10.1038/s41586-023-06560-0 (PMC10600006; doi:10.1038/s41586-023-06560-0)
Supplement: Supplementary file 2 — Reporting Summary [file 41586_2023_6560_MOESM2_ESM.pdf]

nature portfolio

Corresponding author(s): Andres Moreno-Estrada, Mashaal Sohail, Lourdes Garcia-Garcia

Last updated by author(s): Jul 15, 2023

# Reporting Summary

Nature Portfolio wishes to improve the reproducibility of the work that we publish. This form provides structure for consistency and transparency in reporting. For further information on Nature Portfolio policies, see our Editorial Policies and the Editorial Policy Checklist.

## Statistics

For all statistical analyses, confirm that the following items are present in the figure legend, table legend, main text, or Methods section.

| n/a | Confirmed | |
|---|---|---|
| ☐ | ☒ | The exact sample size (*n*) for each experimental group/condition, given as a discrete number and unit of measurement |
| ☐ | ☒ | A statement on whether measurements were taken from distinct samples or whether the same sample was measured repeatedly |
| ☐ | ☒ | The statistical test(s) used AND whether they are one- or two-sided<br>*Only common tests should be described solely by name; describe more complex techniques in the Methods section.* |
| ☐ | ☒ | A description of all covariates tested |
| ☐ | ☒ | A description of any assumptions or corrections, such as tests of normality and adjustment for multiple comparisons |
| ☐ | ☒ | A full description of the statistical parameters including central tendency (e.g. means) or other basic estimates (e.g. regression coefficient) AND variation (e.g. standard deviation) or associated estimates of uncertainty (e.g. confidence intervals) |
| ☐ | ☒ | For null hypothesis testing, the test statistic (e.g. *F*, *t*, *r*) with confidence intervals, effect sizes, degrees of freedom and *P* value noted<br>*Give P values as exact values whenever suitable.* |
| ☐ | ☒ | For Bayesian analysis, information on the choice of priors and Markov chain Monte Carlo settings |
| ☐ | ☒ | For hierarchical and complex designs, identification of the appropriate level for tests and full reporting of outcomes |
| ☐ | ☒ | Estimates of effect sizes (e.g. Cohen's *d*, Pearson's *r*), indicating how they were calculated |

*Our web collection on statistics for biologists contains articles on many of the points above.*

## Software and code

Policy information about availability of computer code

**Data collection**

Our samples were genotyped on the Illumina's Multi-Ethnic Genotyping Array (MEGA). The design of this array was previously led by Christopher Gignoux and Genevieve Wojcik, co-authors on this manuscript. Several properties place the MEGAex array as the ideal choice for biobank genotyping. It captures 1,748,250 SNPs derived from admixed population studies, making it broadly applicable in diverse populations. Genome Studio was used to convert raw image files to plink files with raw genotype information. All SNPs were flipped to the forward strand, and duplicate SNPs were removed. For sites with missing chromosome number, physical position or both, we updated the map using the information in the SNP name or by mapping their rsID using dbSNP Build 151.
We use plink to remove all individuals with more than 5% missing genotype data, and all genotypes with more than 5% missing individuals. We restricted the analyses to only autosomes and removed all monomorphic SNPs. We restricted the analysis to only biallelic SNPs and removed all SNPs with ambiguous strand for all downstream analyses using SNPFLIP. All related individuals were detected using plink (--Z-genome --min 0.5) after pruning for LD (--indep-pairwise 50 5 0.5). A script was written to iteratively find nodes and remove related individuals to obtain the final QC-ed dataset.

**Data analysis**

EIGENSOFT (v7.2.1): https://github.com/dReichLab/EIG
Smartpca (part of Eigensoft v7.2.1): https://github.com/chrchang/eigensoft/tree/master/POPGEN
ADMIXTURE (v1.3.0): https://dalexander.github.io/admixture/
UMAP (repository downloaded Dec 2021): https://github.com/lmcinnes/umap
Archetypal analysis (repository downloaded Nov 2022): https://github.com/AI-sandbox/archetypal-analysis
GNOMIX (repository downloaded Oct 2021): https://github.com/AI-sandbox/gnomix
maas-MDS (repository downloaded Nov 2021): https://github.com/AI-sandbox/maasMDS/
shapeit (v2.17): https://mathgen.stats.ox.ac.uk/genetics_software/shapeit/shapeit.html
RFMIX (v2): https://github.com/slowkoni/rfmix

PCAmask (20131203): https://mybiosoftware.com/tag/pcamask
AdmixtureBayes (repository downloaded Jan 2023): https://github.com/svendvn/AdmixtureBayes
Refined-ibd (17Jan20): https://faculty.washington.edu/sguy/asibdne/
Merge-ibd-segments (17Jan20): https://faculty.washington.edu/sguy/asibdne/
Beagle (25Nov19): https://faculty.washington.edu/sguy/asibdne/
asIBDNe (19Sept19): https://faculty.washington.edu/sguy/asibdne/
Plink (v1.9): https://www.cog-genomics.org/plink/1.9/
Variant Effect Predictor (ensemble-vep-release-104): https://www.ensembl.org/info/docs/tools/vep/index.html
Regenie (v3.1.3): https://rgcgithub.github.io/regenie/
FINEMAP (v1.3): http://www.christianbenner.com/
FUMA (v1.4.1): https://github.com/Kyoko-wtnb/FUMA-webapp/
KING (v2.2.8): https://www.kingrelatedness.com/
Mxmaps (2020.1.1.9000): https://www.diegovalle.net/mxmaps/
Genesis (Release 3.17): https://www.bioconductor.org/packages/release/bioc/html/GENESIS.html
lme4qtl (development version): https://github.com/variani/lme4qtl

For manuscripts utilizing custom algorithms or software that are central to the research but not yet described in published literature, software must be made available to editors and reviewers. We strongly encourage code deposition in a community repository (e.g. GitHub). See the Nature Portfolio guidelines for submitting code & software for further information.

# Data

Policy information about availability of data

All manuscripts must include a data availability statement. This statement should provide the following information, where applicable:

- Accession codes, unique identifiers, or web links for publicly available datasets
- A description of any restrictions on data availability
- For clinical datasets or third party data, please ensure that the statement adheres to our policy

The dataset for the 6,057 newly genotyped individuals from the MX biobank project are available at the European Genome-phenome Archive (EGA) through a Data Access Agreement with the Data Access Committee (EGA accession number for study: EGAS00001005797; Datasets: EGAD00010002361 “Mexican_Biobank_Genotypes” and EGAD00001008354 “Mexican Biobank 50 Genomes”). GWAS summary statistics generated as part of this study are available at: https://doi.org/10.5281/zenodo.7420254. Variant Effect Predictor (VEP, https://asia.ensembl.org/info/docs/tools/vep/index.html) was used to annotate the effect of a variant using the humdiv database. 1000 Genomes data was accessed from: http://ftp.1000genomes.ebi.ac.uk/vol1/ftp/release/20130502/. HGDP data was downloaded from: https://rosenberglab.stanford.edu/hgdpsnpDownload.html. Pan-ancestry GWAS summary statistics from UK Biobank were downloaded from: https://pan.ukbb.broadinstitute.org/docs/summary.

# Human research participants

Policy information about studies involving human research participants and Sex and Gender in Research.

| | |
|---|---|
| Reporting on sex and gender | Self-reported sex was collected as part of the survey, and sex was also identified using genetic plink analysis. There are self-reported 1844 males and 4213 females in the entire dataset. There were a few cases (249) where plink reports an ambiguous sex. For our complex trait analyses, we used self-reported sex as a covariate. Prior to GWAS and polygenic score analyses, individuals with mismatched sex or IBD>0.9 were removed. |
| Population characteristics | Trained personnel conducted the interviews. Information was collected on household and sociodemographic characteristics, current health status, health care service usage, and behavioral aspects of participants. The genotyped and QC'ed data is 30.45% Male and 69.55% Female. The age distribution is as follows: 20-39 (55.13%), 40-59 (30.35%), 60-79 (12.77%) and 80+ (1.86%). The dataset is ~70% from rural areas and ~30% from urban areas. |
| Recruitment | Since 1988, Mexico has established periodical health surveys (ENSA) for surveillance of Mexican population-based health and nutrition metrics. In this study, we use data and samples collected by the National Institute of Public Health (INSP) from the survey performed in 2000, the ENSA 2000. This survey was a probabilistic, multi-stage, stratified, cluster household survey conducted by the Mexican Secretariat of Health from November 1999 to June 2000. Research design and methods have been described elsewhere. Participants were randomly selected in order to be representative of the civilian, non-institutionalized Mexican population, at the state and national levels. |
| Ethics oversight | The ENSA 2000 was carried out following the strictest ethical principles and in accordance with the Helsinki Declaration of Human Studies. Informed consent was obtained from all participants. Extracted DNA has been stored and maintained at the National Institute of Public Health (Cuernavaca, Mexico), and samples were genotyped and analyzed at the Advanced Genomics Unit of CINVESTAV (Irapuato, Mexico) through a collaborative institutional agreement. The project was reviewed and approved by the Research Ethics Committee and the Biosafety Committee of the National Institute of Public Health (IRB approvals CI: 1479 and CB: 1470). For the present project, personally identifiable data was removed from the data set. |

Note that full information on the approval of the study protocol must also be provided in the manuscript.

# Field-specific reporting

Please select the one below that is the best fit for your research. If you are not sure, read the appropriate sections before making your selection.

☒ Life sciences ☐ Behavioural & social sciences ☐ Ecological, evolutionary & environmental sciences

For a reference copy of the document with all sections, see nature.com/documents/nr-reporting-summary-flat.pdf

# Life sciences study design

All studies must disclose on these points even when the disclosure is negative.

| | |
|---|---|
| Sample size | No statistical methods were used to predetermine sample size. The sample size was selected before analysis was begun based on available samples and budgetary constraints for genotyping. We sought to include sufficient sample to power statistical comparisons. |
| Data exclusions | We use plink to remove all individuals with more than 5% missing genotype data, and all genotypes with more than 5% missing individuals. We restricted the analyses to only autosomes and removed all monomorphic SNPs. We restricted the analysis to only biallelic SNPs and removed all SNPs with ambiguous strand for all downstream analyses using SNPFLIP. All related individuals were detected using plink (--Z-genome --min 0.5) after pruning for LD (--indep-pairwise 50 5 0.5). A script was written to iteratively find nodes and remove related individuals to obtain the final QC-ed dataset. Prior to GWAS and polygenic score analyses, individuals with mismatched sex or IBD>0.9 were removed. |
| Replication | No experiments were conducted, so replication of experimental results is not relevant. |
| Randomization | For some analyses where covariates were relevant such as the complex trait analyses, they were controlled for in a mixed model framework using lme4qtl. For the GWAS analysis, individuals were either allocated into cases and controls groups for binary traits or analyzed in a single group for quantitative traits. In both cases, covariates such as the genetic relationship matrix, principal components, age and sex were controlled for using a novel machine-learning method called REGENIE for fitting a whole-genome regression model. For all other analyses, either all individuals were used, or a subset of individuals with high proportion of ancestries from the Americas (as inferred using Admixture) were used when we wanted to focus on patterns in specifically the indigenous genetic background. |
| Blinding | Blinding was not relevant to this study, as it is not a clinical trial but rather a descriptive population genetics analysis and association study. The individuals were not given a drug for which the effects were evaluated, thus making blinding irrelevant. |

# Reporting for specific materials, systems and methods

We require information from authors about some types of materials, experimental systems and methods used in many studies. Here, indicate whether each material, system or method listed is relevant to your study. If you are not sure if a list item applies to your research, read the appropriate section before selecting a response.

## Materials & experimental systems

| n/a | Involved in the study |
|---|---|
| ☒ | ☐ Antibodies |
| ☒ | ☐ Eukaryotic cell lines |
| ☒ | ☐ Palaeontology and archaeology |
| ☒ | ☐ Animals and other organisms |
| ☒ | ☐ Clinical data |
| ☒ | ☐ Dual use research of concern |

## Methods

| n/a | Involved in the study |
|---|---|
| ☒ | ☐ ChIP-seq |
| ☒ | ☐ Flow cytometry |
| ☒ | ☐ MRI-based neuroimaging |
